# Carbon quantum dot modified clinochlore as a green support for the formation and stabilization of palladium nanoparticles in aqueous Suzuki–Miyaura coupling

**DOI:** 10.1039/d6ra03494b

**Published:** 2026-07-24

**Authors:** Mohammad Gholinejad, Setare Jafarpour, José M. Sansano

**Affiliations:** a Department of Chemistry, Institute for Advanced Studies in Basic Sciences (IASBS) Zanjan 45137-66731 Iran gholinejad@iasbs.ac.ir; b Research Center for Basic Sciences & Modern Technologies (RBST), Institute for Advanced Studies in Basic Sciences (IASBS) Zanjan 45137-66731 Iran; c Departamento de Química Orgánica, Instituto de Síntesis Orgánica, and Centro de Innovación en Química Avanzada (ORFEO-CINQA), Universidad de Alicante 03690 Alicante Spain

## Abstract

A sustainable and efficient heterogeneous palladium catalyst was developed by supporting palladium nanoparticles on clinochlore as a naturally abundant clay mineral modified with carbon quantum dots (CQDs). The clinochlore-CQD support was synthesized *via* a straightforward hydrothermal method using citric acid and urea as the CQD precursors. The presence of CQDs provides an abundance of surface functional groups, facilitating the effective reduction and stabilization of Pd(0) from a PdCl_2_ precursor. Comprehensive characterization of the clinochlore-CQDs@Pd nanocomposite confirmed that the lamellar structure of the clinochlore remained intact and Pd nanoparticles homogeneously distributed without aggregation. The catalytic performance of the composite was evaluated in the Suzuki–Miyaura cross-coupling of aryl halides with arylboronic acids. The catalyst exhibited high activity toward a broad range of aryl iodides, bromides, and chlorides, delivering biaryl products in high to excellent yields under mild conditions in aqueous media. Furthermore, the key role of CQDs in enhancing palladium stabilization and catalytic efficiency was demonstrated. The catalyst was successfully recycled over several runs, with post-reaction characterization confirming its robust structural stability.

## Abstract 2

A sustainable and efficient heterogeneous palladium catalyst for Suzuki–Miyaura coupling reaction was developed by supporting palladium nanoparticles on clinochlore as a naturally abundant clay mineral modified with carbon quantum dots (CQDs).

## Graphical abstract

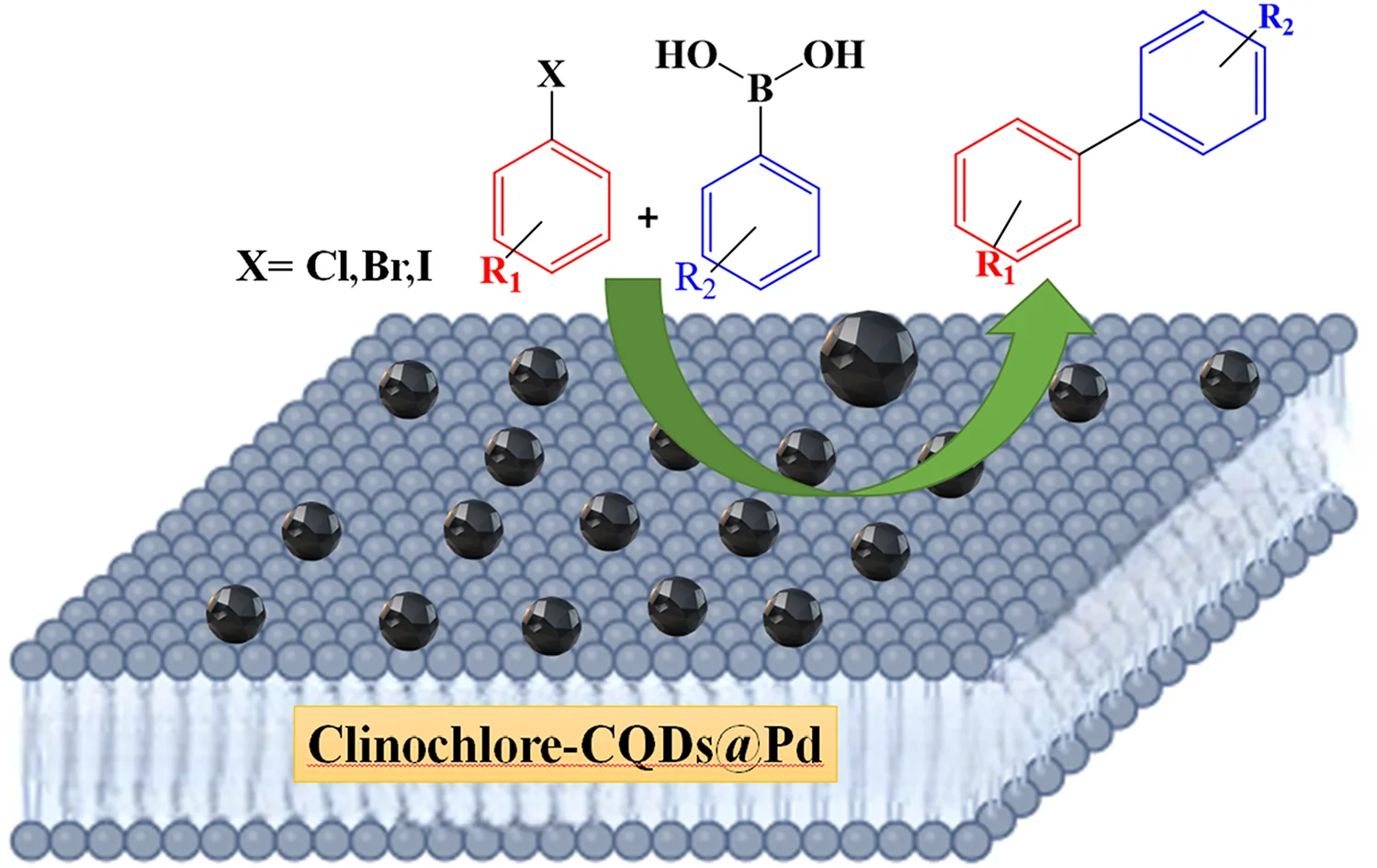

## 1 Introduction

Coupling reactions include a main class of transformations with wide industrial and academic importance, particularly for the formation of carbon–carbon bonds. Among different coupling reactions, palladium catalyzed Suzuki–Miyaura cross-coupling reaction, which is the reaction between aryl halides or pseudo halides with organoboron compounds in the presence of a base, is extensively applied for the preparation of biaryl products.^1–3^ The general applicability of the Suzuki–Miyaura reaction resulted from the stability and low toxicity of organoboron reagents, milder reaction conditions, requirement for relatively low catalyst loadings, and the easy removal of boron-containing byproducts. In recent years, efforts have increasingly focused on developing more sustainable and green reaction conditions such as using safer and more stable solvents, and green ligands and supports. Furthermore, it has become well recognized that catalyst choice plays a crucial role in determining the efficiency and selectivity of the reaction.^1–6^ During recent decades, numerous homogeneous or heterogeneous palladium catalysts have been applied in the Suzuki–Miyaura reaction.^7–10^ Despite the homogeneous catalysts typically exhibit high catalytic activity due to the availability of frequent active sites, their separation and recovery from the reaction medium remain challenging. On the other hand, heterogeneous catalysts can be easily recovered and recycled, while they often suffer from disadvantages such as lower catalytic activity.^11–14^ Recently, growing interest has been focused to the use of green and naturally abundant materials for the preparation of heterogeneous catalysts.^15^ Among the various materials employed as catalyst supports, mineral clays have attracted considerable attention due to their natural abundance, low cost, environmental compatibility, and structural versatility. Clay minerals have high thermal stability, high mechanical strength, layered or porous structures, and tunable surface chemistry which allow effective incorporation and stabilization of catalytically active metals.^16–18^

Clinochlore is a member of chlorite phyllosilicate minerals which is widely distributed in altered oceanic crust and low-grade metamorphic environments. It is typically formed through the secondary transformation of primary mafic minerals during hydrothermal and metamorphic processes. Clinochlore has layered crystal structure and complex chemical composition which generally showed as (Mg, Mn, Fe)_3_(Si, Al)_4_O_10_(OH)_2_(Mg, Mn, Fe, Al)_3_(OH)_6_. This clay exhibits high structural stability and favorable surface properties which enable effective synergistic interaction with supported metal species and facilitate their uniform dispersion on the mineral surface.^19,20^ In addition, the natural availability, low production cost, and environmental compatibility of clinochlore make it an attractive alternative for using as a promising mineral support for the development of sustainable heterogeneous catalytic systems.

Carbon quantum dots (CQDs) are a class of carbon-based nanomaterials that are environmentally benign, nontoxic and readily synthesizable *via* simple methods. They exhibit unique properties such as excellent photostability and fluorescence, which enable a wide range of applications. The presence of surface carboxyl and hydroxyl functional groups provides carbon dots with high water solubility and good biocompatibility, making them promising supports for active metal catalyst immobilization.^21–23^ One of the interesting applications of C-dots was described by Dey *et al.*, as a reducing agent for the synthesis of Pd nanoparticles.^24^ However fewer attentions have been paid for using CQDs in design of heterogeneous catalysts^25–29^ and to the best of our knowledge, the modification of minerals such as clinochlore with C-dots for the stabilization of palladium nanoparticles in coupling reactions remains unexplored. Herein, we report the synthesis of a clinochlore-based substrate, functionalized with carbon quantum dots and decorated with palladium nanoparticles (clinochlore-CQDs@Pd). Furthermore, its catalytic performance was rigorously evaluated in the Suzuki–Miyaura cross-coupling reaction.

## 2 Results and discussion

### 2.1. Catalyst characterization

**Scheme 1:**
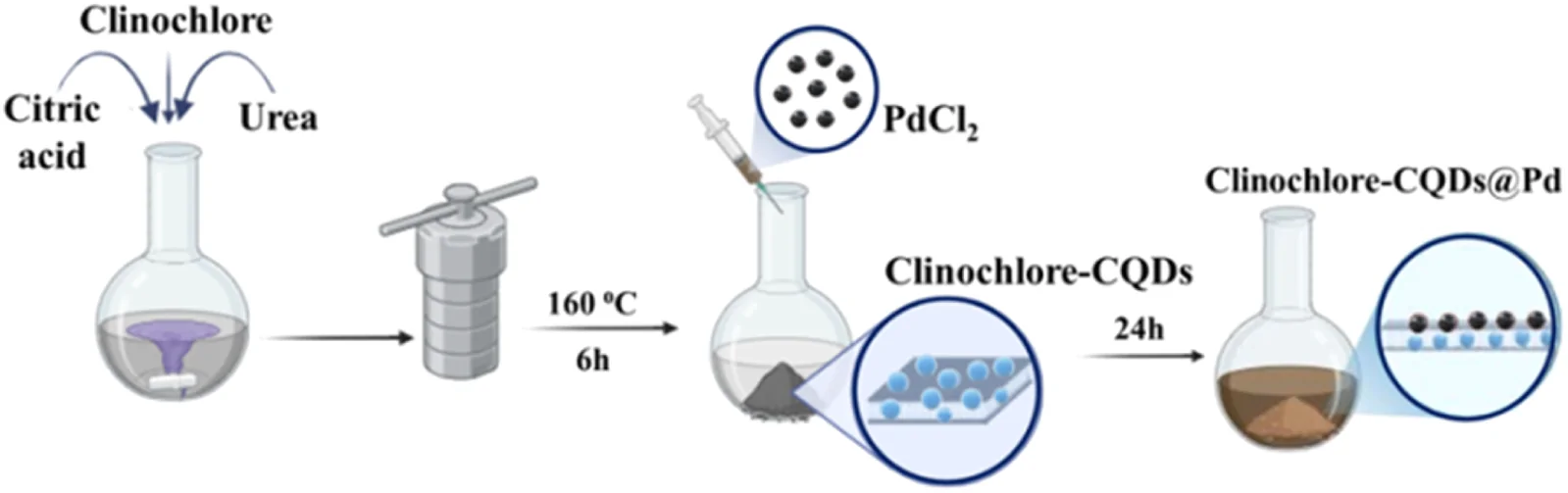
Step for the preparation of clinochlore-CQDs@Pd.

The steps for preparing clinochlore-CQDs@Pd are summarized in Scheme 1. Briefly, clinochlore was modified with CQDs, and the resulting clinochlore-CQDs material was subsequently treated with PdCl_2_. The loading of Pd in the obtained material was determined by atomic absorption spectroscopy (AAP) analysis to be 0.07 mmol g^−1^.

**Fig. 1:**
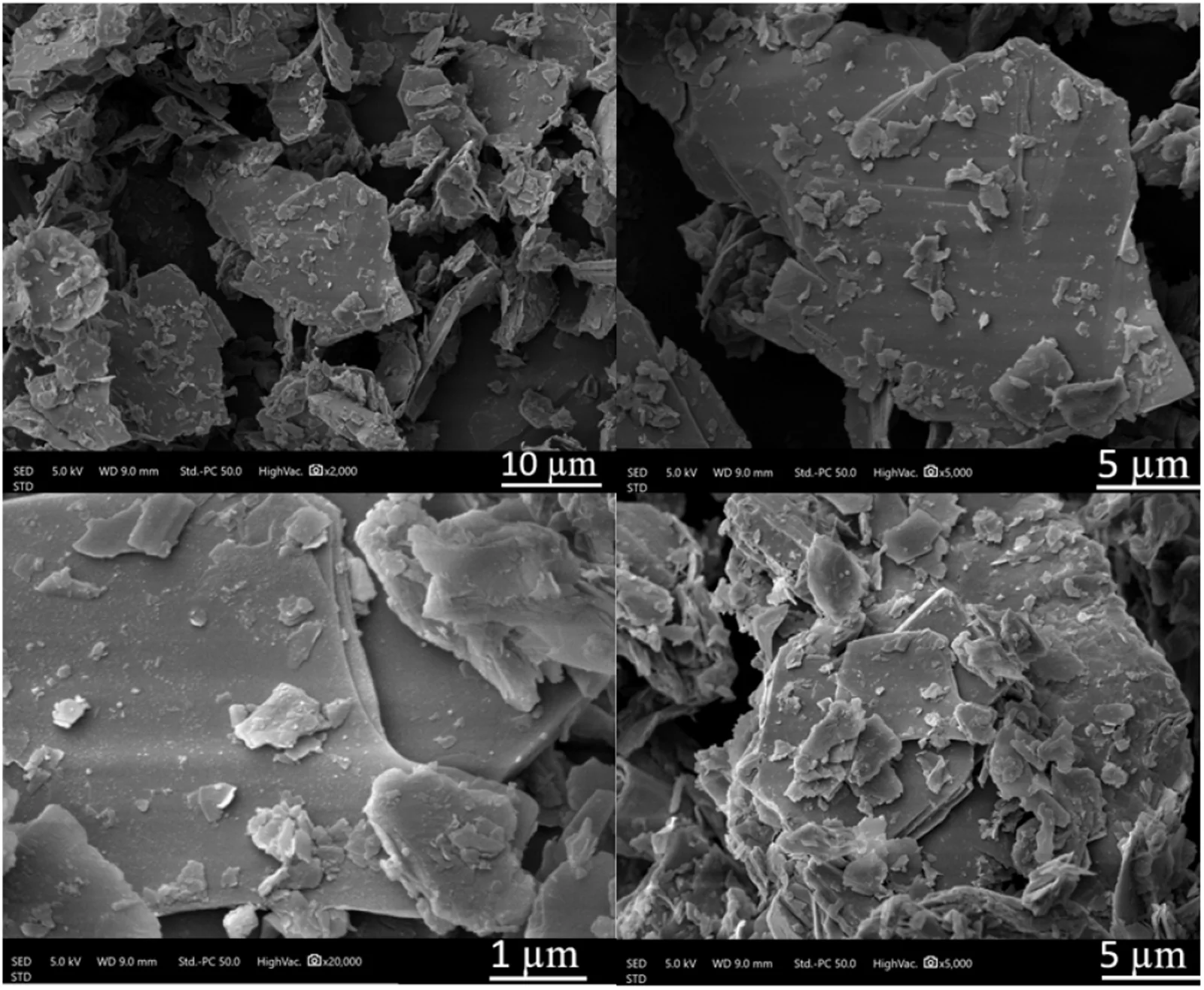
SEM images of clinochlore-CQDs@Pd.

The scanning electron microscope (SEM) images reveal the characteristic lamellar and plate-like morphology of clinochlore indicating that the layered silicate structure is preserved after modification (Fig. 1).

**Fig. 2:**
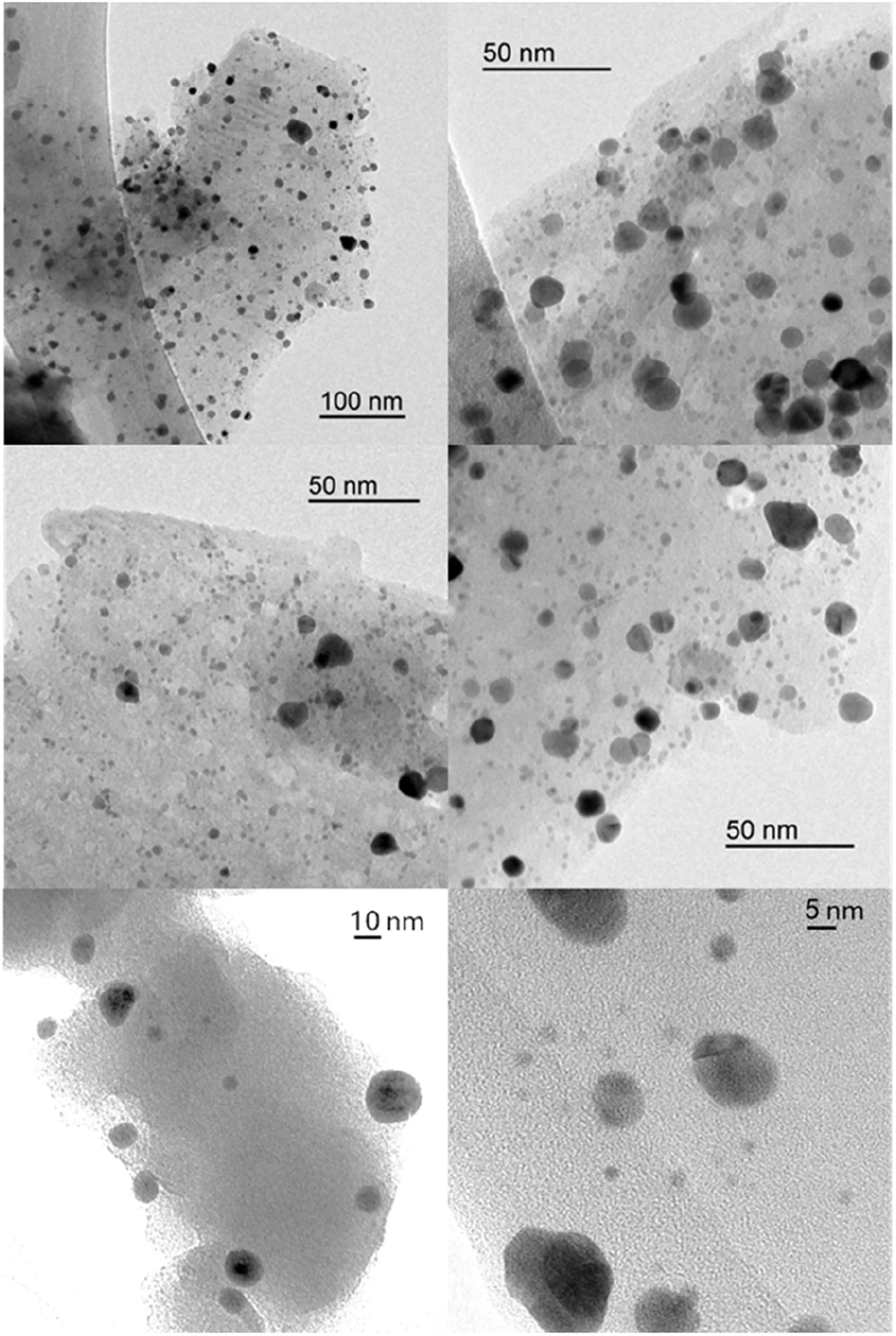
TEM images of clinochlore-CQDs@Pd.

The transmission electron microscopy (TEM) images provide the formation and dispersion of Pd nanoparticles on the CQD-modified clinochlore support. Palladium nanoparticles are clearly visible and are homogeneously distributed over the lighter clinochlore matrix, indicating effective stabilization by the CQDs (Fig. 2).

**Fig. 3:**
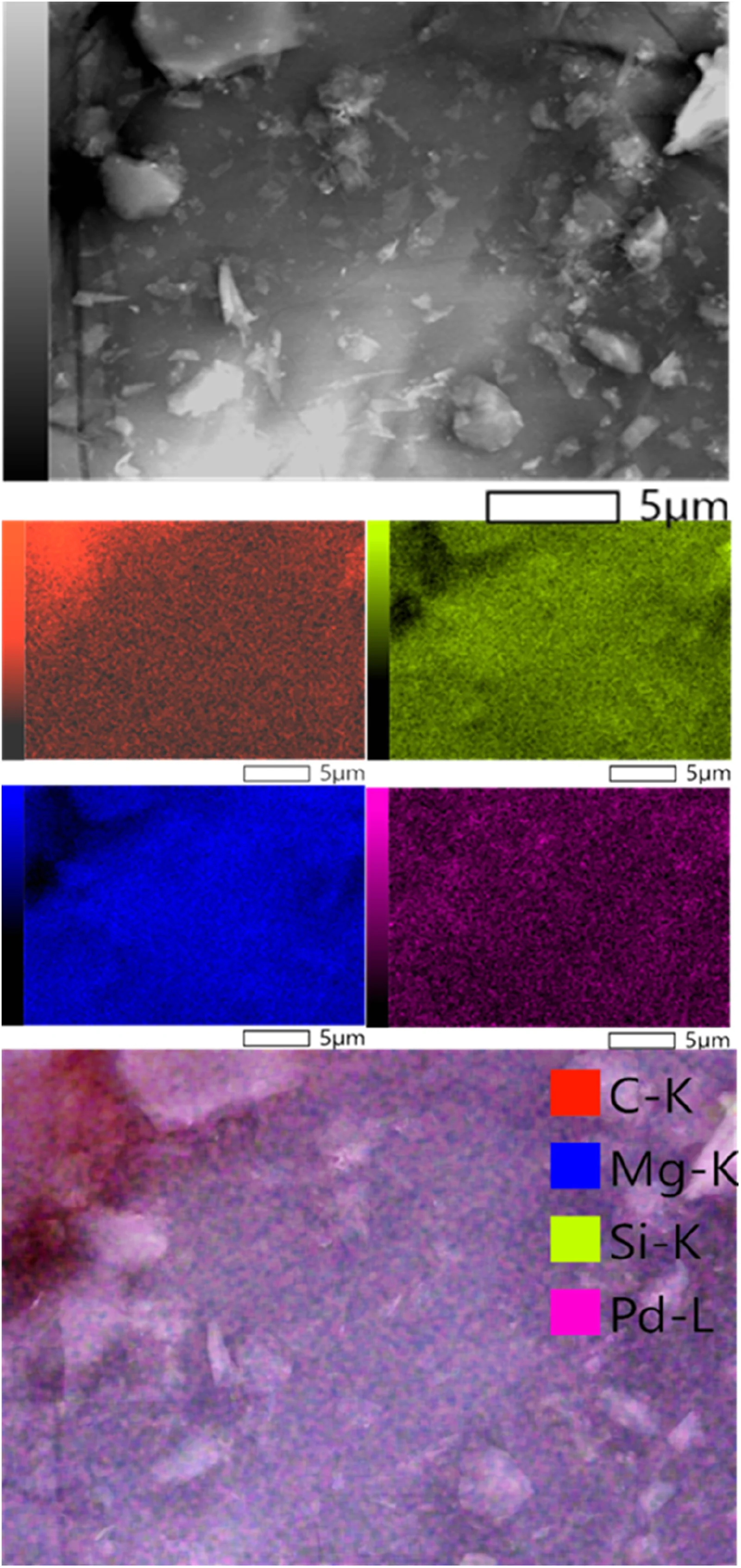
SEM mapping images of clinochlore-CQDs@Pd.

The SEM mapping results disclose the uniform presence of Si and Mg as well as regular distribution of carbon proving the successful coating of the clinochlore with carbon quantum dots. Also, the Pd mapping image reveals a well-dispersed distribution of palladium nanoparticles within the CQD-modified clinochlore without noticeable aggregation (Fig. 3).

The EDX spectrum of the clinochlore-CQDs@Pd displays presence of O, Mg, Al, and Si, as characteristic elements of the clinochlore mineral. The presence of a C and Pd indicates the successful coating of clinochlore with carbon quantum dots and effective immobilization of palladium nanoparticles (Fig. S1).

**Fig. 4:**
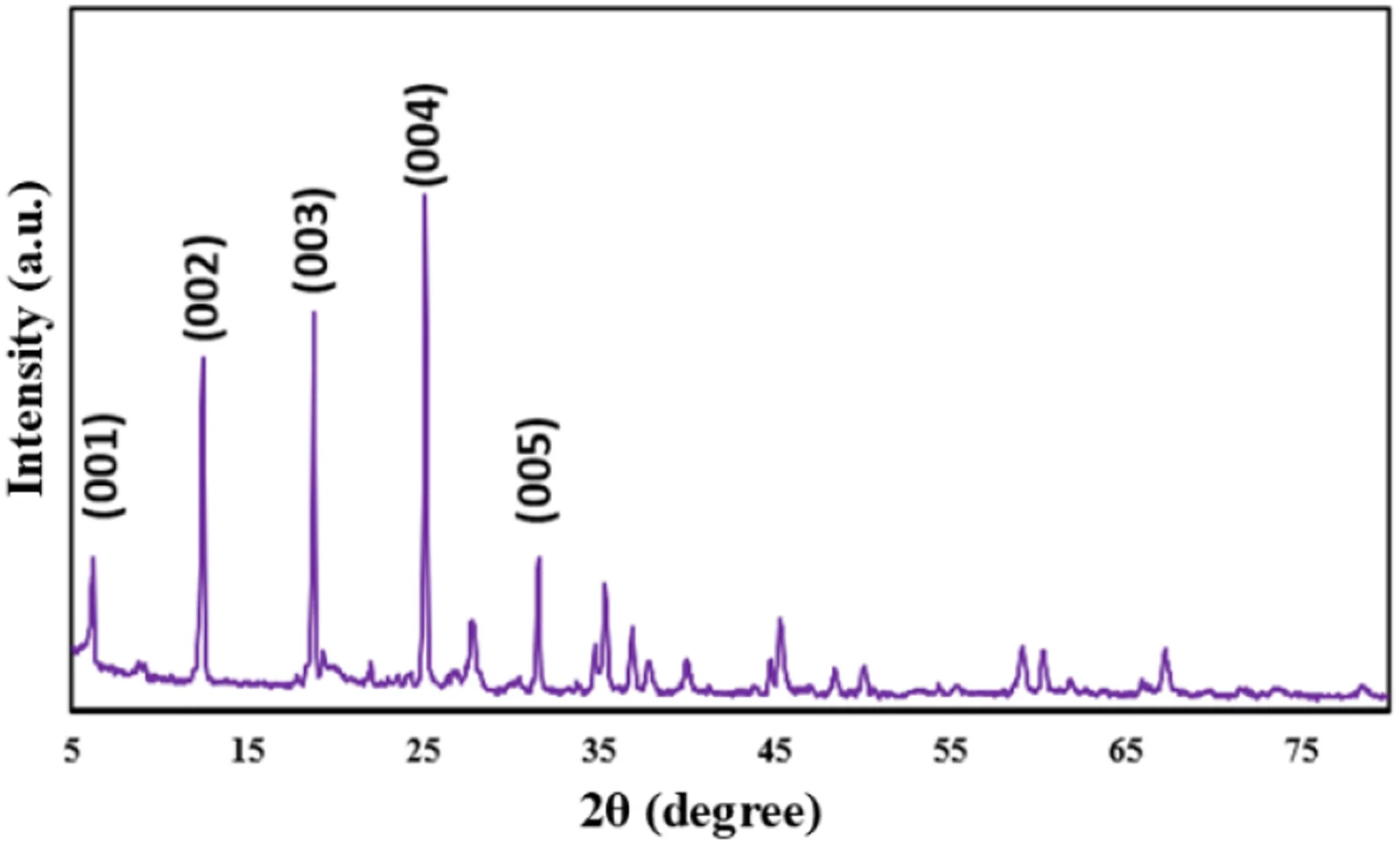
XRD pattern of the clinochlore-CQDs@Pd.

The X-ray powder diffraction (XRD) pattern of clinochlore-CQDs@Pd was studied and the results showed good agreement with the standard diffraction pattern of clinochlore (PDF no. 07-0078).^30^ The characteristic diffraction peaks corresponding to the (001), (002), (003), (004), and (005) crystal planes were observed at 2*θ* = 6.2°, 12.5°, 18.8°, 25.1°, and 31.6°, respectively, confirming the preservation of the clinochlore crystalline structure in the clinochlore-CQDs@Pd nanocomposite. However, because of overlapping of Pd reflections at 2*θ* = 40.1°, 46.7°, and 68.2° with the strong diffraction peaks of clinochlore, the characteristic diffraction peaks of Pd were not observed (Fig. 4)^20^

**Fig. 5:**
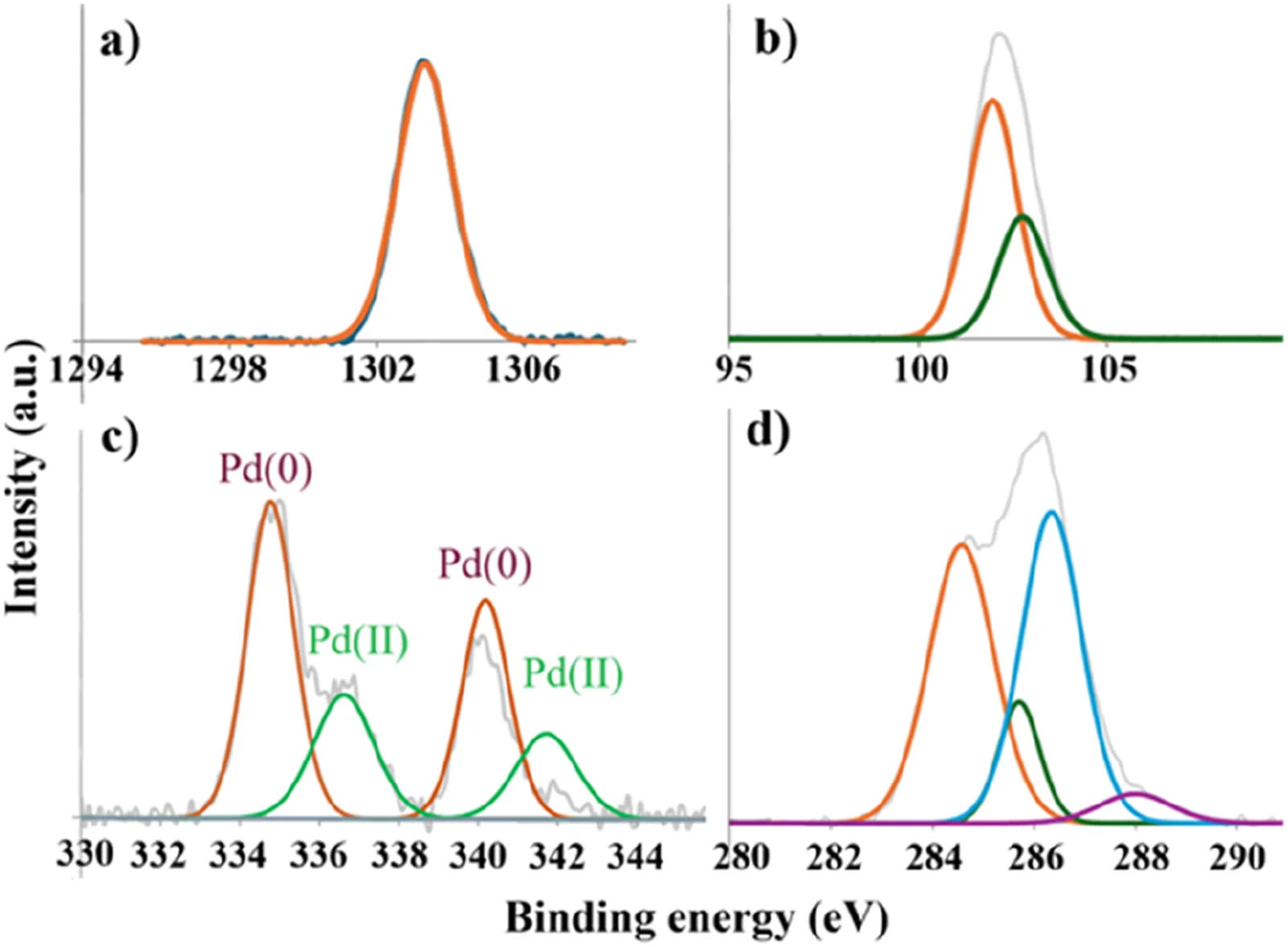
XPS spectra of clinochlore-CQDs@Pd in (a) C 1s, (b) Si 2p, (c) Pd 3d, (d) C 1s regions.

X-ray photoelectron spectroscopy (XPS) was used to study the chemical states of Mg, Si, Pd, and C in the clinochlore-CQDs@Pd (Fig. 5). The Mg 1s spectrum exhibited a peak at 1303.3 eV, which is attributed to Mg–O species, confirming the presence of MgO (Fig. 5a).^31^ Also, Si 2s spectrum showed two peaks at 101.6 and 102.7 eV, corresponding to SiO_*x*_ species of clinochlore (Fig. 5b).^32,33^

The Pd 3d spectrum displayed two spin–orbit doublets assigned to Pd(0) and Pd(ii) species in which peaks at 334.9 and 340.2 eV are associated with metallic Pd(0), while peaks at 336.6 and 341.8 eV correspond to Pd(ii). Notably, the majority of palladium is present in the zerovalent state confirming significant role of CQDs in supporting the reduction of Pd(ii) to Pd(0) (Fig. 5c).^21–25^ C 1s region showed at 284.6, 285.4, 286.4, and 288.1 eV, which are characteristic for C=C/C–C, C–N, C–O, and C=O forms of carbon in CQDs, respectively (Fig. 5d).^28,34^

**Fig. 6:**
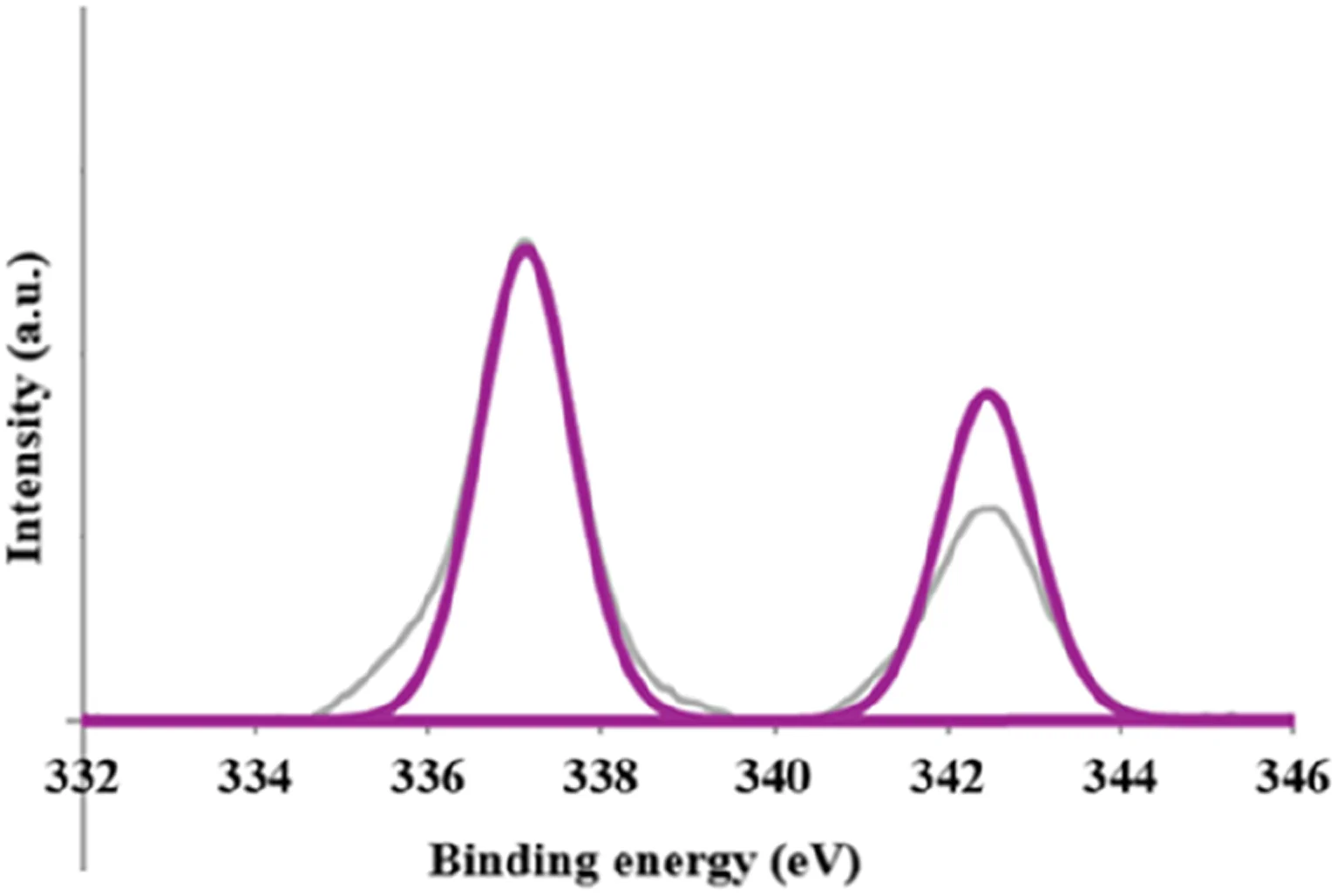
XPS spectra of clinochlore@Pd without CQDs in Pd 3d region.

To elucidate the specific role of CQDs in the formation of metallic Pd species, unmodified clinochlore was employed as a control support, resulting in the preparation of clinochlore@Pd. XPS analysis of this material in the Pd 3d region revealed that palladium existed exclusively in the Pd(ii) oxidation state. This confirms the indispensable role of CQDs as reducing and stabilizing agents in the formation of Pd(0) (Fig. 6).

The nitrogen adsorption–desorption isotherm of the clinochlore-CQDs@Pd exhibits a Type II isotherm. This adsorption behavior suggests that the clinochlore-CQDs@Pd possess a predominantly layered structure with unrestricted monolayer–multilayer adsorption. The Brunauer–Emmett–Teller (BET) surface area analysis revealed a specific surface area of approximately 12 m^2^ g^−1^ (Fig. S2).

### 2.2. Catalytic performance

The catalytic activity of the prepared clinochlore-CQDs@Pd was evaluated in the Suzuki–Miyaura cross-coupling reaction of aryl halides. Initially reaction of 4-bromoanisole with phenylboronic acid was selected as a standard reaction. Based on our previous studies and reported literature,^21,22^ we chose equal mixture of EtOH and water as a reaction solvent and K_2_CO_3_ as a base and studied effect of catalyst amount, reaction time and temperature (Table 1).

**Table 1:** Optimization of the reaction condition in the Suzuki reaction between 4-bromoanisole and phenylboronic acid

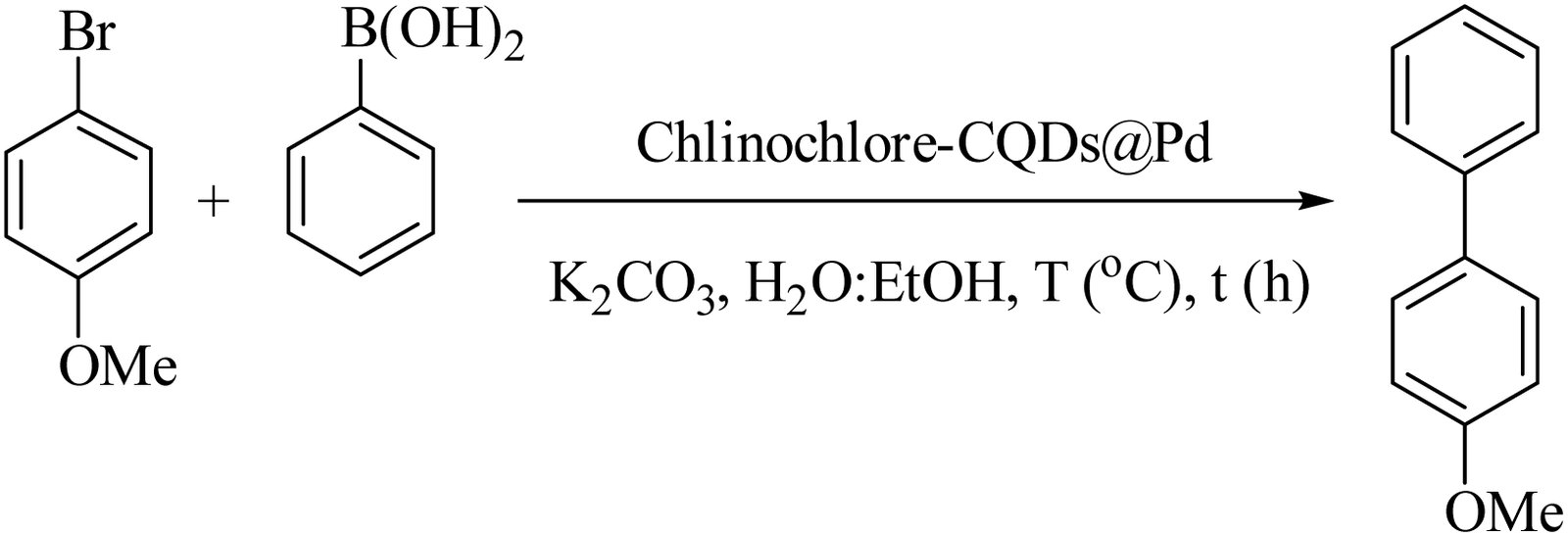				
Entry	Amount(mol% of Pd)	*T* (°C)	*t* (h)	Yield (%)^a^
1	0.035	60	6	15
2	0.07	60	6	50
3	0.1	60	6	96
4	0.17	60	6	97
5	0.1	60	1	52
6	0.1	60	2	65
7	0.1	60	3	75
8	0.1	60	5	88
9	0.1	60	24	98
10	0.1	25	6	20
11	0.1	50	6	60
12	0.1	60	6	45^b^
13	0.1	60	6	3^c^

a Yields determined by GC.

b Reaction performed using clinochlore-CQDs as a catalyst.

c Reaction performed using clinochlore-Pd as a catalyst.

The effect of catalyst loading on the reaction efficiency was first investigated (Table 1). Results showed that increasing the amount of clinochlore-CQDs@Pd from 0.035 mol% to 0.17 mol% resulted in a significant enhancement of the yield from 15% to 97% within 6 h in which 0.1 mol% of catalyst afforded an excellent yield (96%) while further increase in catalyst to 0.17 mol% had only a minimal effect (Table 1, entries 1–4). The influence of reaction time was then examined using 0.1 mol% of catalyst at 60 °C. Results indicated that shorter reaction times led to lower yields, whereas extending the reaction time up to 24 h did not result in a substantial improvement compared to 6 h (Table 1, entries 5–9), demonstrating that 6 h is sufficient to reach almost complete conversion. When the reaction was performed at room temperature using 0.1 mol% of catalyst, only a low yield (20%) was obtained after 6 h (Table 1, entry 10), while increasing the temperature to 50 °C the yield was improved to 60% (Table 1, entry 11). To highlight the role of carbon quantum dots in catalyst efficiency, a control experiment was designed in which clinochlore-Pd was prepared with same amount of Pd affording very low yield (Table 1, entry 12). Also, clinochlore-Pd exhibited significantly lower catalytic activity, while the reaction did not proceed in the presence of clinochlore-CQDs alone (Table 1, entries 13).

To ensure the selection of the most appropriate solvent several tests, including H_2_O : EtOH mixtures, EtOH, H_2_O, THF, and DMF, was investigated at 60 °C. Results indicated that the highest reaction yield was obtained when a 1 : 1 mixture of H_2_O and EtOH was employed. (Table S1, entries 1–7).

Furthermore, results showed that other bases such as Na_2_CO_3_, DABCO, and Et_3_N resulted in lower yields than K_2_CO_3_ (Table S2).

Under the optimized reaction conditions, the Suzuki reaction of aryl iodides, bromides and chlorides with various arylboronic acids was investigated (Table 2). The results show that activated or deactivated aryl iodides reactions with phenylboronic acids proceeded in shorter times than aryl bromides and chlorides and gave products in excellent yields (Table 2, entries 1–6). Reactions of less reactive aryl bromides having electron donating groups such as –OMe, –OH, and reaction of more active aryl bromides having electron withdrawing groups such as –CN, –NO_2_, –C=O, –F as well as 2-bromo-1-methyl-4-nitrobenzene with phenylboronic acid proceeded very well and the desired coupling products were achieved in excellent yields (Table 2, entries 7–14). It should be noted that for aryl bromides with electron-donating groups little prolongation of the reaction time was required. The reaction of 4-bromo-1,1′-biphenyl, 1-bromonaphthalene, and 1-bromo-2-methylnaphthalene were performed very well and products were obtained in 90–96% (Table 2, entries 15–17). Reactions of 2-iodothiophene and 5-bromopyrimidine as heterocyclic aryl halides with phenylboronic acid gave products in quantitative yields (Table 2, entries 5 and 18). Reactions of (3-fluorophenyl)boronic acid and 4-methoxyphenylboronic acid as well as PhBF_3_K with aryl bromides afforded very good to excellent yields (Table 2, entries 19–22). Finally, catalyst showed high activity for the reaction of less active aryl chlorides in which desired coupling products were achieved in longer reaction compared to aryl bromides and iodides at 70 °C (Table 2, entries 23–25). Comparing reactivity of 1-iodo-4-nitrobenzene, 1-bromo-4-nitrobenzene, and 1-chloro-4-nitrobenzene with phenylboronic acid, confirmed the halide reactivity order of I > Br > Cl (Table 2, entries 6, 10, and 23).

**Table 2:** The Suzuki–Miyaura cross-coupling reaction of aryl iodides and bromides with different arylboronic acids using the clinochlore-CQDs@Pd catalyst^a^

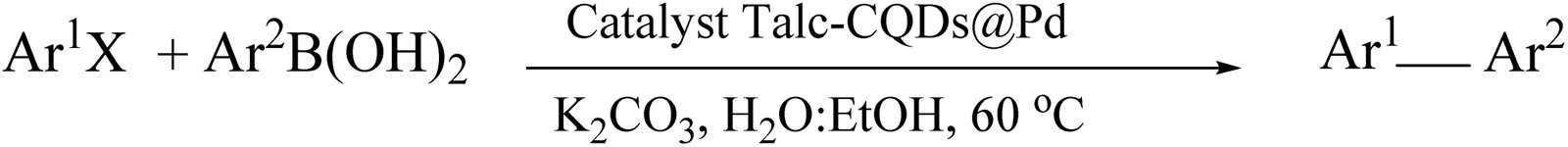					
Entry	Ar^1^X	Ar^2^B(OH)_2_	*t* (h)	Product	Yield^b^ [%]
1	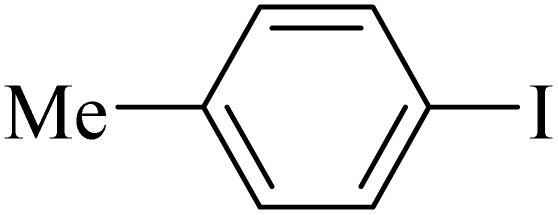	PhB(OH)_2_	0.5	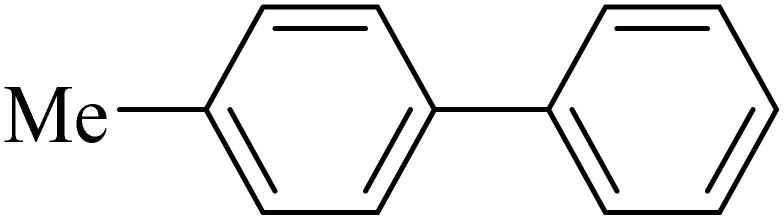	98>
2	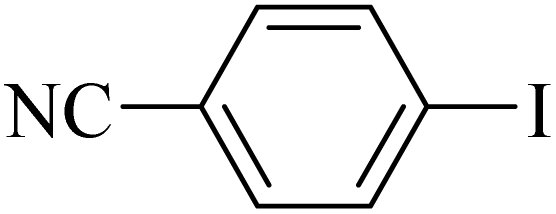	PhB(OH)_2_	0.5	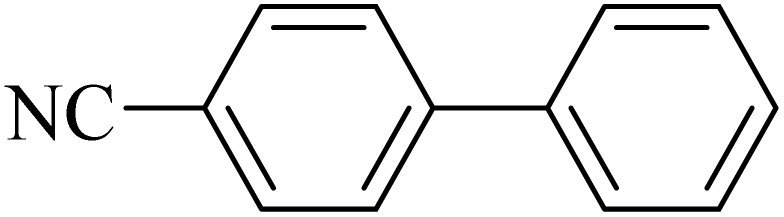	99>
3	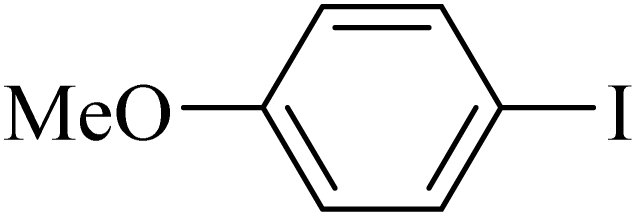	PhB(OH)_2_	1	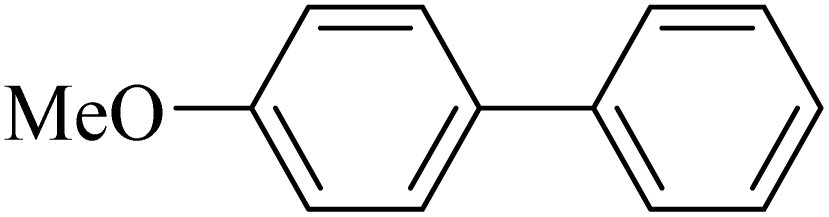	100
4	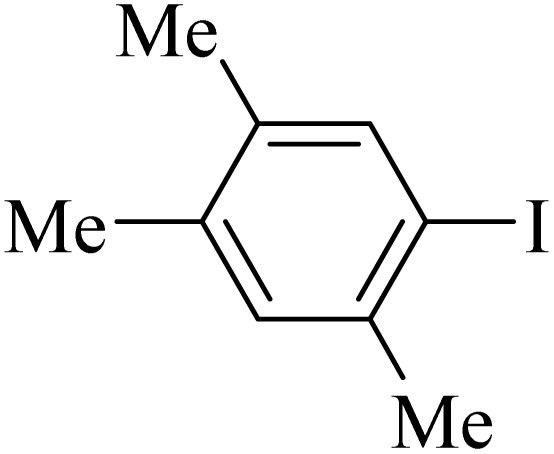	PhB(OH)_2_	1.5	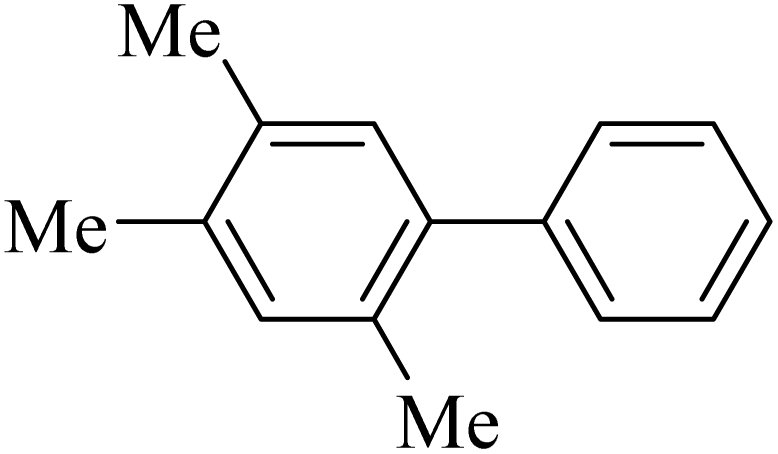	83
5	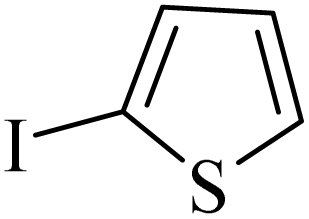	PhB(OH)_2_	1	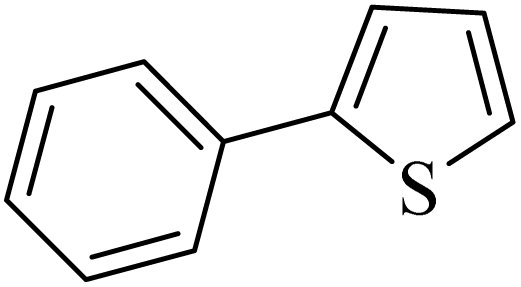	100
6	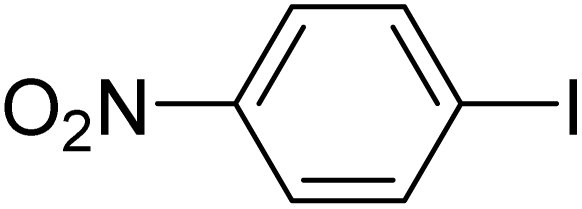	PhB(OH)_2_	0.16	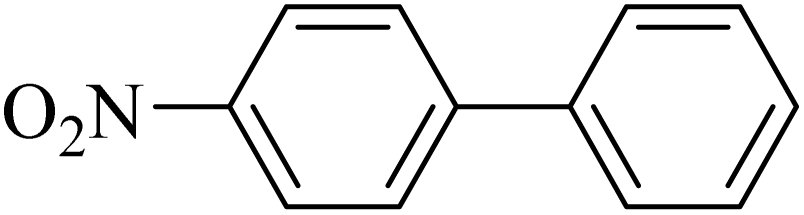	100
7	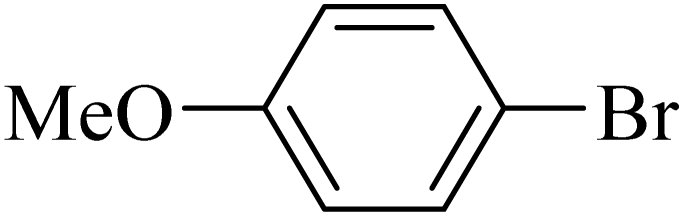	PhB(OH)_2_	6	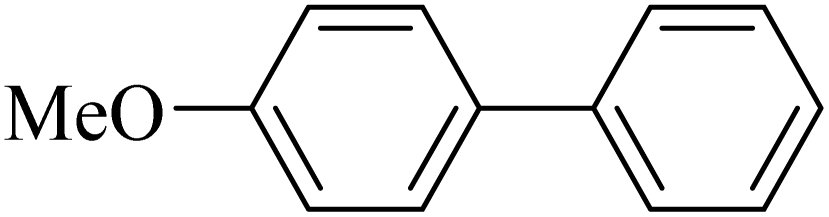	96
8	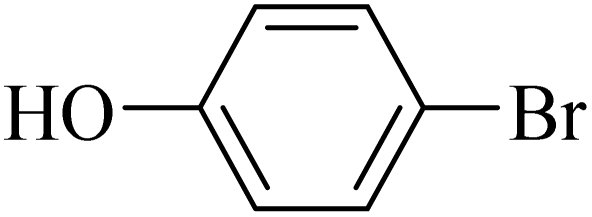	PhB(OH)_2_	1.45	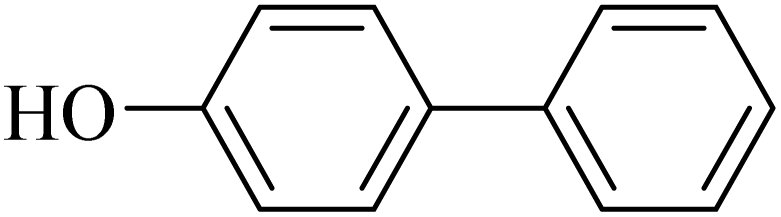	98
9	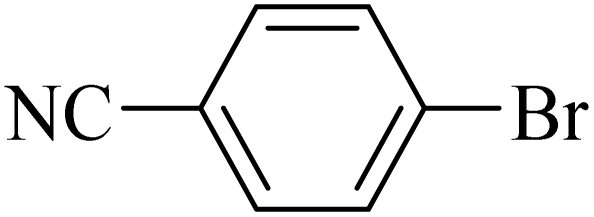	PhB(OH)_2_	2	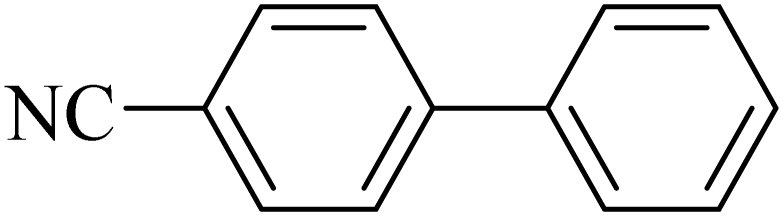	93
10	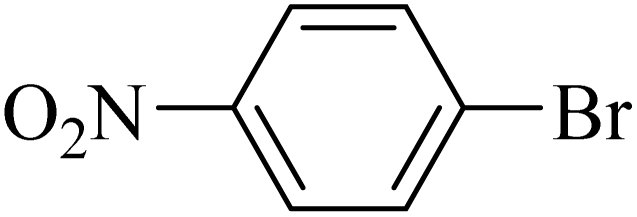	PhB(OH)_2_	1.5	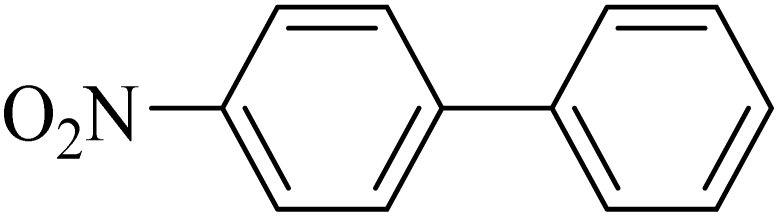	100
11	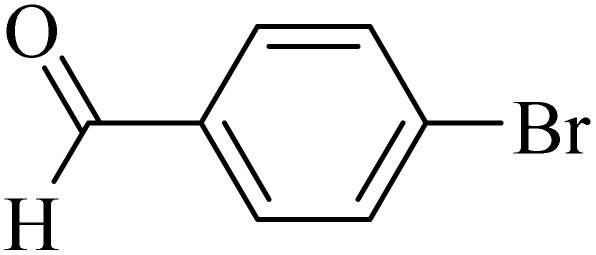	PhB(OH)_2_	1.5	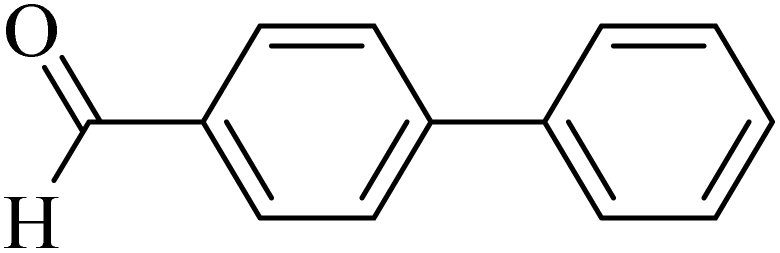	95
12	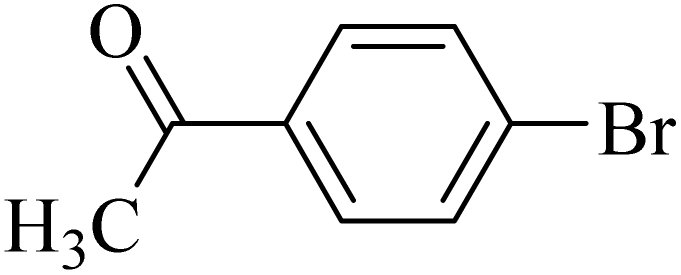	PhB(OH)_2_	1.5	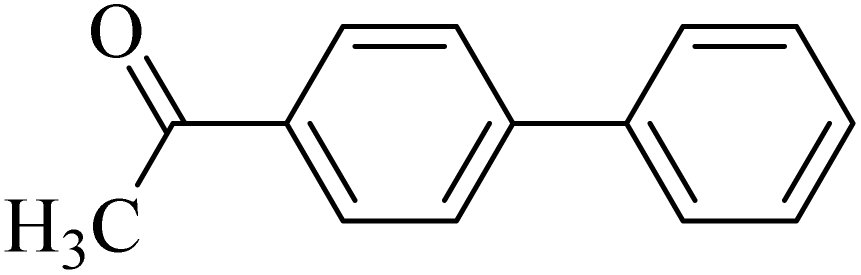	96
13	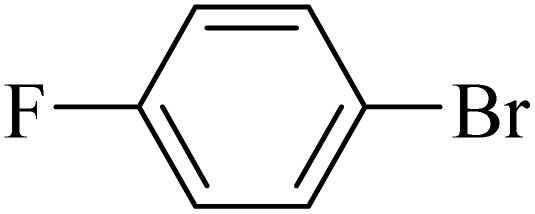	PhB(OH)_2_	2	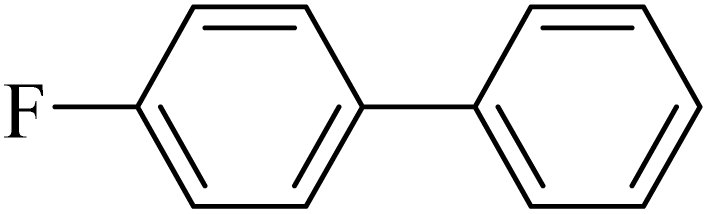	100
14	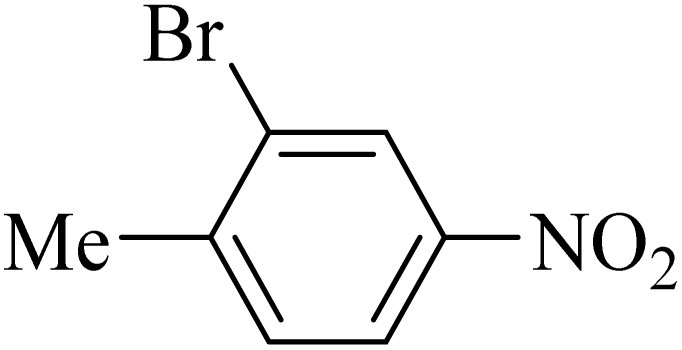	PhB(OH)_2_	3	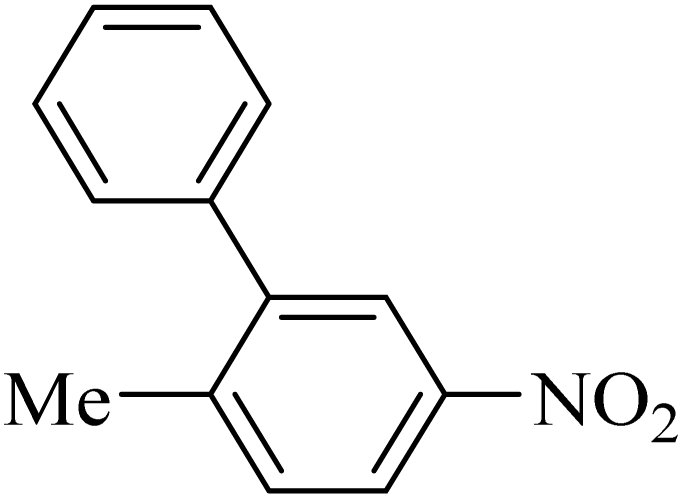	95
15	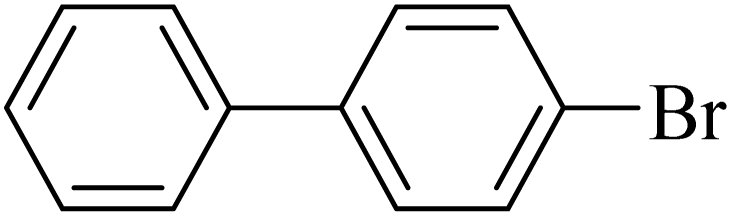	PhB(OH)_2_	1	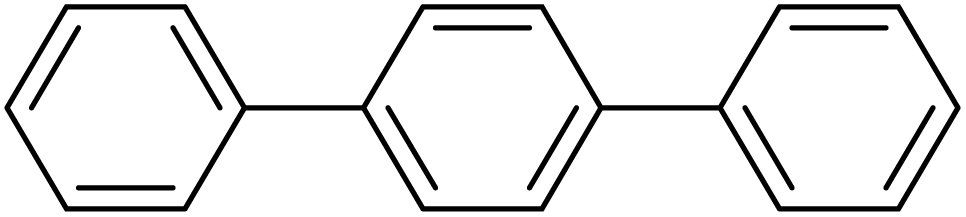	96
16	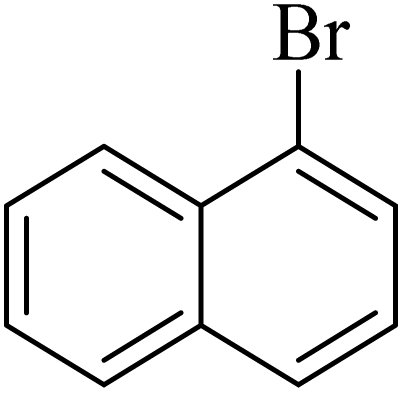	PhB(OH)_2_	1.5	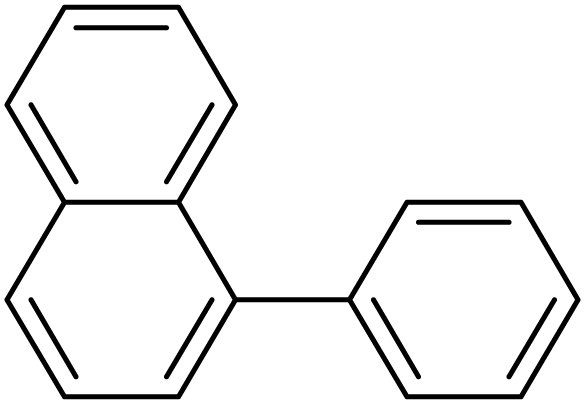	90
17	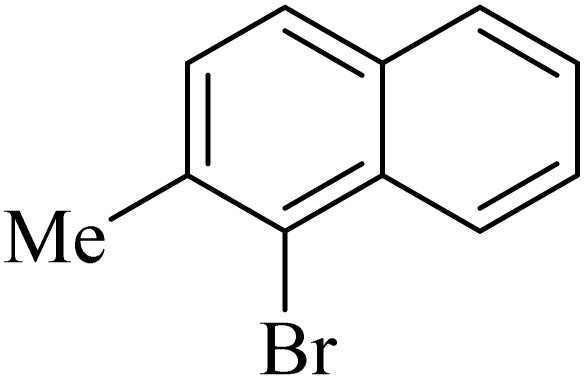	PhB(OH)_2_	4.5	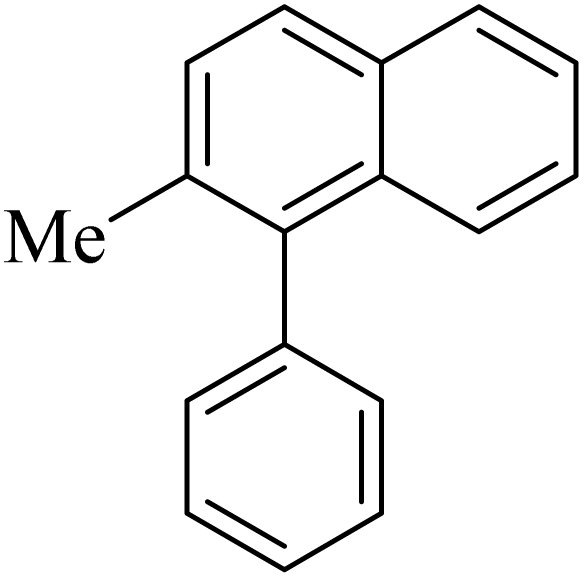	93
18	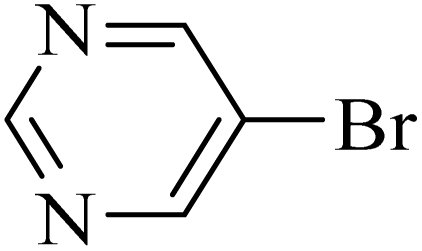	PhB(OH)_2_	1	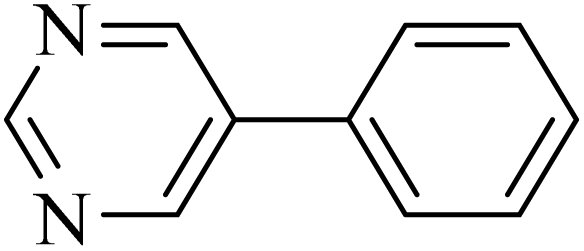	100
19	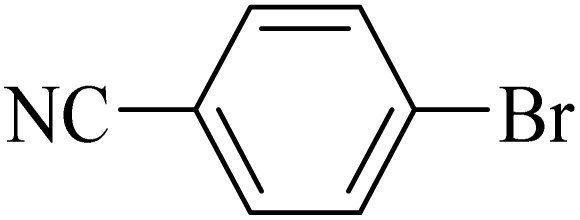	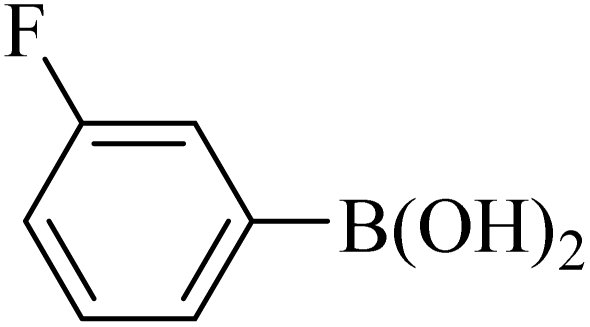	1.5	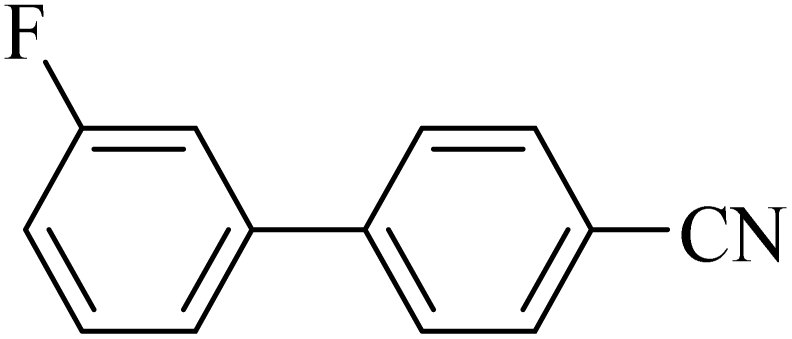	95
20	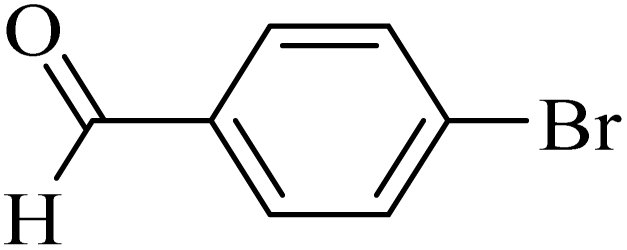	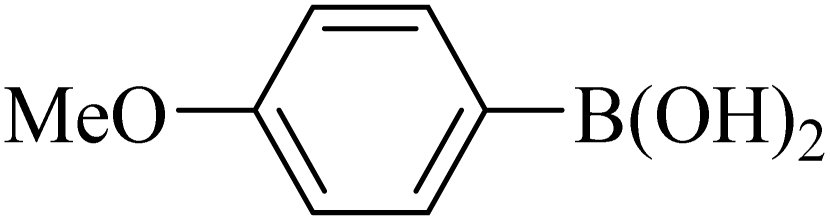	1	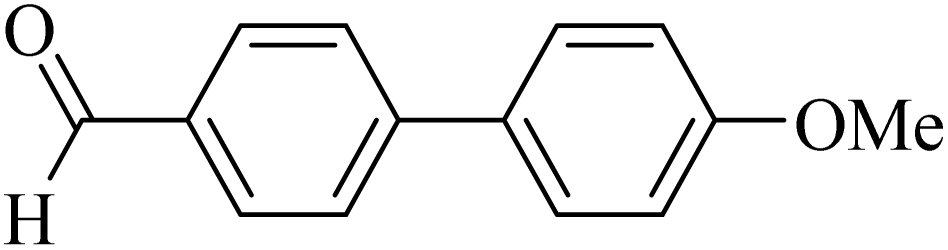	92
21	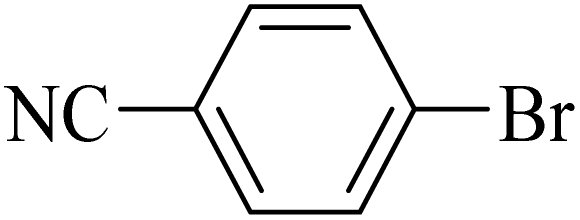	PhBF_3_K	2.5	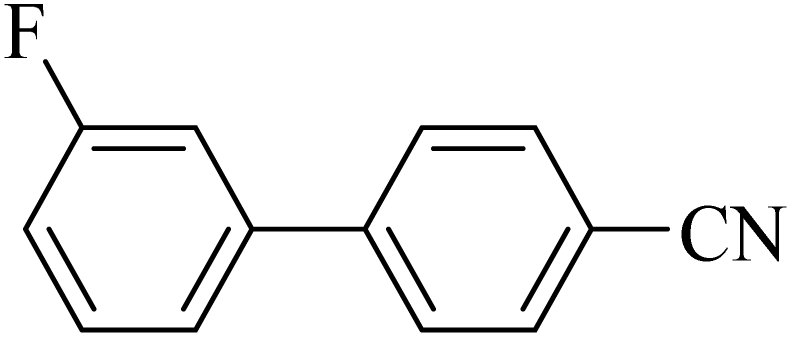	90
22	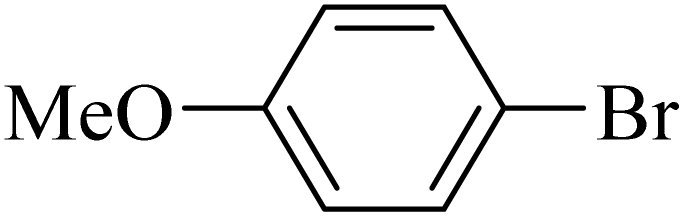	PhBF_3_K	6	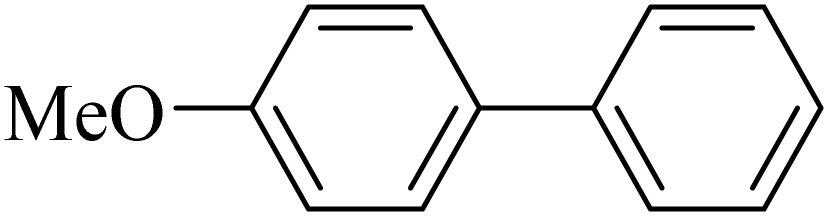	88
23	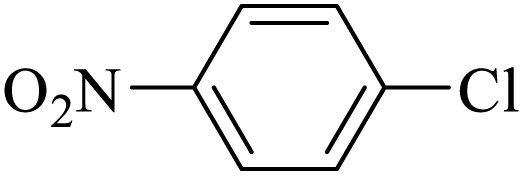	PhB(OH)_2_	4	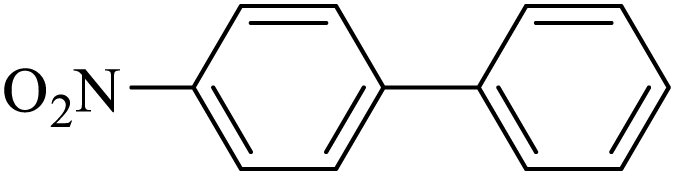	89^c^
24	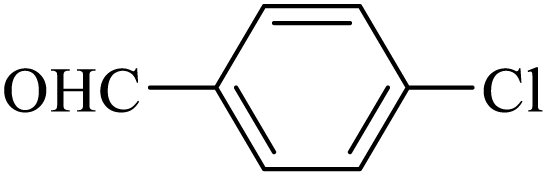	PhB(OH)_2_	4.5	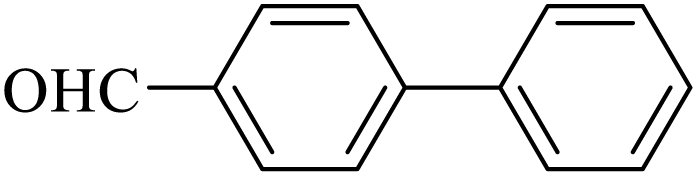	92^c^
25	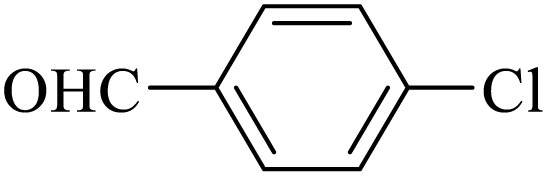	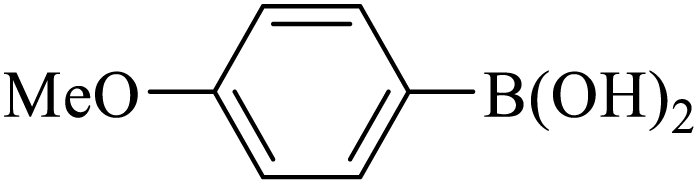	4.5	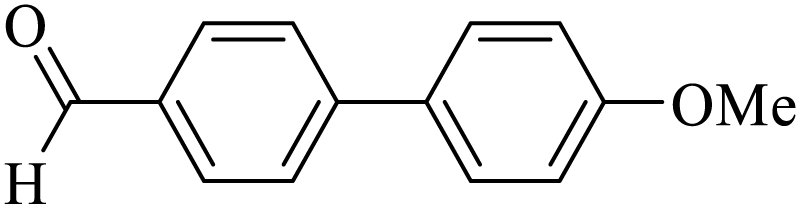	93^c^

a Reaction conditions: Ar^1^X (0.2 mmol), Ar^2^BR (0.3 mmol), solvent (water : ethanol, 1 : 1, 2 mL), K_2_CO_3_ (0.3 mmol), catalyst (0.1 mol% palladium), 60 °C.

b Yields determined by GC.

c Reactions were performed at 70 °C.

**Fig. 7:**
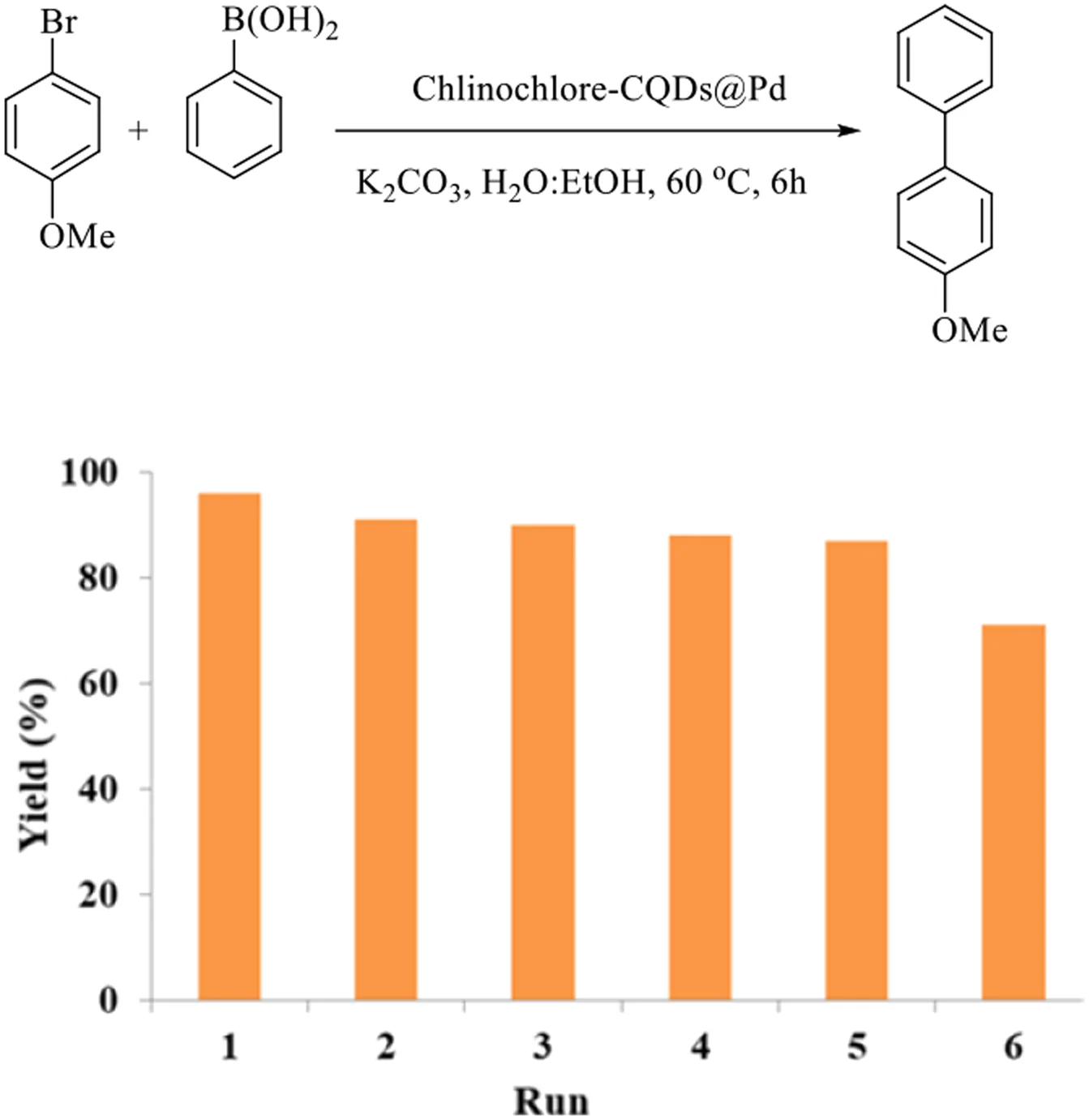
Recycling performance of clinochlore-CQDs@Pd in the Suzuki reaction of 4-bromoanisole with phenylboronic acid.

We studied recyclability of the catalyst in the reaction of 4-bromoanizol with phenylboronic acid under optimized reaction condition. For this purpose, after completing the reaction, catalyst was separated by centrifugation and after washing with ethyl acetate was reused in another run of reaction. Results showed that catalyst is able to recover and reuse for 5 runs with small decrease in activity while in run 6 yield of reaction was decreased to 71% yield (Fig. 7).

**Fig. 8:**
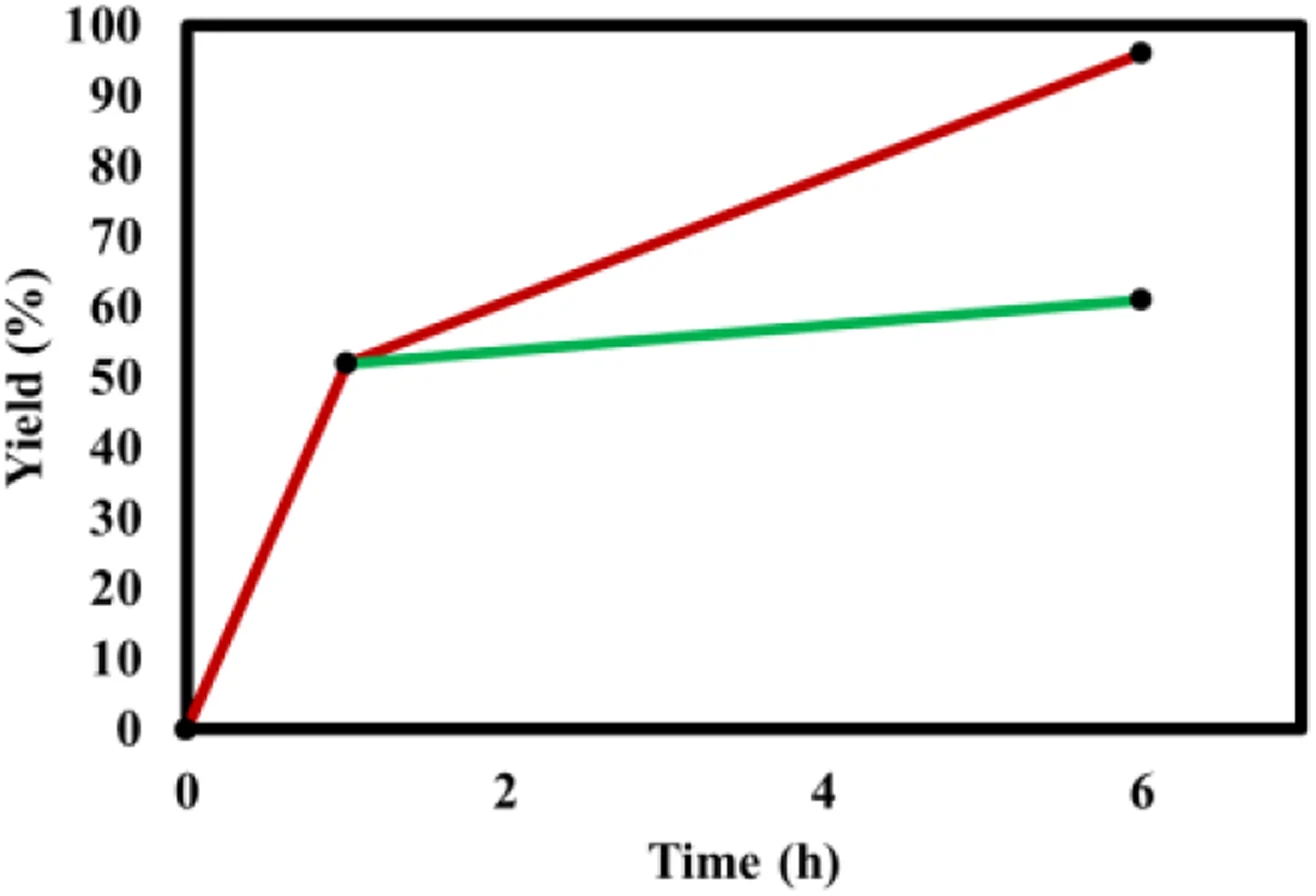
Hot filtration test for the reaction 4-bromoanisole with phenylboronic acid, red line) standard reaction without filtration, green line) hot-filtration after 1 h.

To check the possible leaching of active Pd species and find information about homogeneous or heterogeneous nature of this catalyst, hot filtration test was performed (Fig. 8) For this purpose, two identical reactions between 4-bromoanisole and phenylboronic acid were carried out under the same reaction conditions. After 1 h, first reaction was stopped and yield of reaction was determined by GC to be 52%. In the second experiment, after 1 h of reaction, the catalyst was removed by hot filtration, and the filtrate was allowed to react for an additional 5 h under identical conditions. The reaction afforded a 61% yield, which is considerably lower than that obtained under the standard reaction conditions (96%). This result indicates that only a negligible amount of Pd was leached into the solution, and that the catalytic process proceeds predominantly under heterogeneous conditions.^46^

**Fig. 9:**
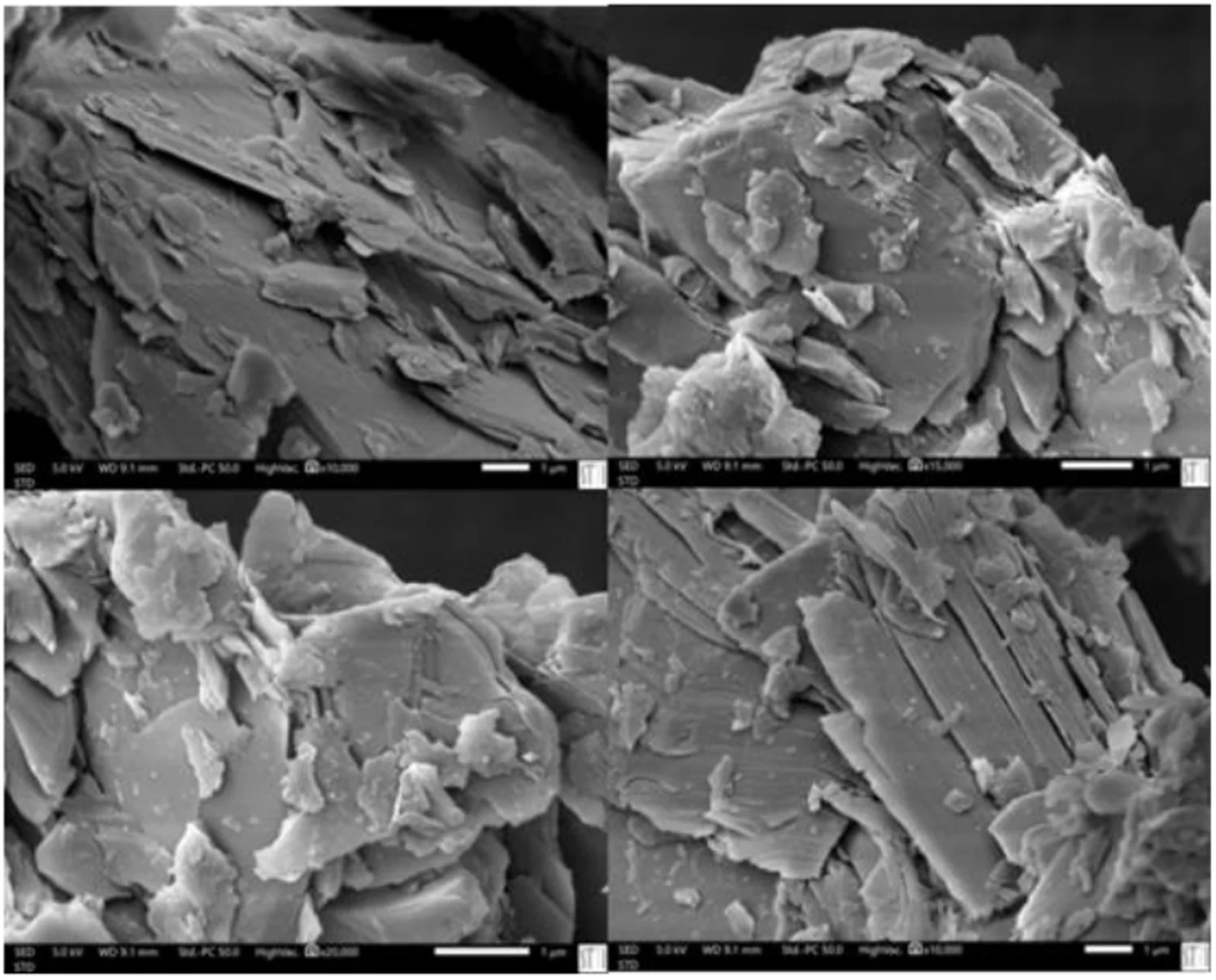
SEM images of reused clinochlore-CQDs@Pd catalyst.

**Fig. 10:**
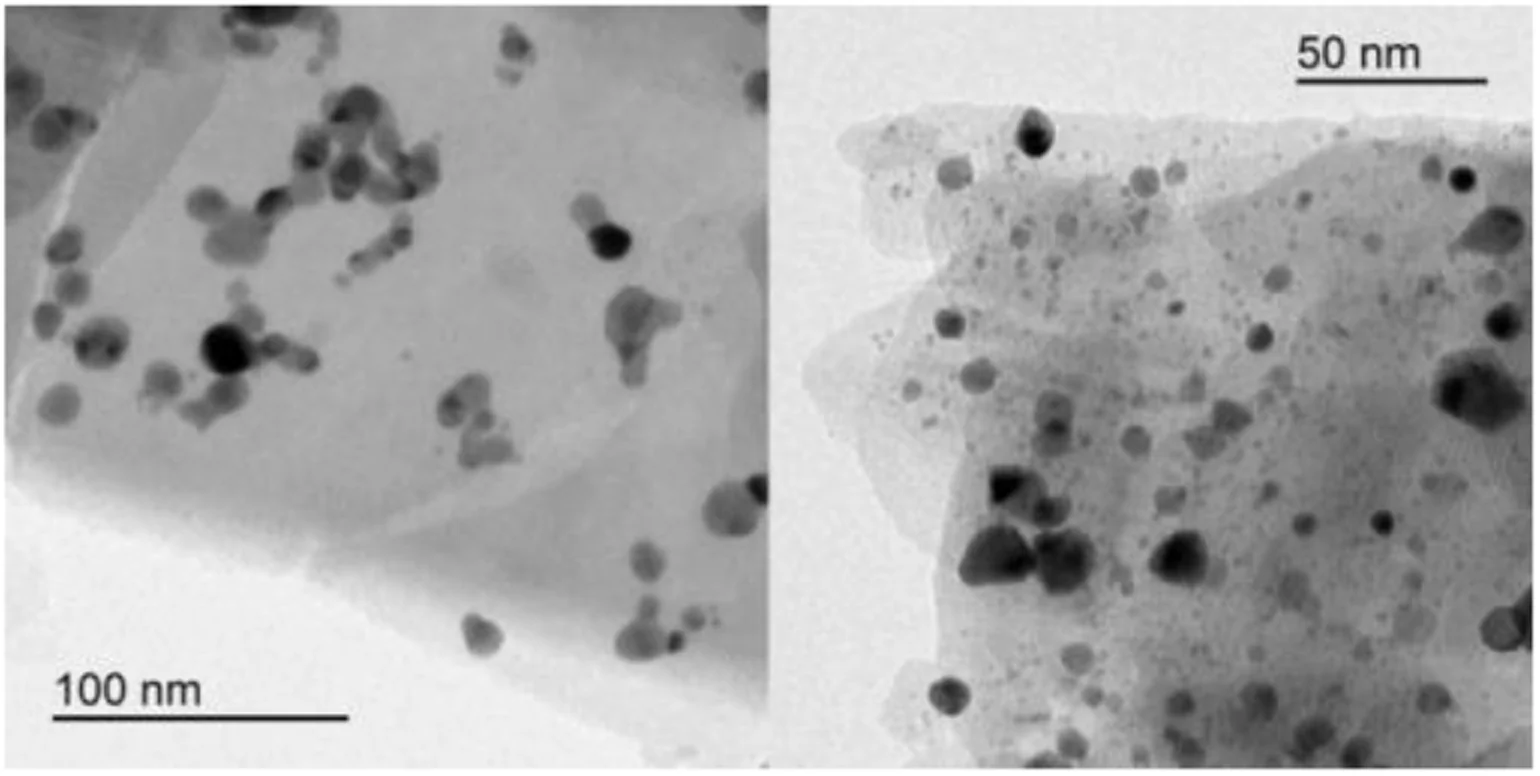
TEM images of reused catalyst.

The morphology of the reused catalyst after six consecutive runs was investigated by SEM and TEM (Fig. 9 and 10). Both analyses showed that the catalyst mainly preserved its original structure, while slight aggregation of Pd nanoparticles was observed in the TEM images.

SEM-Mapping and EDX of reused catalyst showed presence of main elements and preserve of catalyst structure (Fig. S3 and S4). Also, XRD of reused catalyst showed very similar pattern to the fresh catalyst (Fig. S5).

Comparison of the catalytic activity of the clinochlore-CQDs@Pd catalyst with some of the previously reported catalysts in the Suzuki–Miyaura cross-coupling reaction of 4-bromoanisole and phenylboronic acid, used as a common reaction, demonstrated the overall efficiency of the clinochlore-CQDs@Pd catalyst (Table 3).

**Table 3:** The comparative catalytic activity of the clinochlore-CQDs@Pd catalyst with other reported catalysts in the Suzuki–Miyaura cross-coupling reaction

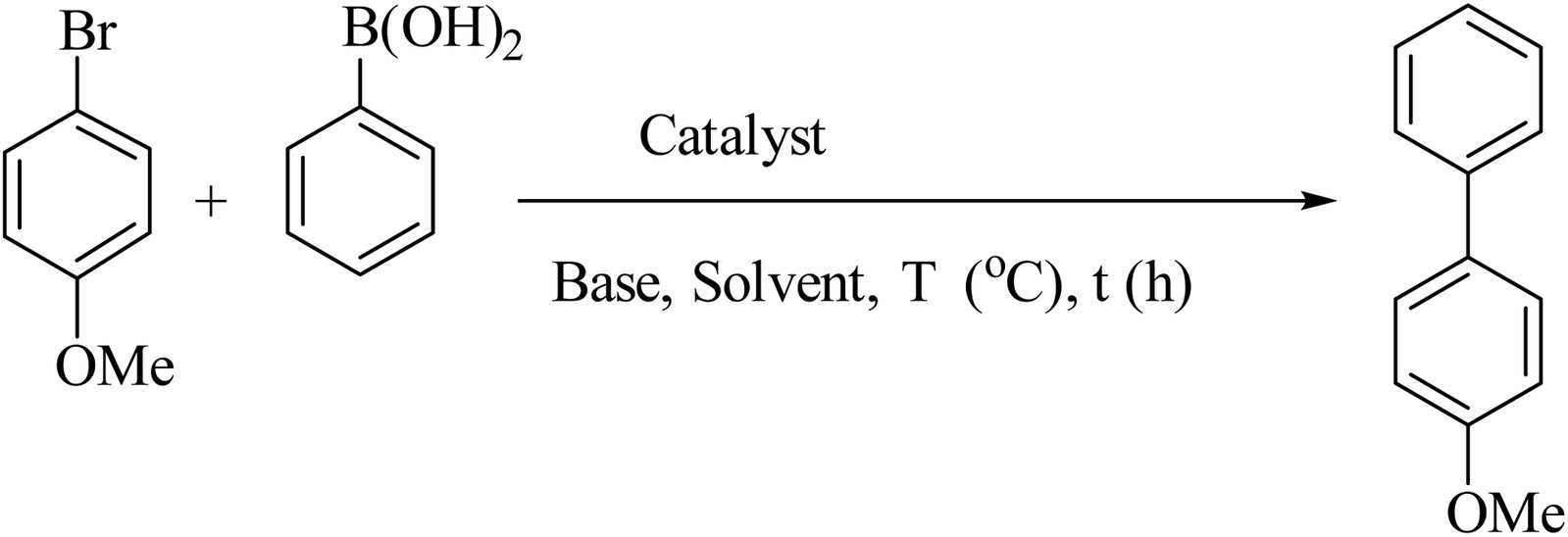								
Entry	Catalyst	Pd (mol%)	Solvent	Base	*T* (°C)	Time (h)	Yield (%)	Reference
1	L_3_PdCl_2_	Pd (1 mol%)	DMF : H_2_O (4 : 1 mL)	K_2_CO_3_	RT	4	60	35
2	GO–TeN–Pd(0)	Pd (0.108 mol%)	DMF : H_2_O (4 : 1 mL)	K_2_CO_3_	100	12	61	36
3	[Pd(L_3_)_2_]	Pd (0.3 mol%)	Methanol (5 mL)	K_2_CO_3_	80	2	67	37
4	Pd(ii)–thiourea	Pd (0.1 mol%)	H_2_O	NaOH	100	12	80	38
5	γ-Fe_2_O_3_-acetamidine-Pd	Pd (0.12 mol%)	DMF (2 mL)	Et_3_N	100	2	81	39
6	Pd(ii)–NHC	Pd (1.0 mol%)	DMA (2 mL)	Cs_2_CO_3_	100	24	86	40
7	γ-Fe_2_O_3_–Pd–NHC-*n*-butyl-SO_3_Na	Pd (0.2 mol%)	H_2_O (6 mL)	Et_3_N	90	13	89	41
8	PdNPs	Pd (2 mol%)	CH_3_OH : CH_3_CN (5 : 5 mL)	K_2_CO_3_	RT	3.5	90	42
9	Diimine/Pd(ii)	Pd(OAc)_2_ (3 mol%)	DMA (2 mL)	K_2_CO_3_	95	5	95	43
10	CoPd	Pd (0.038 mol%)	EtOH : H_2_O (1 : 1 mL)	K_2_CO_3_	60	18	92	44
11	Ni@NH_2_–Pd	Pd (0.011 mol%)	EtOH : H_2_O (1.5 mL)	K_2_CO_3_	80	12	93	45
12	Clinochlore-CQDs@Pd	Pd (0.1 mol%)	EtOH : H_2_O (1 : 1 mL)	K_2_CO_3_	60	6	96	This work

## 3 Experimental section

### 3.1. Materials

All materials were purchased from Sigma-Aldrich, Acros and Merck MilliporeSigma were used without any additional purification. ^1^H NMR and ^13^C NMR spectra were recorded at 400 and 100 MHz, respectively, on a Bruker Avance HD apparatus in CDCl_3_. Reactions were monitored using thin-layer chromatography (TLC) and GC (Varian CP-3800 apparatus). The microstructure and morphology properties of the samples were examined by a FE-SEM (JEOL JSM 840) and TEM (EOL JEM-2010) images. The SEM mapping was measured by Hitachi S3000 N. The product's crystallographic structures were monitored by XRD (Cu Kα radiation, *λ* = 0.154 Å). XPS measurements were characterized using a VGMicrotech Multilab 3000 spectrometer, equipped by Al-Kα spectrometer.

### 3.2. Procedure for preparation of clinochlore-CQDs

A mixture of citric acid (1 g), urea (0.5 g), clinochlore (1 g), and distilled water (25 mL) was sonicated for 5 min, transferred to a Teflon-lined autoclave, and heated at 160 °C for 6 h. Afterward precipitate was separated by centrifugation and washed with water (5 × 5 mL). Then the precipitate was dried in an oven at 60 °C for 24 h.

### 3.3. Procedure for preparation clinochlore-CQDs@Pd

PdCl_2_ (0.055 mmol, 10 mg) was sonicated in water (1 mL) until fully dissolved, then added to a sonicated suspension of clinochlore-CQDs (450 mg), and the resulting mixture was stirred under an argon atmosphere for 24 h. Next, the precipitate was separated by centrifugation, washed with water (5 × 5 mL), and then dried in an oven at 60 °C for 24 h.

### 3.4. General procedure for Suzuki–Miyaura coupling reaction catalyzed by clinochlore-CQDs@Pd

To a 5 mL flask, aryl halide (0.2 mmol), arylboronic acid (0.3 mmol), K_2_CO_3_ (0.3 mmol, 41.5 mg), catalyst (0.1 mol% in Pd), H_2_O (1 mL) and EtOH (1 mL) were added. Reaction was stirred at 60 °C for appropriate reaction time and progresses of reaction were checked by GC and TLC. After completing reactions, ethyl acetate was added and product was extracted. Pure products were obtained after column or plait chromatography.

## 4 Conclusions

In this work, we developed a simple and sustainable recyclable palladium catalyst *via* formation and stabilization of palladium nanoparticles on carbon quantum dots modified clinochlore. The presence of carbon quantum dots played an important and crucial role in formation and uniform dispersion of the palladium nanoparticles on the modified clinochlore surface, while the layered structure of the natural clay was well preserved. The clinochlore-CQDs@Pd showed excellent catalytic activity in the Suzuki–Miyaura cross-coupling reaction of various aryl iodides, bromides and chlorides with arylboronic acids under mild condition in aqueous ethanol under very mild conditions. Combining abundant natural mineral clinochlore with carbon quantum dots provided a green approach for designing robust and recyclable heterogeneous palladium catalysts.

## Author contributions

Material preparation and data collection were performed by Setare Jafarpour. Writing, analysis and edit of manuscript were performed by Mohammad Gholinejad and José Miguel Sansano.

## Conflicts of interest

Authors declare they have no conflicts of interest.

## Supplementary Material

RA-016-D6RA03494B-s001

## Data Availability

The authors affirm that the data supporting the findings of this study are included in the article and supplementary information (SI). Additional data can be made available from the corresponding author upon reasonable request. Supplementary information: EDX of the catalyst, tables of optimizations, absorption–desorption isotherm of the catalyst, mapping images, EDX and XRD of reused catalyst, ^1^H NMR and ^13^C NMR of coupling products. See DOI: https://doi.org/10.1039/d6ra03494b.
