# Supplementary material for: Carbon quantum dot modified clinochlore as a green support for the formation and stabilization of palladium nanoparticles in aqueous Suzuki–Miyaura coupling
Source: RSC Adv. 2026 Jul 24;16(38):42714–23. doi: 10.1039/d6ra03494b (PMC13397079; doi:10.1039/d6ra03494b)
Supplement: Supplementary file 1 [file RA-016-D6RA03494B-s001.pdf]

Supplementary Information (SI) for RSC Advances.
This journal is © The Royal Society of Chemistry 2026

Carbon Quantum Dots Modified Clinochlore as a Green Support for formation and stabilization of Palladium Nanoparticles in Aqueous Suzuki-Miyaura Coupling

Mohammad Gholinejad[a,b,*] Setare Jafarpour[a], José M Sansano[c]

[a] Department of Chemistry, Institute for Advanced Studies in Basic Sciences (IASBS), Zanjan 45137-66731, Iran. E-mail: gholinejad@iasbs.ac.ir

[b] Basic Sciences and Modern Technologies (RBST), Institute for Advanced Studies in Basic Sciences (IASBS), Zanjan 45137-66731, Iran

[c] Departamento de Química Orgánica, Instituto de Síntesis Orgánica, and Centro de Innovación en Química Avanzada (ORFEO-CINQA), Universidad de Alicante, 03690 Alicante, Spain

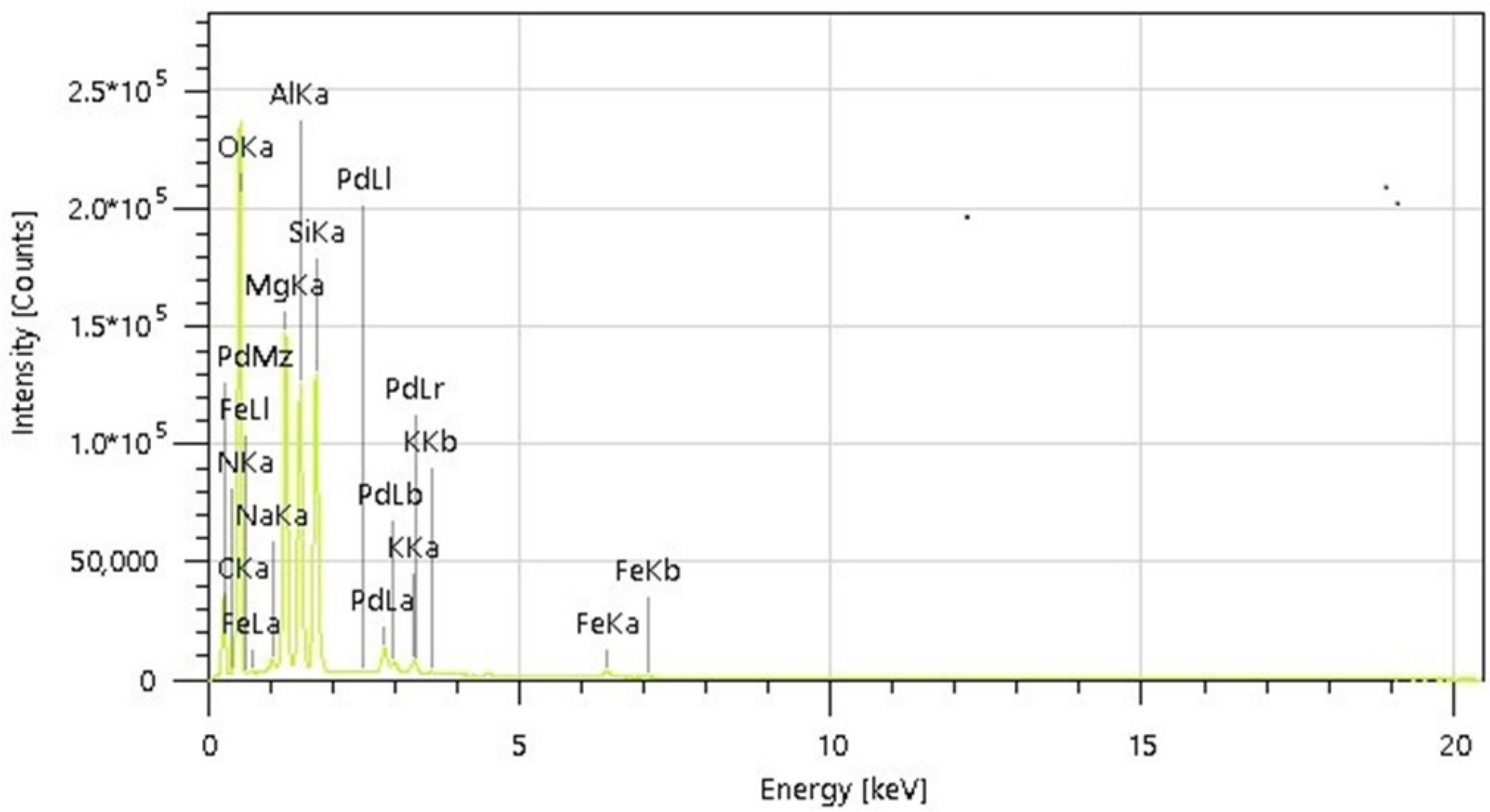

Figure S1. EDX spectrum of the clinochlore-CQDs@Pd

Table S1. Effect of different solvents in the coupling of 4-bromoanisole and phenylboronic acid.

Entry	Solvent	Yield (%)
**1**	**H_2O: EtOH (1:1)**	**96**
2	H_2O: EtOH (2:1)	30
3	H_2O: EtOH (1:2)	70
4	EtOH	50
5	H_2O	7
6	THF	12
7	DMF	8

Table S2. The effect of different bases in the reaction of 4-bromoanisole and phenylboronic acid.

Entry	Base	Yield (%)
**1**	**K_2CO_3**	**96**
2	DABCO	60
3	Et_3N	15
4	Na_2CO_3	75

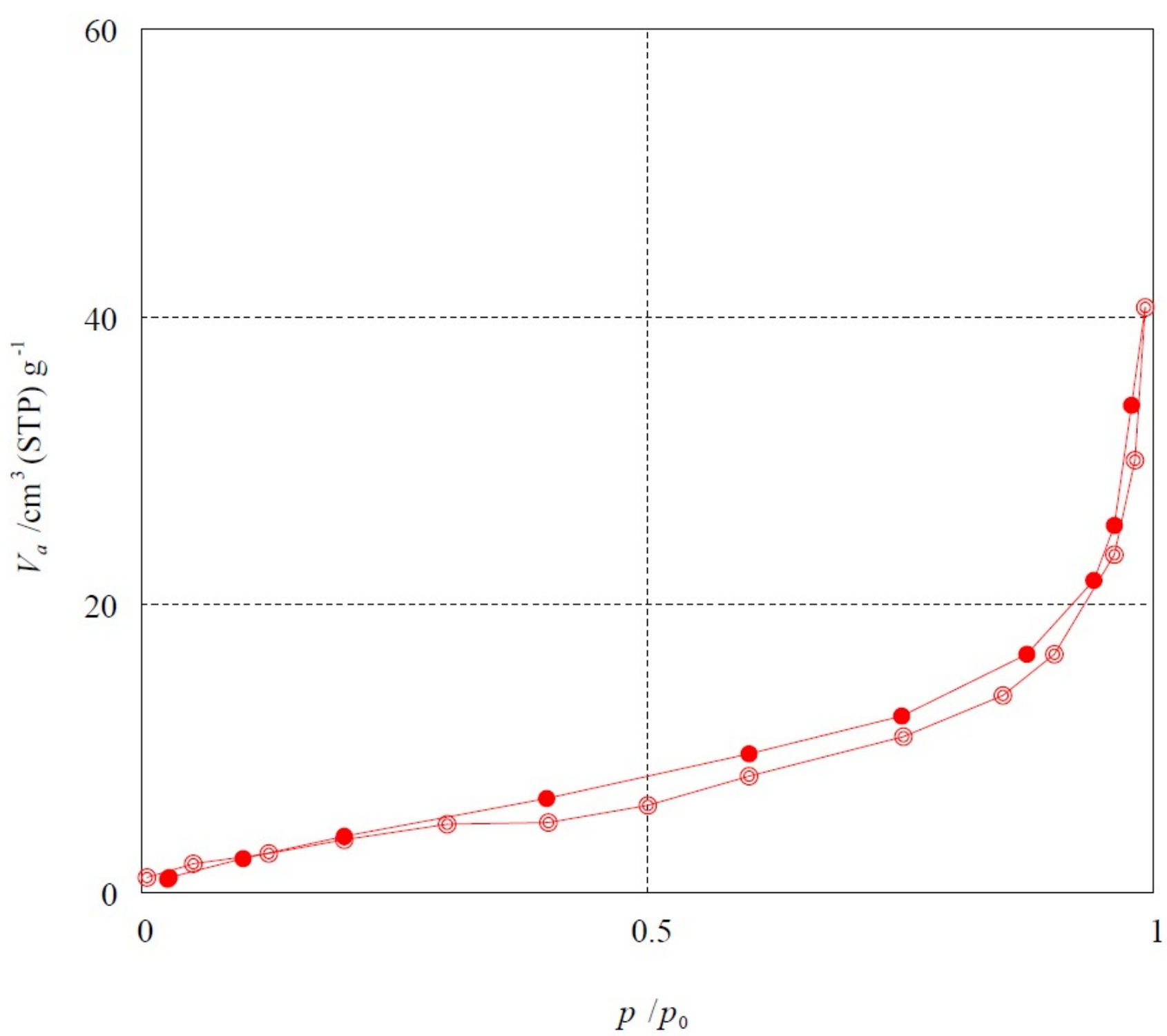

Figure S2. Absorption-desorption isotherm of clinochlore-CQDs@Pd

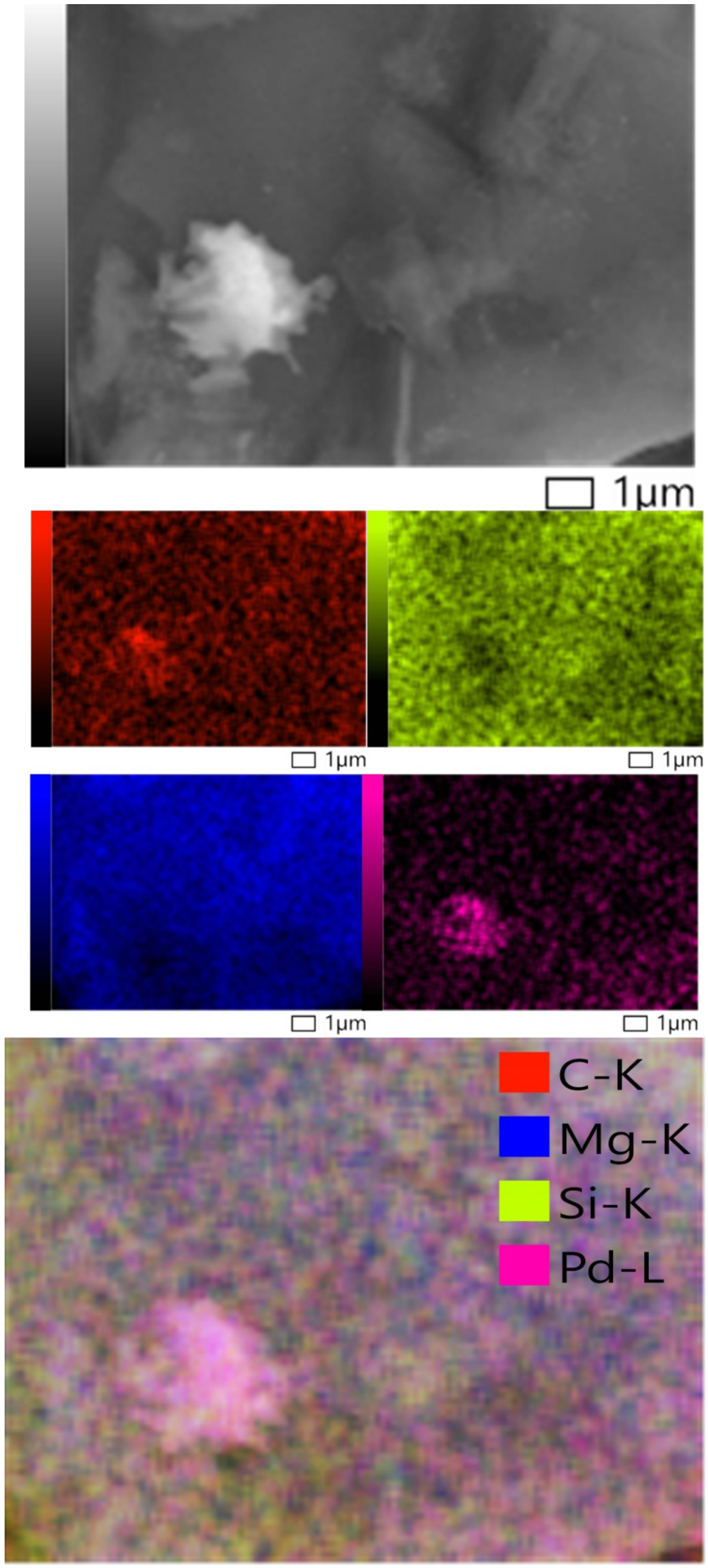

Figure S3. SEM mapping images of the reused clinochlore-CQDs@Pd

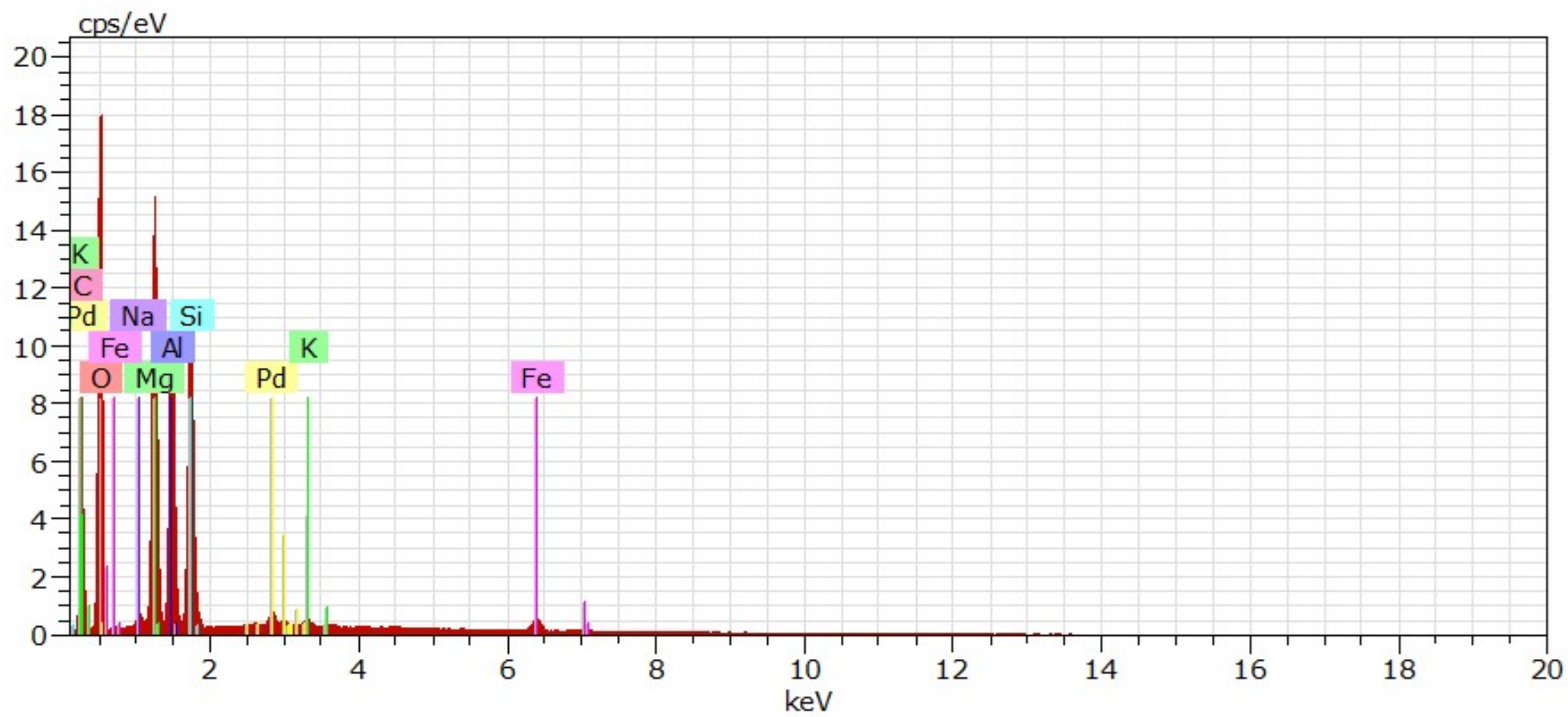

Figure S4. EDX spectrum of the reused clinochlore-CQDs@Pd

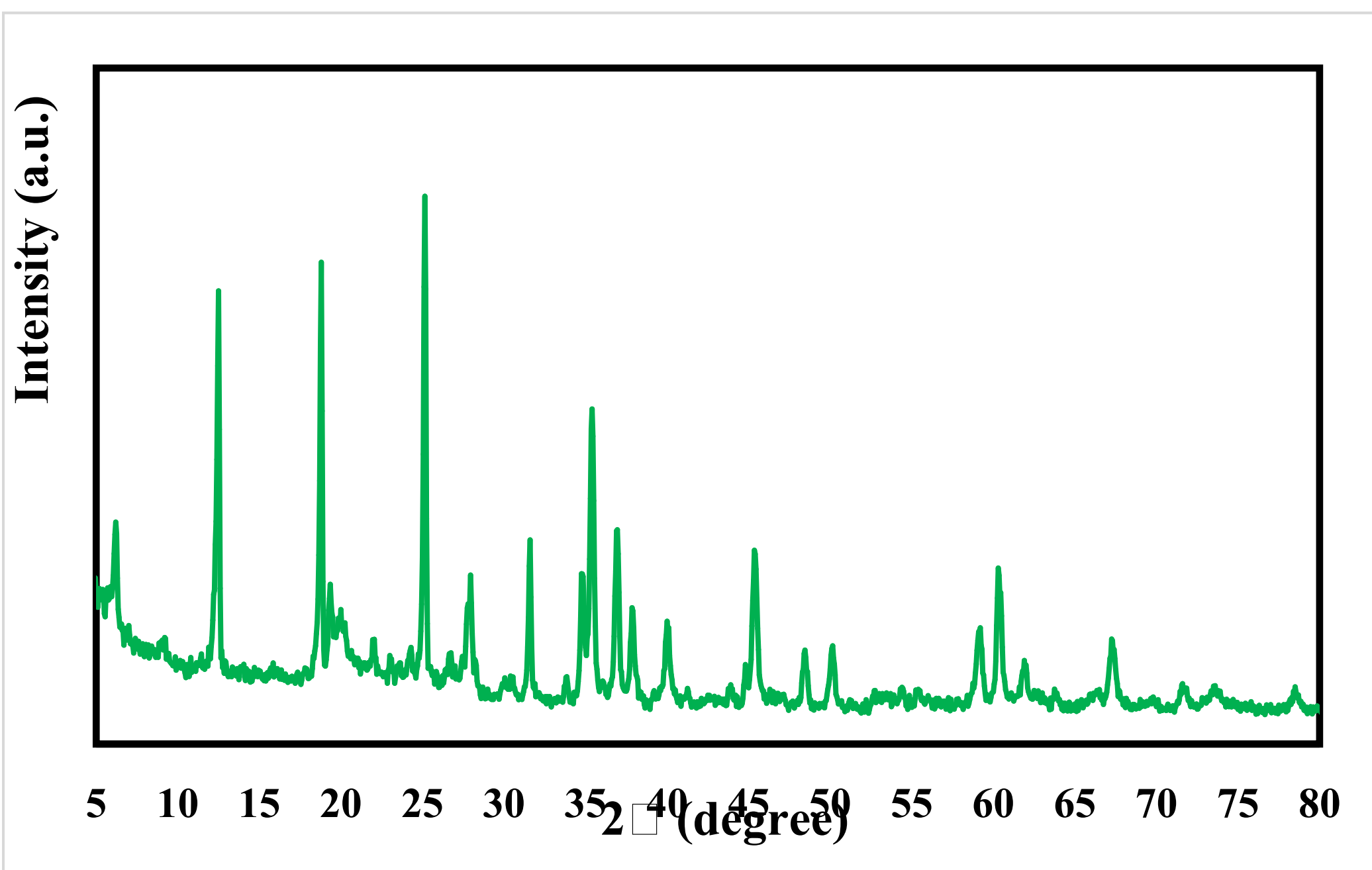

Figure S5. XRD pattern of the reused clinochlore-CQDs@Pd

^{1}H NMR, ^{13}C NMR of coupling products

4-methyl-1,1'-biphenyl [1]

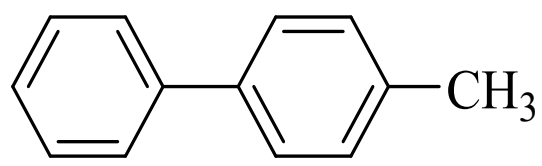

^{1}H NMR (400 MHz, $CDCl_3$) δ (ppm): 7.66 – 7.60 (m, 2H), 7.57 – 7.52 (m, 2H), 7.50 – 7.45 (m, 2H), 7.39 – 7.35 (s, 1H), 7.31 (d, J = 7.9 Hz, 2H), 2.45 (s, 3H). ^{13}C NMR (100 MHz, $CDCl_3$) δ (ppm): 141.2, 138.4, 137.1, 129.5, 128.8, 127.0, 127.0, 21.1.

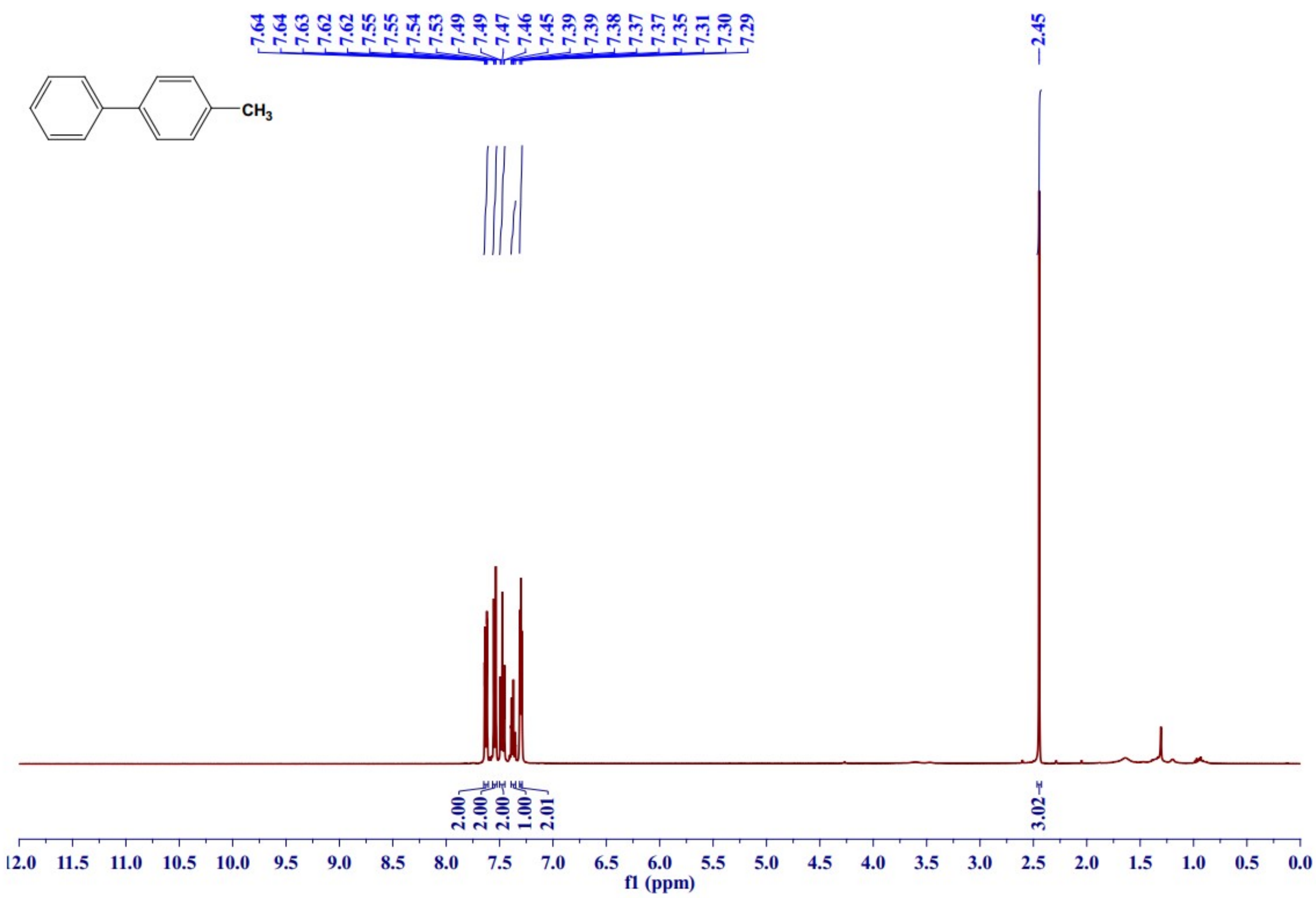

^{1}H NMR of 4-methyl-1,1'-biphenyl

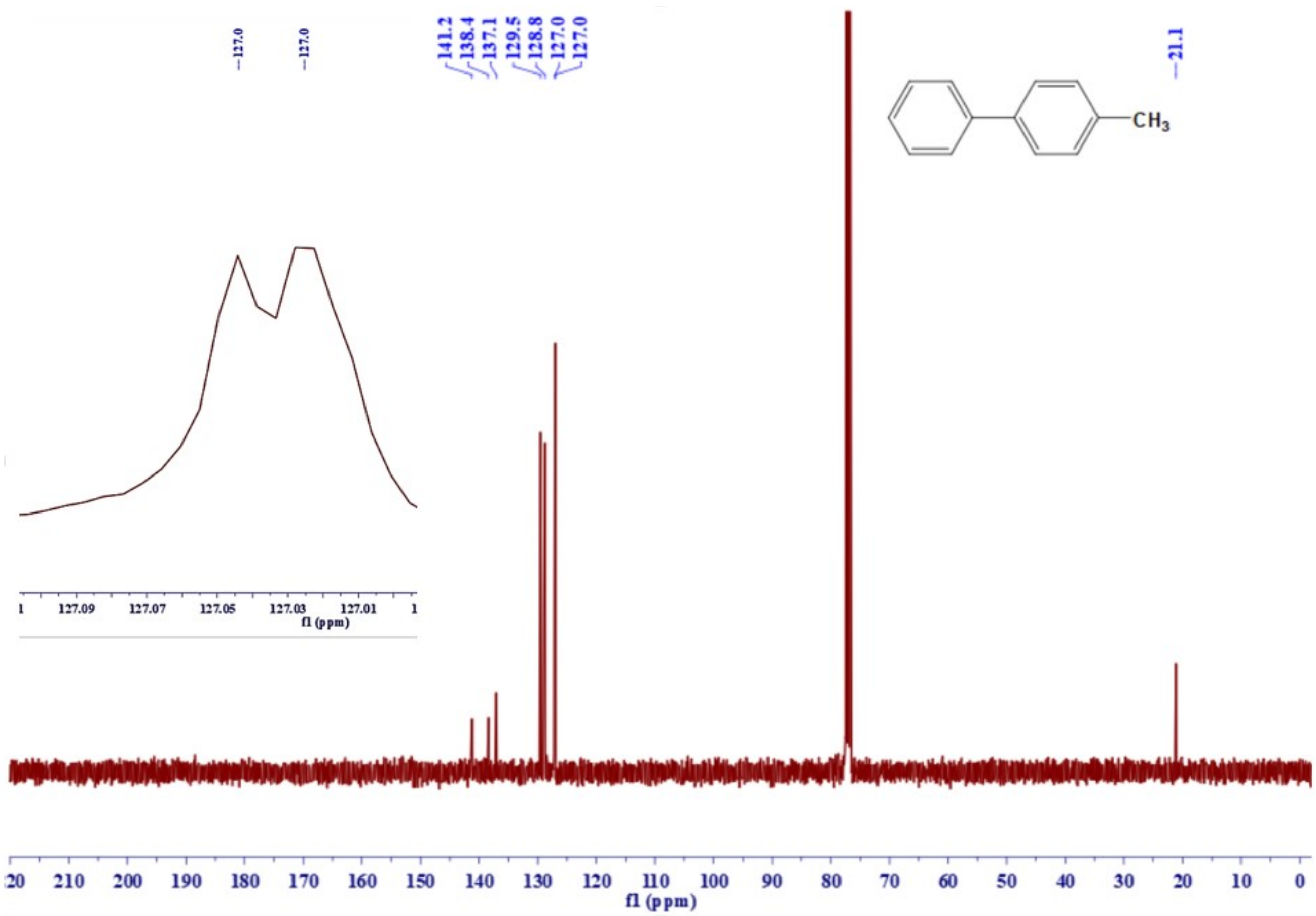

^{13}C NMR of 4-methyl-1,1'-biphenyl

4-methoxy-1,1'-biphenyl [1]

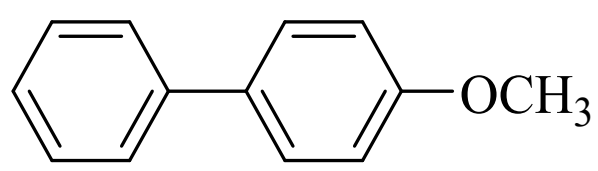

^{1}H NMR (400 MHz, $CDCl_3$) δ 7.68 – 7.56 (m, 4H), 7.53 – 7.44 (m, 2H), 7.37 (d, *J* = 7.4 Hz, 1H), 7.05 (s, 2H), 3.91 (s, 3H). ^{13}C NMR (101 MHz, $CDCl_3$) δ 159.2, 140.9, 133.8, 128.8, 128.2, 126.8, 126.7, 114.2, 55.4.

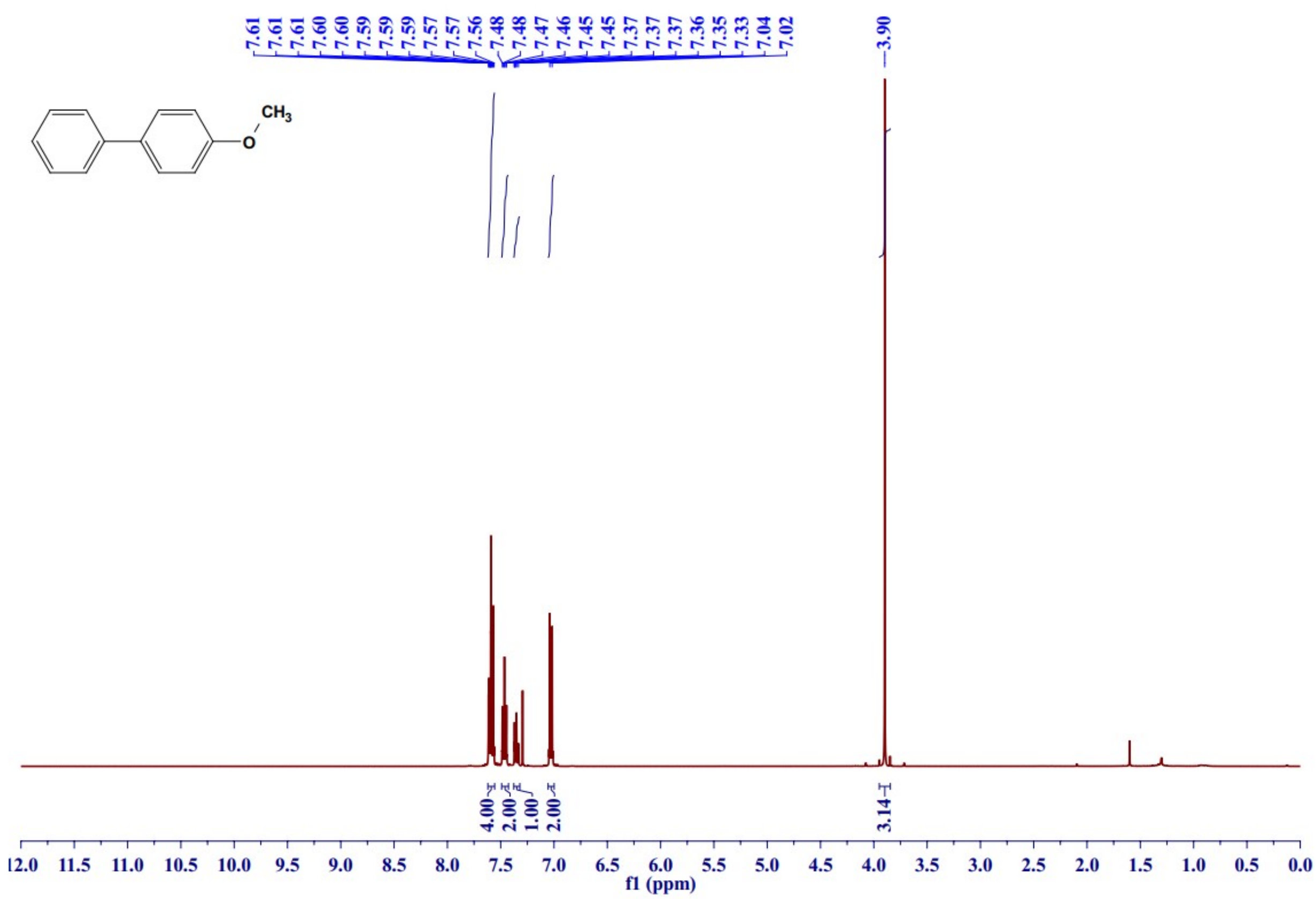

^{1}H NMR of 4-methoxy-1,1'-biphenyl

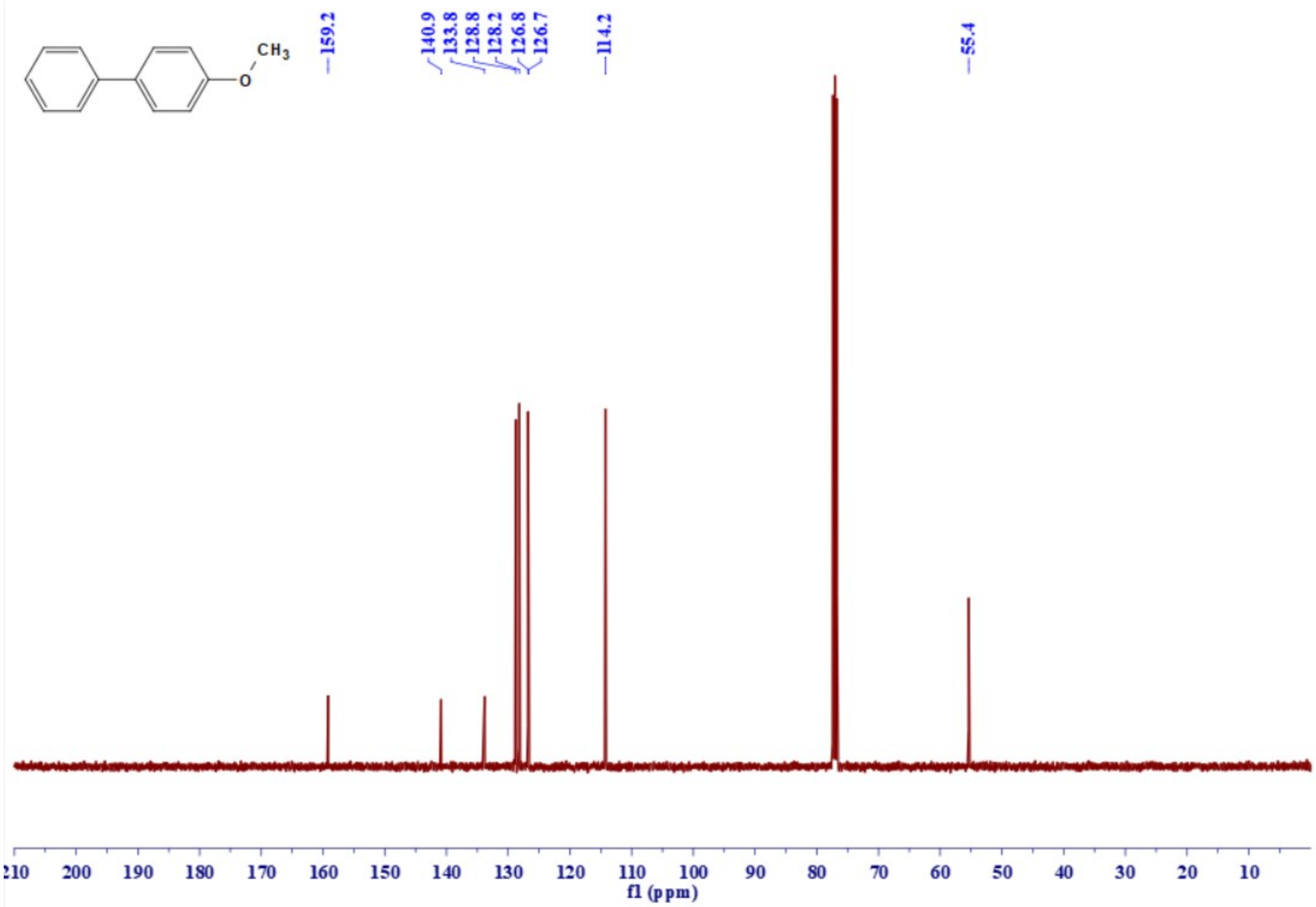

^{13}C NMR of 4-methoxy-1,1'-biphenyl

[1,1'-biphenyl]-4-carbaldehyde [2]

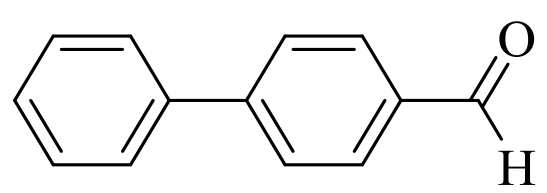

^{1}H NMR (400 MHz, $CDCl_3$) δ (ppm): 10.10 (s, 1H), 8.01 – 7.98 (m, 2H), 7.81 – 7.79 (m, 2H), 7.69 – 7.67 (m, 2H), 7.54 – 7.44 (m, 3H). ^{13}C NMR (100 MHz, $CDCl_3$) δ (ppm): 192.0, 147.2, 139.7, 135.2, 130.3, 129.1, 128.5, 127.7, 127.4.

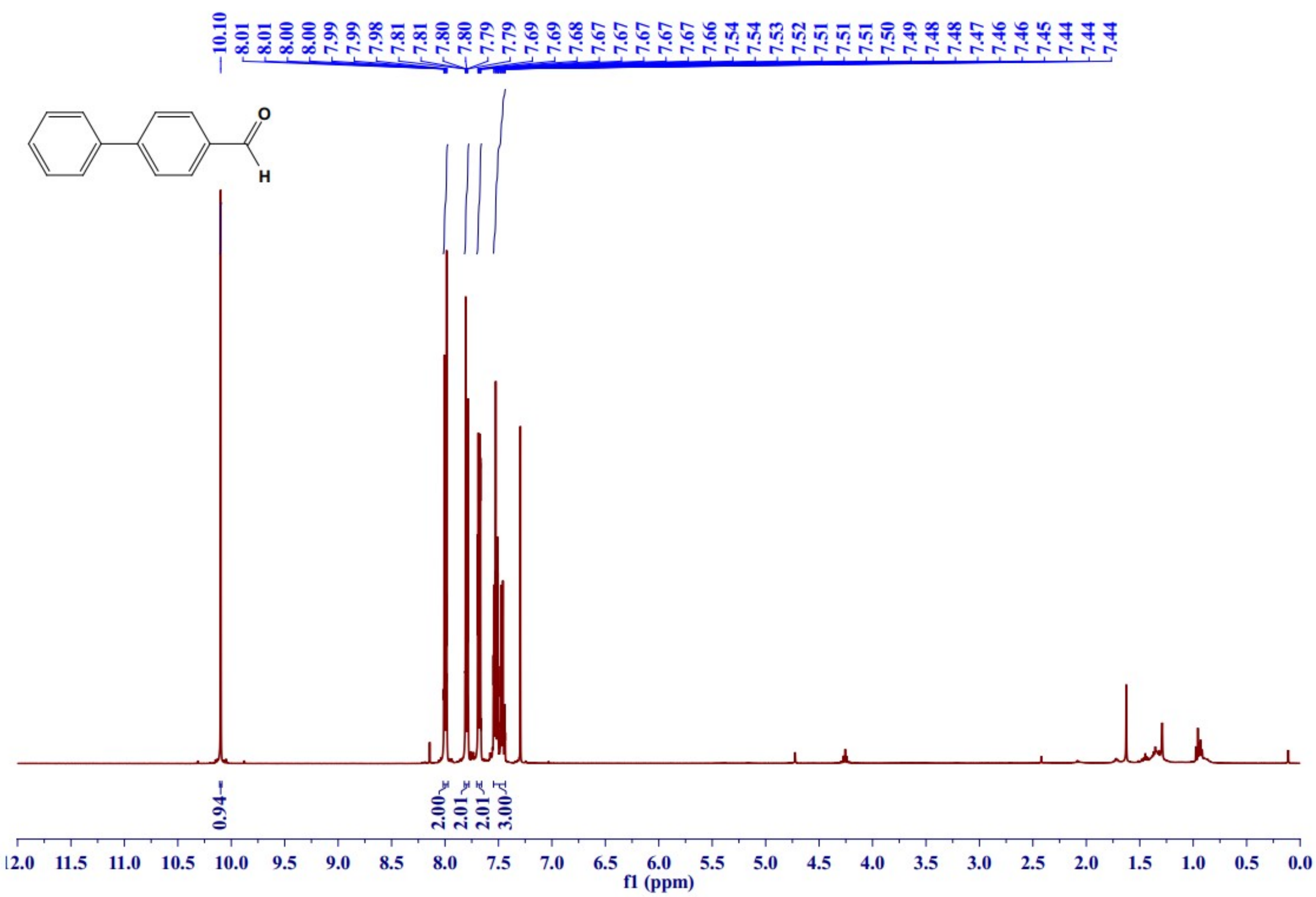

^{1}H NMR of [1,1'-biphenyl]-4-carbaldehyde

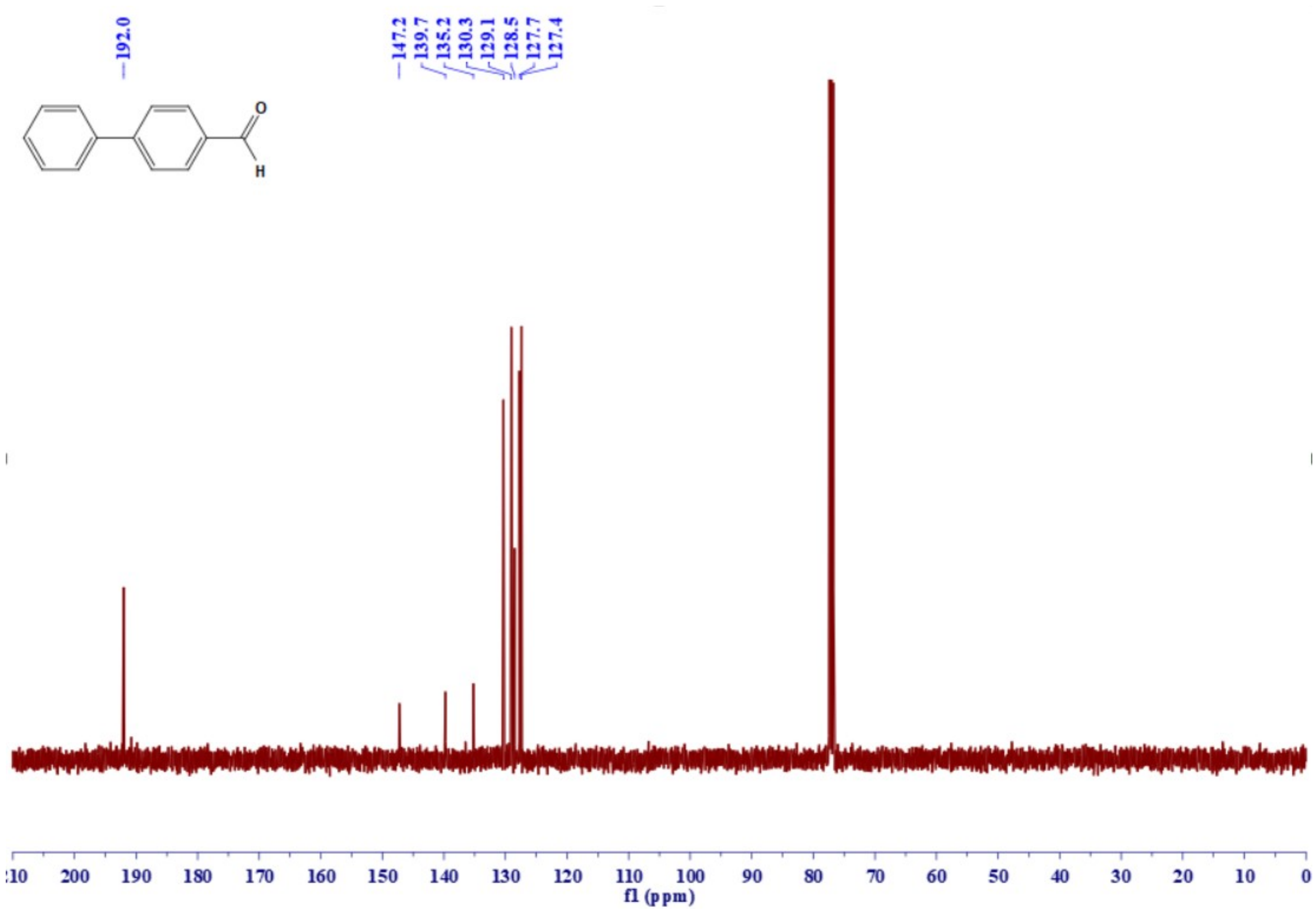

^{13}C NMR of [1,1'-biphenyl]-4-carbaldehyde

[1,1'-biphenyl]-4-ol [10]

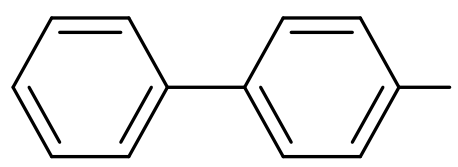

^{1}H NMR (400 MHz, $CDCl_3$) δ (ppm): 7.58 (m, 2H), 7.54 – 7.51 (m, 2H), 7.48 – 7.43 (m, 2H), 7.37 – 7.33 (m, 1H), 6.97 – 6.93 (m, 2H), 4.88 (s, 1H). ^{13}C NMR (100 MHz, $CDCl_3$) δ (ppm): 155.1, 140.8,134.1,128.8,128.4,126.8,115.7.

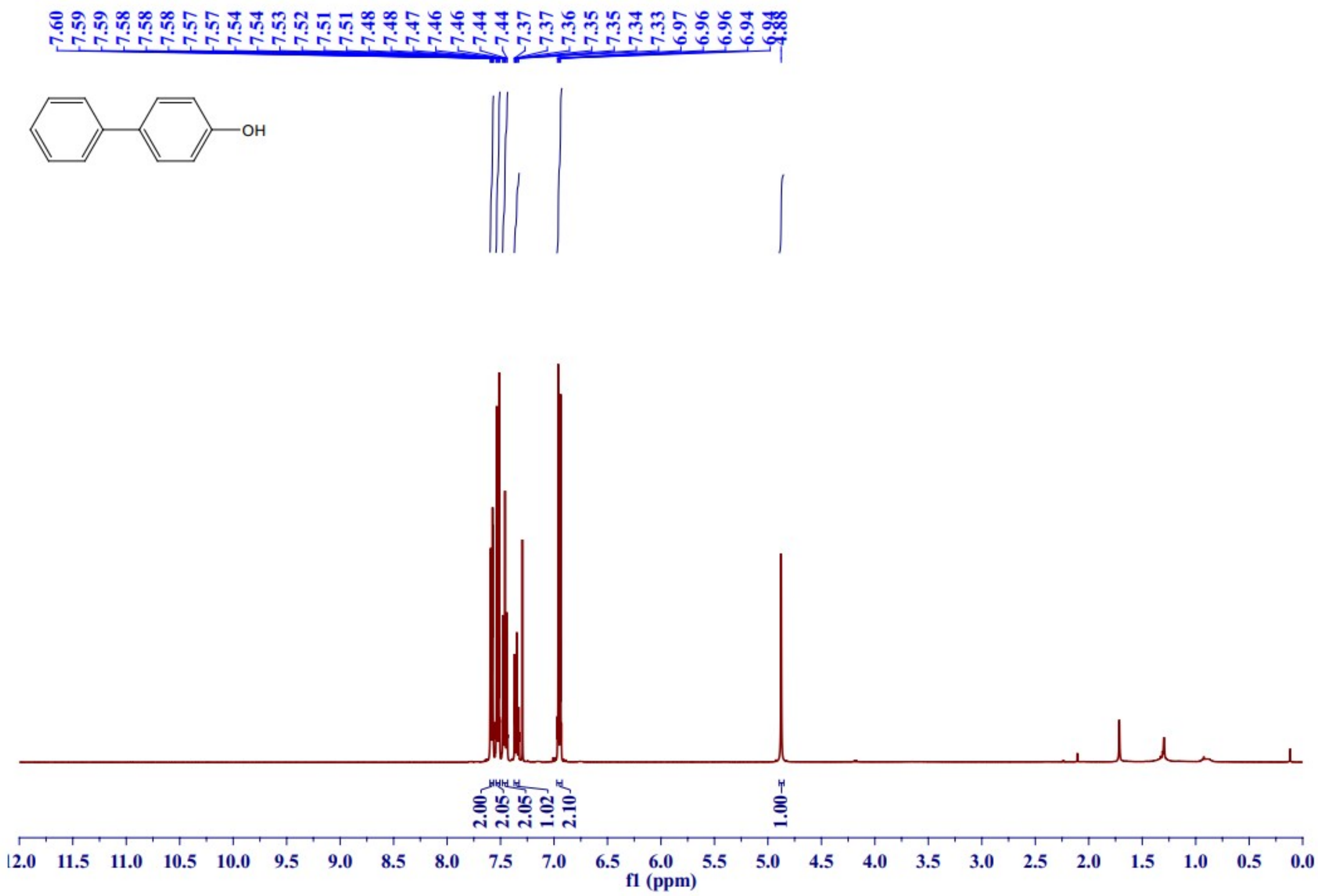

^{1}H NMR of [1,1'-biphenyl]-4-ol

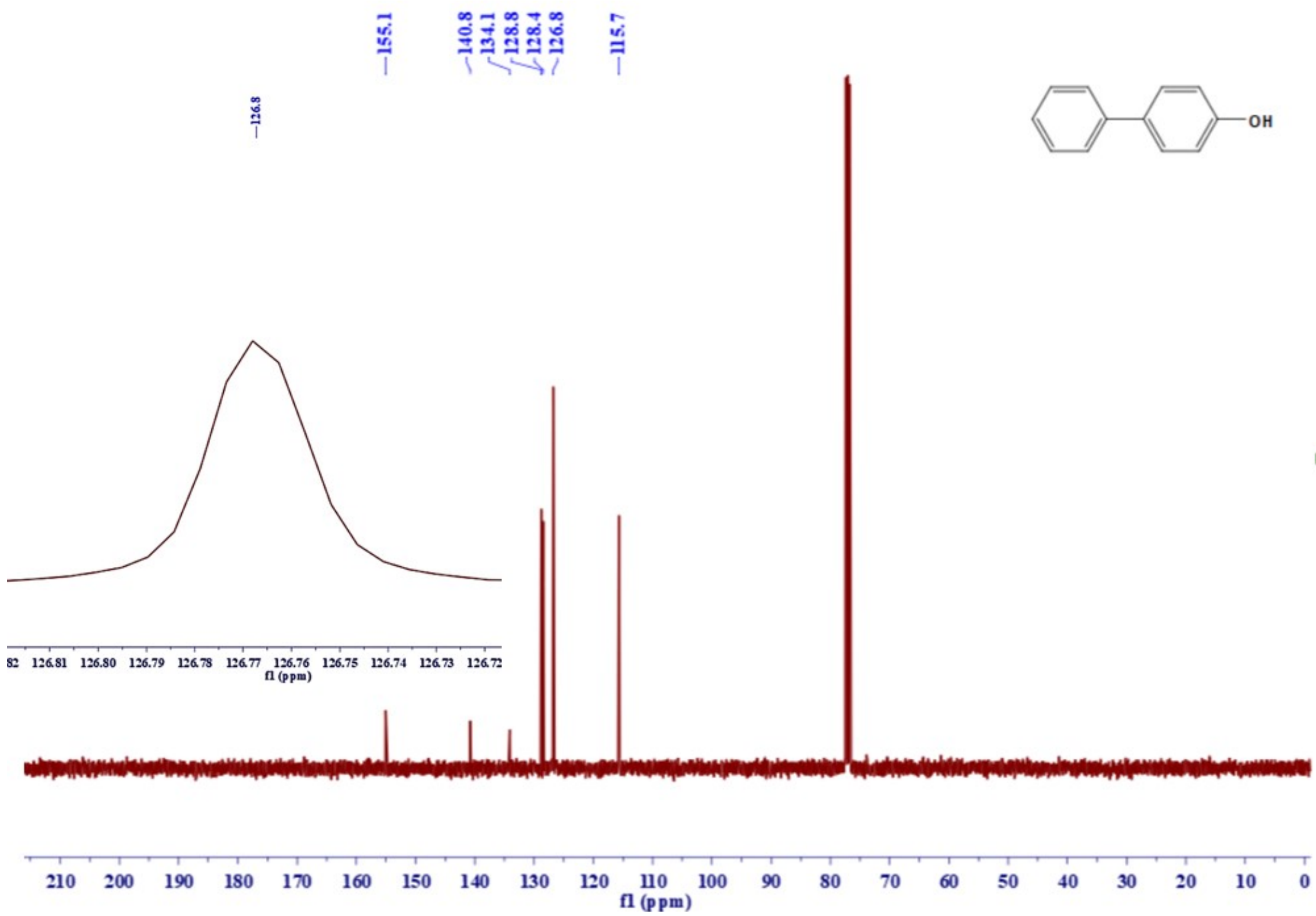

^{13}C NMR of [1,1'-biphenyl]-4-ol

[1,1'-biphenyl]-4-carbonitrile [3]

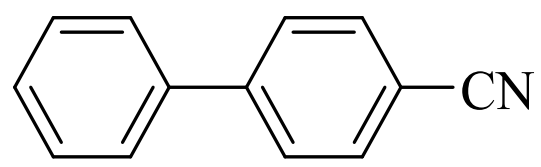

1H NMR (400 MHz, CDCl3) δ 7.78 – 7.71 (m, 4H), 7.64 – 7.62 (m, 2H), 7.55 – 7.44 (m, 3H).
^{13}C NMR (101 MHz, $CDCl_3$) δ 145.7, 139.2, 132.7, 129.2, 128.7, 127.8, 127.3, 119.0, 110.9.

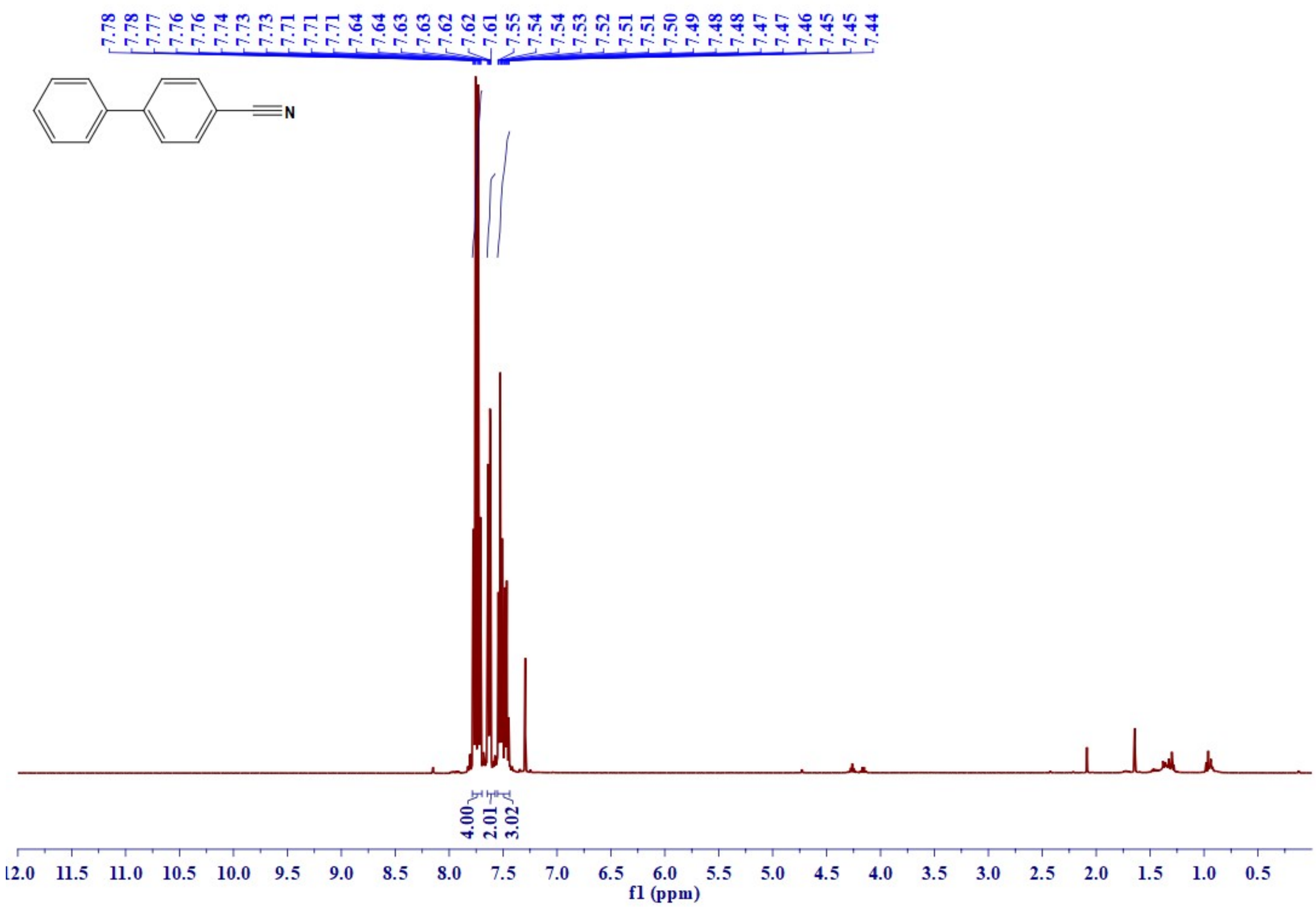

^{1}H NMR of [1,1'-biphenyl]-4-carbonitrile

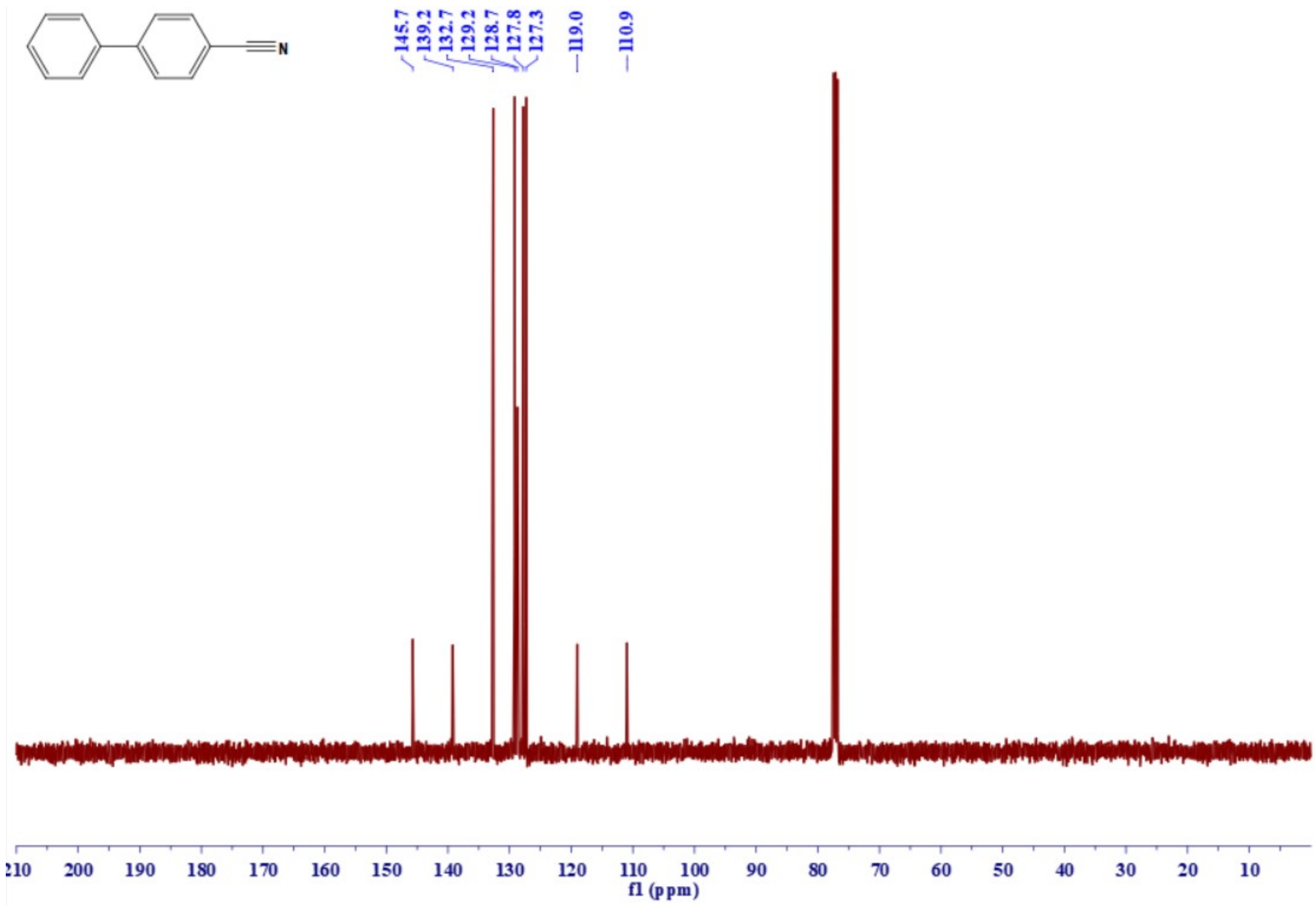

^{13}C NMR of [1,1'-biphenyl]-4-carbonitrile

4-nitro-1,1'-biphenyl [1]

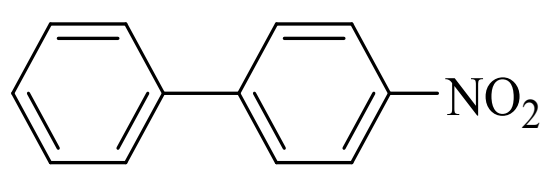

^{1}H NMR (400 MHz, $CDCl_3$) δ (ppm): 8.35 – 8.32 (m, 2H), 7.79 – 7.76 (m, 2H), 7.68 – 7.65 (m, 2H), 7.56 – 7.47 (m, 3H). ^{13}C NMR (100 MHz, $CDCl_3$) δ (ppm): 147.7, 147.1, 138.7, 129.2, 128.9, 127.8, 127.4, 124.2.

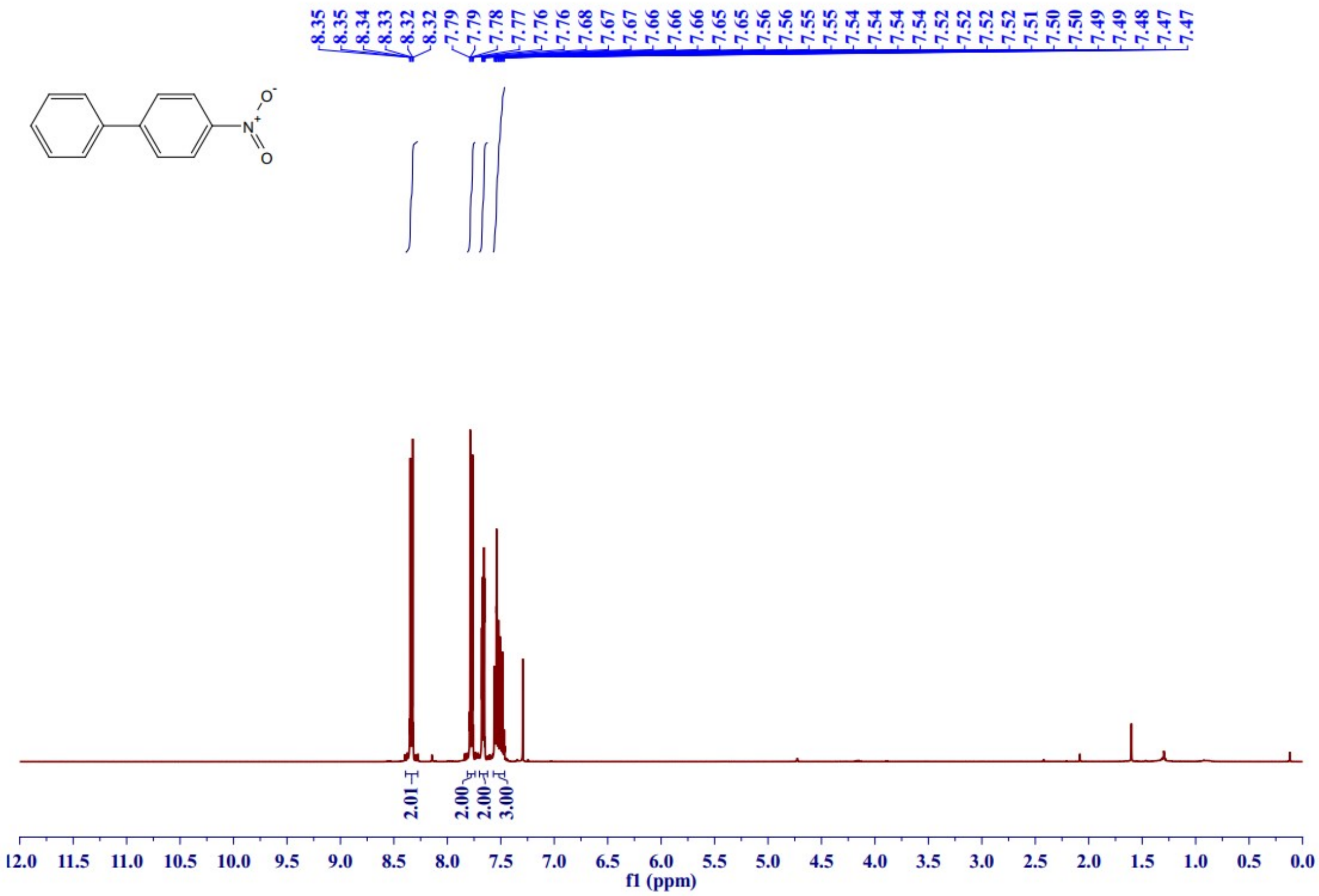

^{1}H NMR of 4-nitro-1,1'-biphenyl

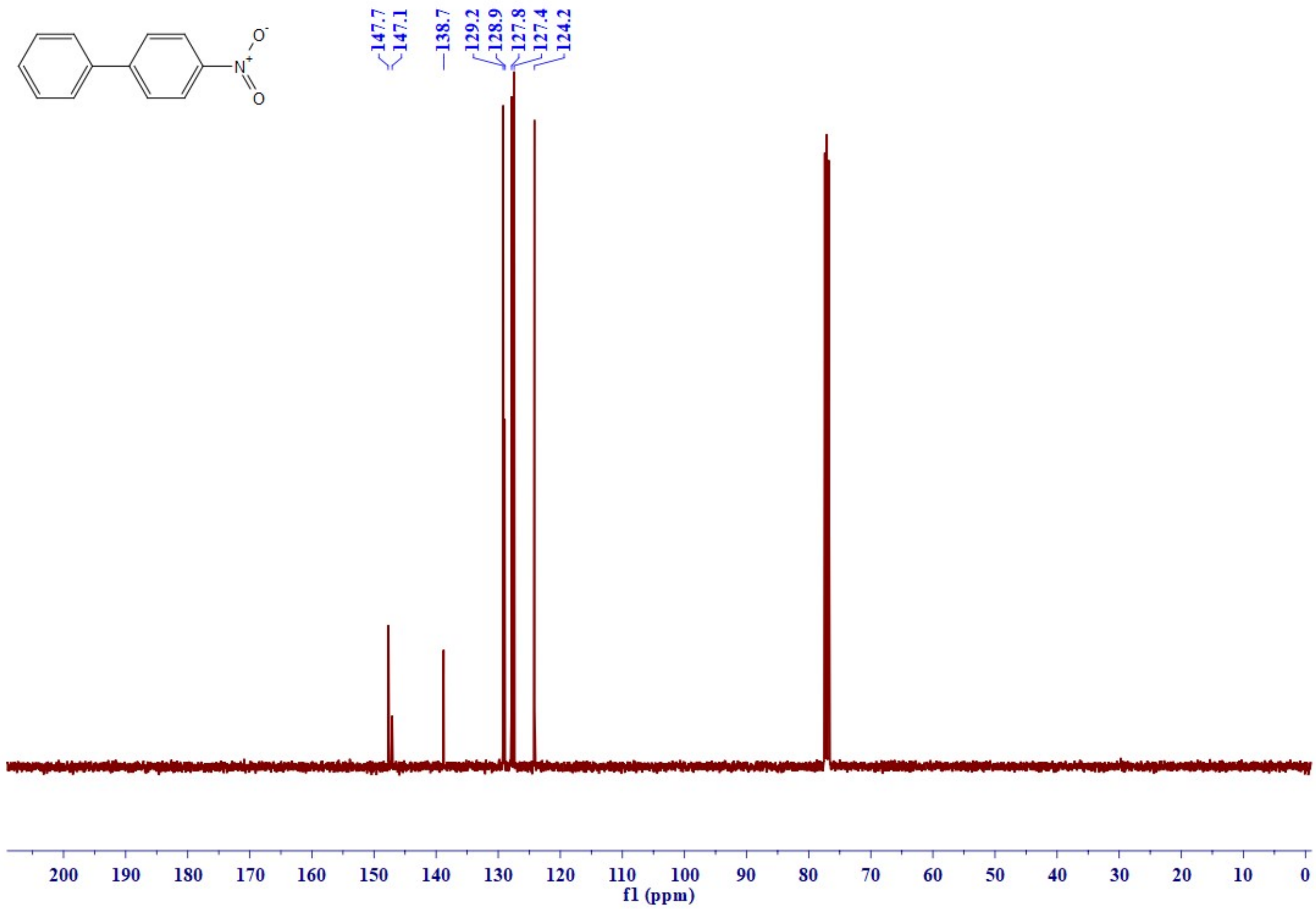

^{13}C NMR of 4-nitro-1,1'-biphenyl

1,1'4',1''-terphenyl [3]

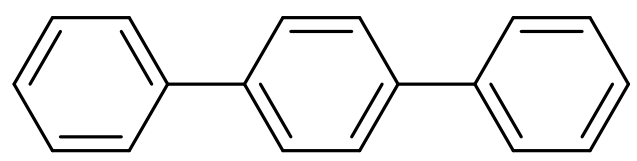

^{1}H NMR (400 MHz, $CDCl_3$) δ 7.72 (s, 4H), 7.72 – 7.66 (m, 4H), 7.54 – 7.48 (m, 4H), 7.44 – 7.37 (m, 2H). ^{13}C NMR (101 MHz, $CDCl_3$) δ 140.7, 140.1, 128.9, 127.6, 127.4, 127.1.

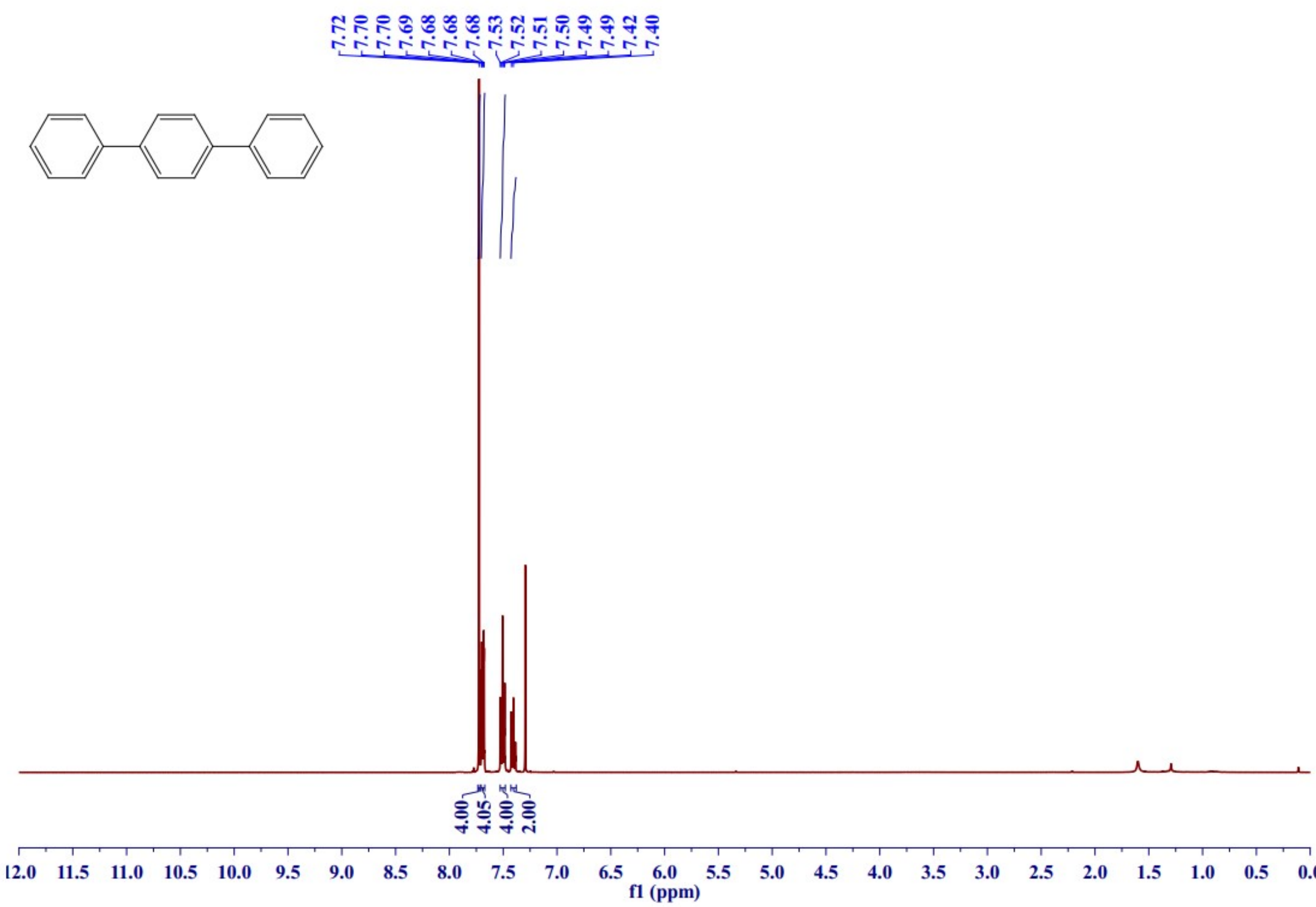

^{1}H NMR of 1,1'4',1''-terphenyl

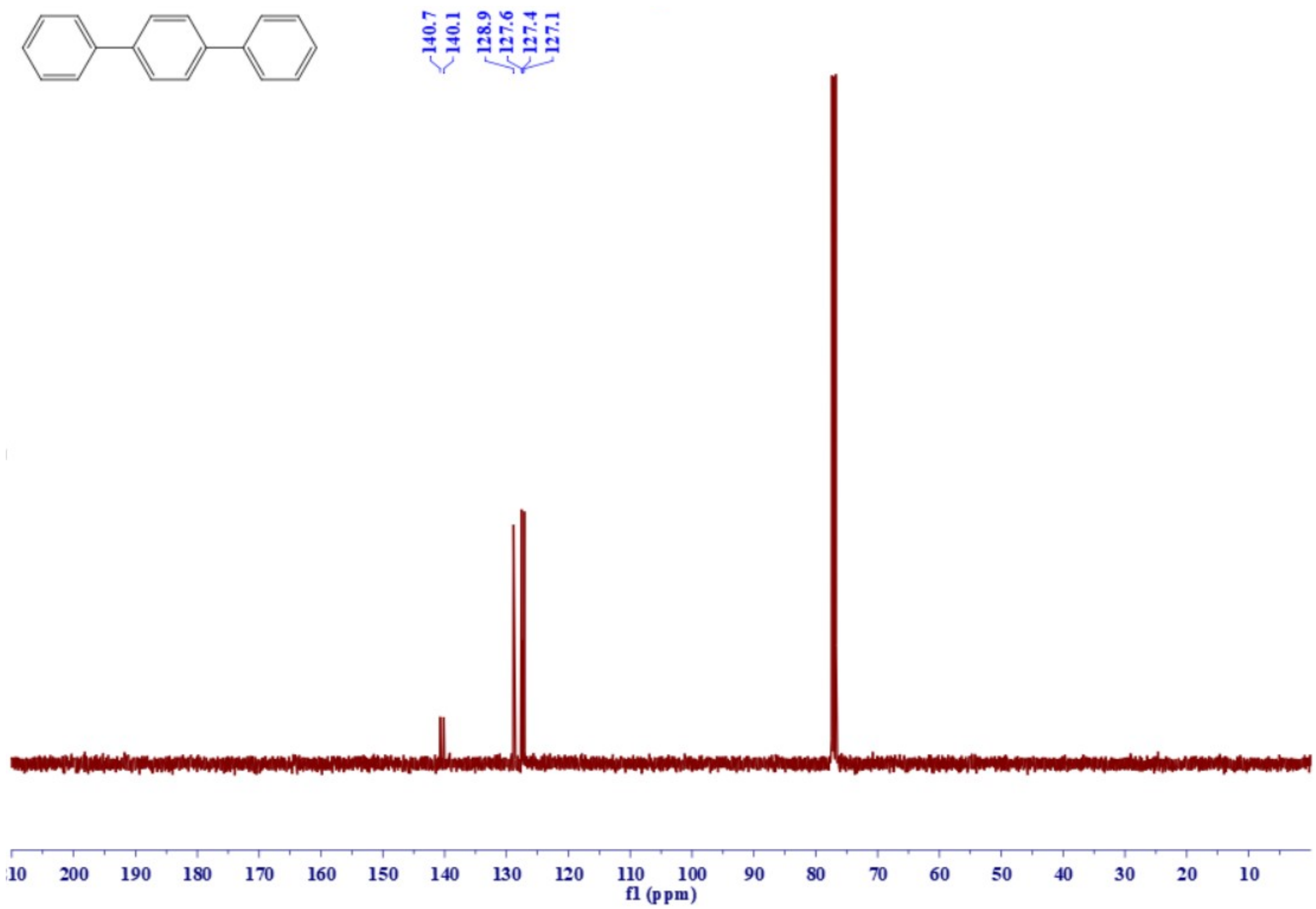

^{13}C NMR of 1,1'4',1''-terphenyl

2-methyl-5-nitro-1,1'-biphenyl

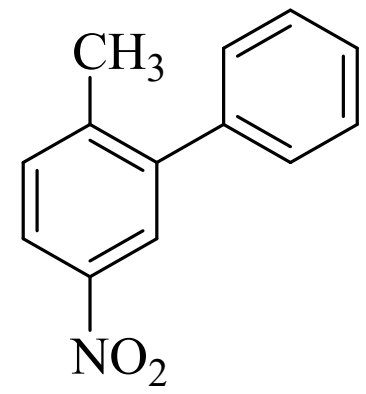

^{1}H NMR (400 MHz, $CDCl_3$) δ 8.16 – 8.13 (m, 2H), 7.52 – 7.45 (m, 4H), 7.37-7.34 (s, 2H), 2.40 (s, 3H). ^{13}C NMR (101 MHz, $CDCl_3$) δ 146.2, 143.6, 143.1, 139.6, 131.2, 129.0, 128.5, 127.9, 124.7, 122.1, 20.8.

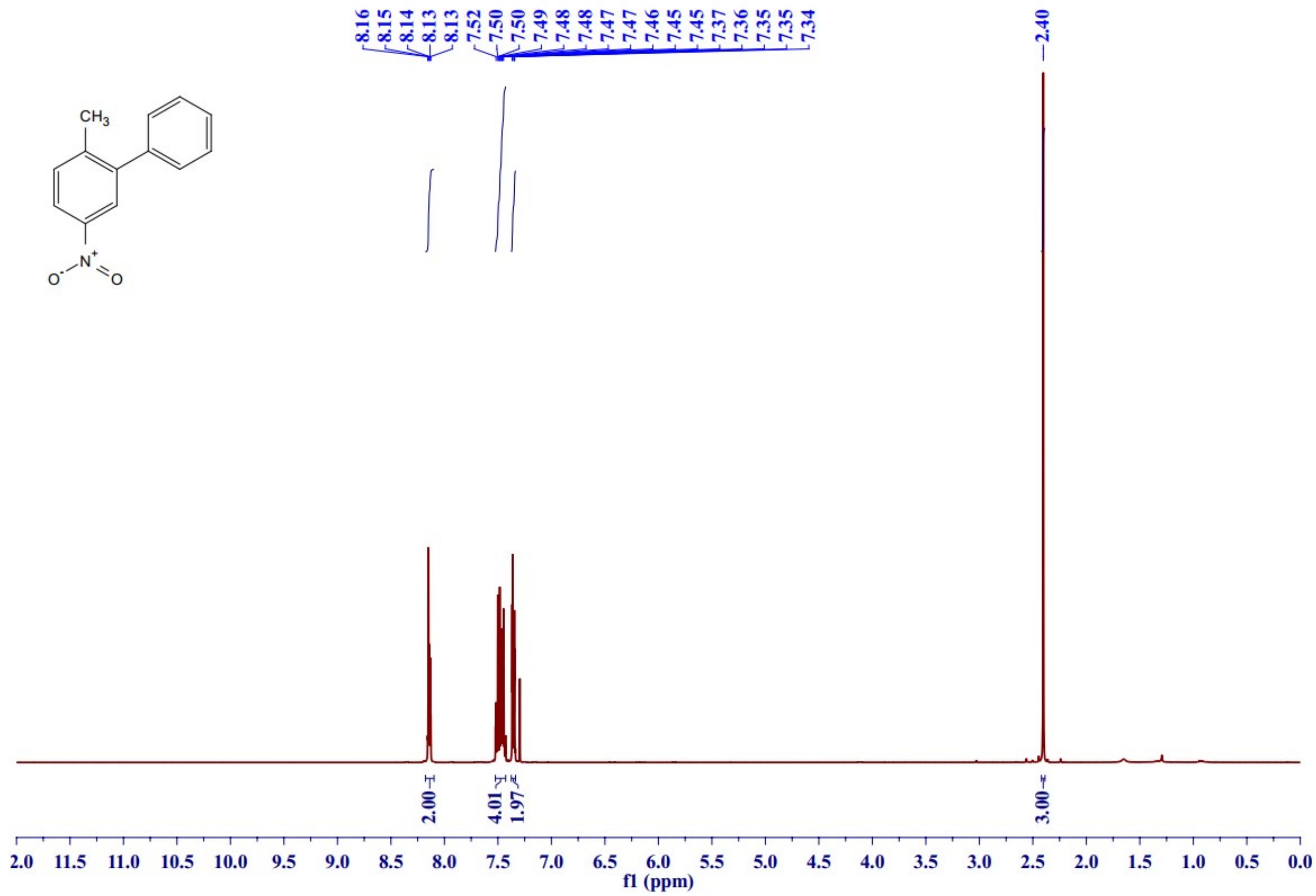

^{1}H NMR of 2-methyl-5-nitro-1,1'-biphenyl

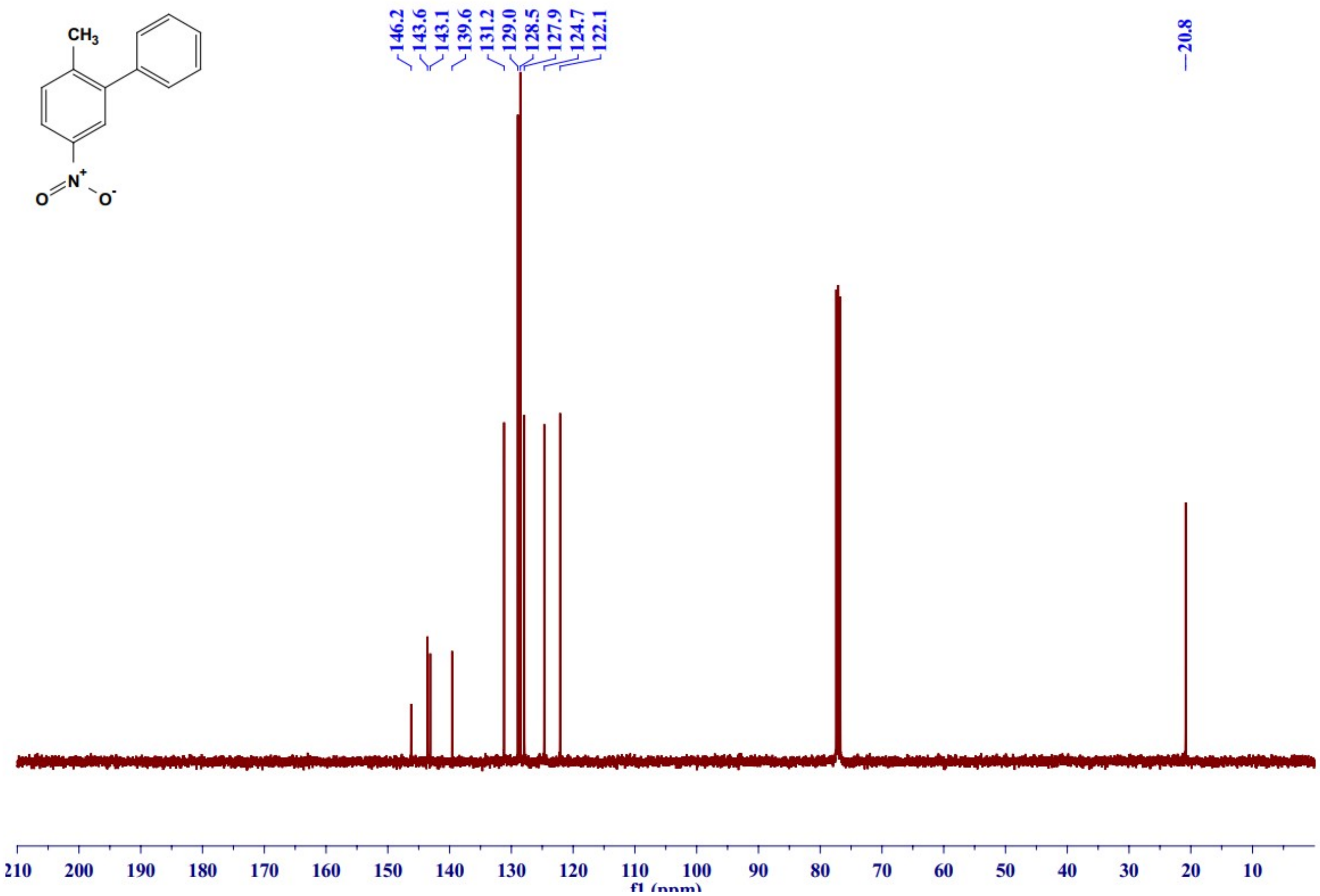

^{13}C NMR of 2-methyl-5-nitro-1,1'-biphenyl

Biphenyl-4-acetophenone [1]

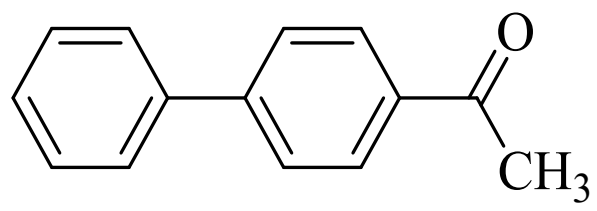

^{1}H NMR (400 MHz, $CDCl_3$) δ 8.07 (d, *J* = 8.6 Hz, 2H), 7.76 – 7.70 (m, 2H), 7.70 – 7.64 (m, 2H), 7.56 – 7.47 (m, 2H), 7.48 – 7.40 (m, 1H), 2.68 (s, 3H). ^{13}C NMR (101 MHz, $CDCl_3$) δ 197.9,145.8, 139.9, 135.9, 129.0, 128.9, 128.3, 127.3,127.2, 26.8.

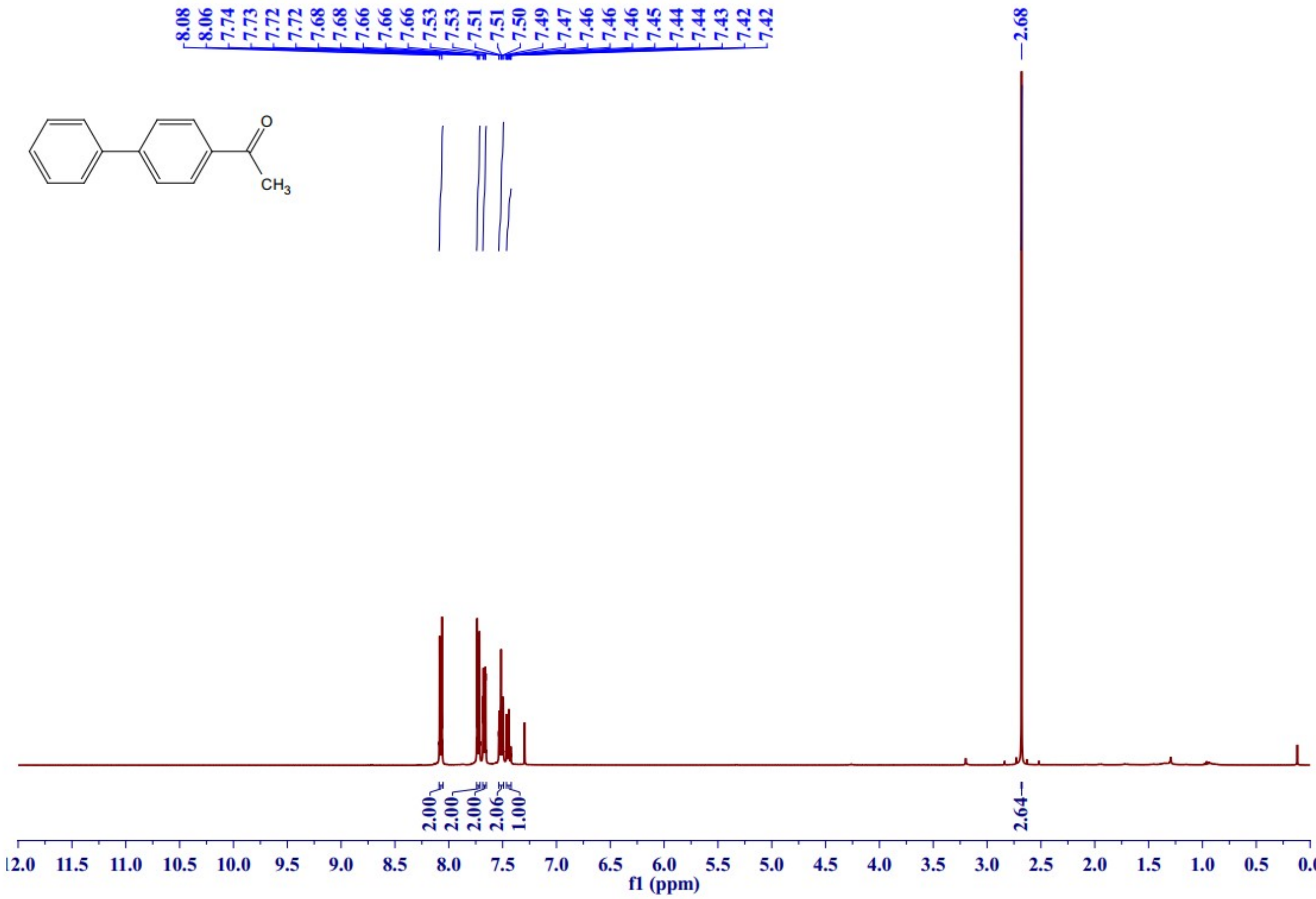

^{1}H NMR of Biphenyl-4-acetophenone

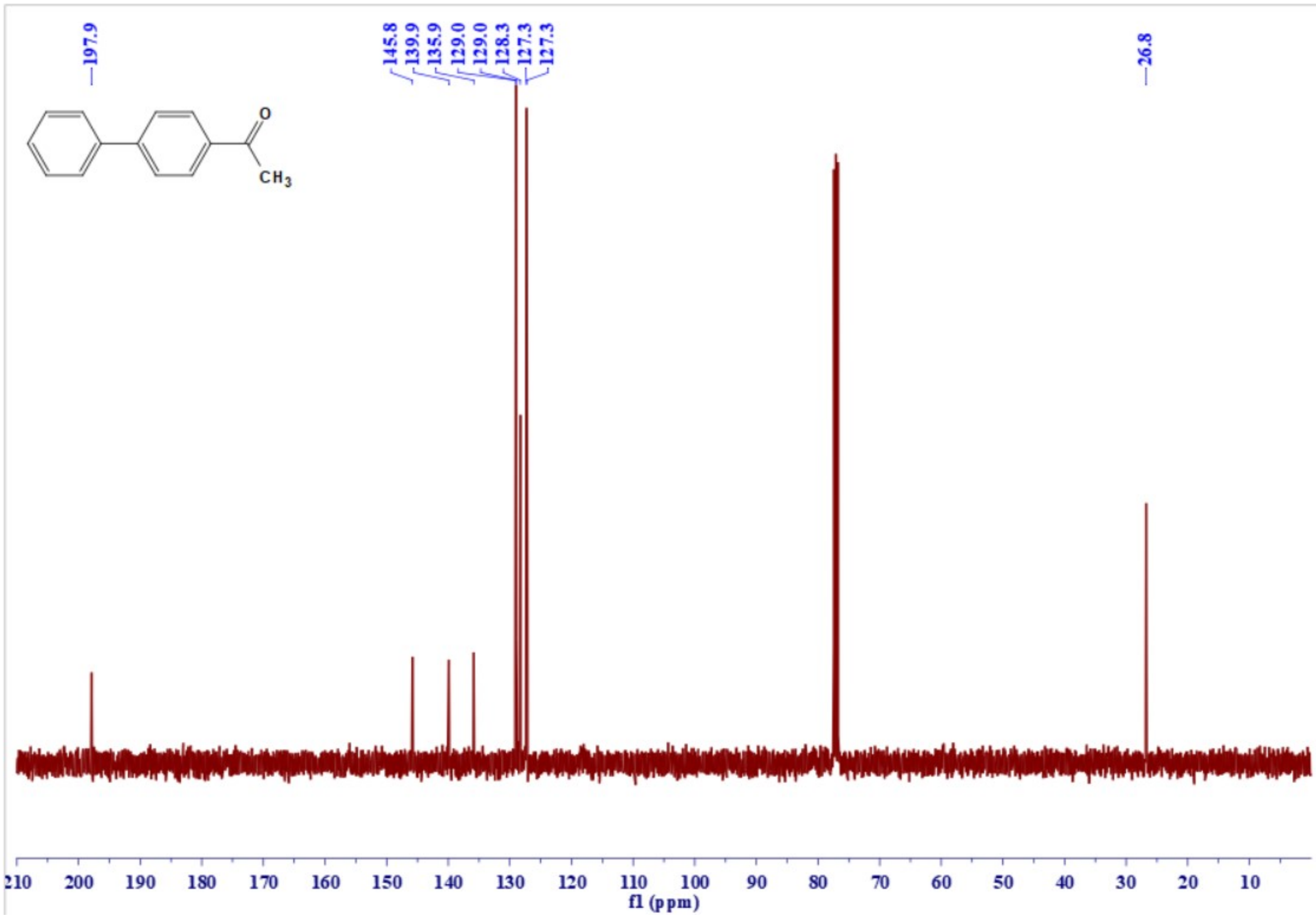

^{13}C NMR of Biphenyl-4-acetophenone

1-Phenylnaphthalene [11]

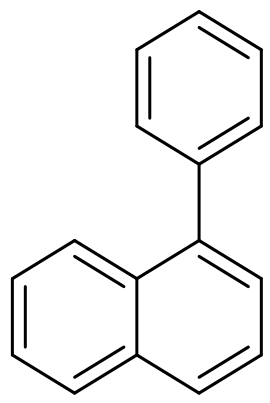

^1H NMR (400 MHz, $CDCl_3$) δ 7.96 – 7.89 (m, 3H), 7.59 – 7.45 (m, 9H). ^{13}C NMR (100 MHz, $CDCl_3$) δ (ppm): 140.8, 140.3, 133.8, 131.7, 130.1, 128.3, 127.7, 127.3, 127.0, 126.1, 125.8, 125.4.

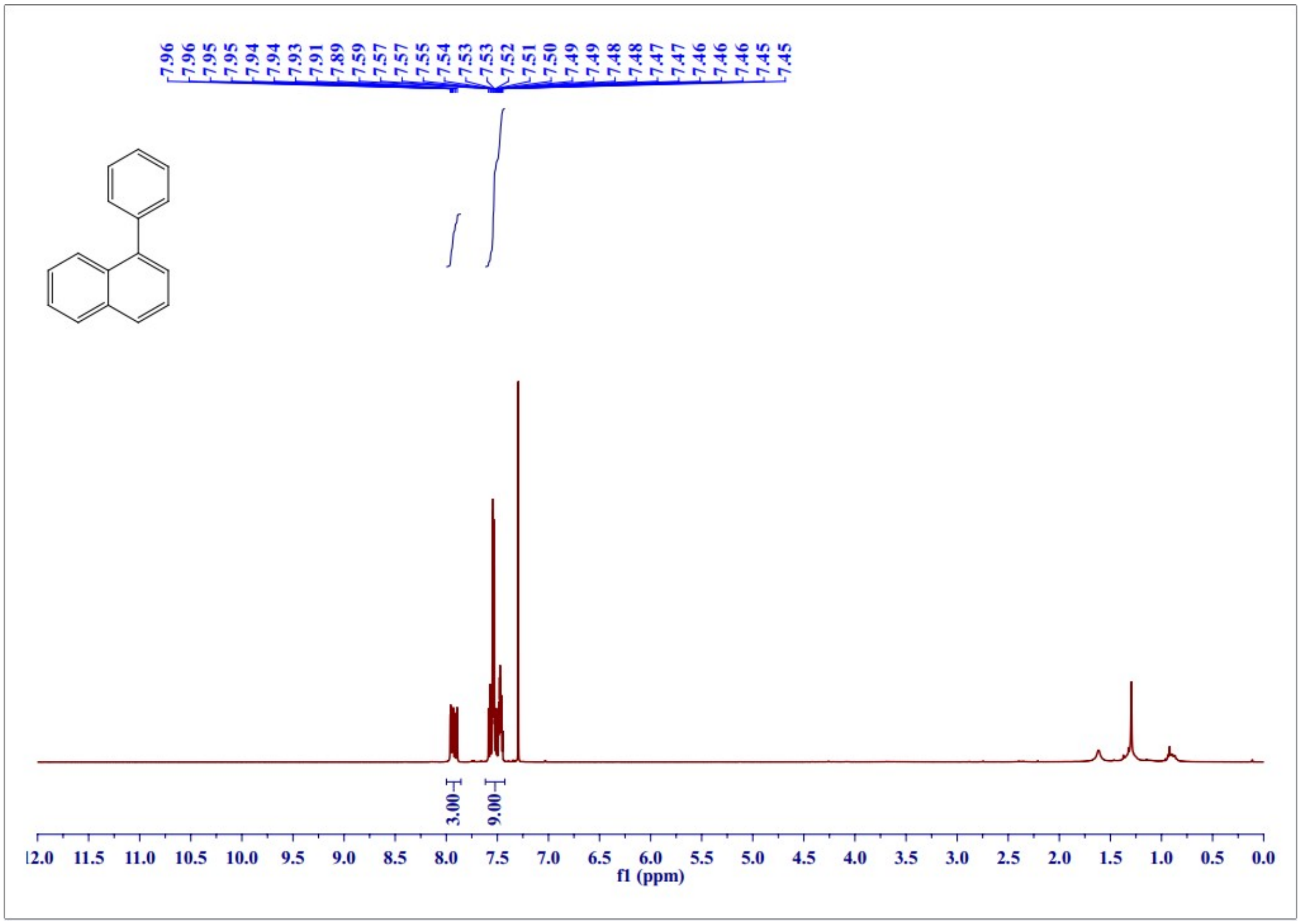

^1H NMR of 1-phenylnaphthalene

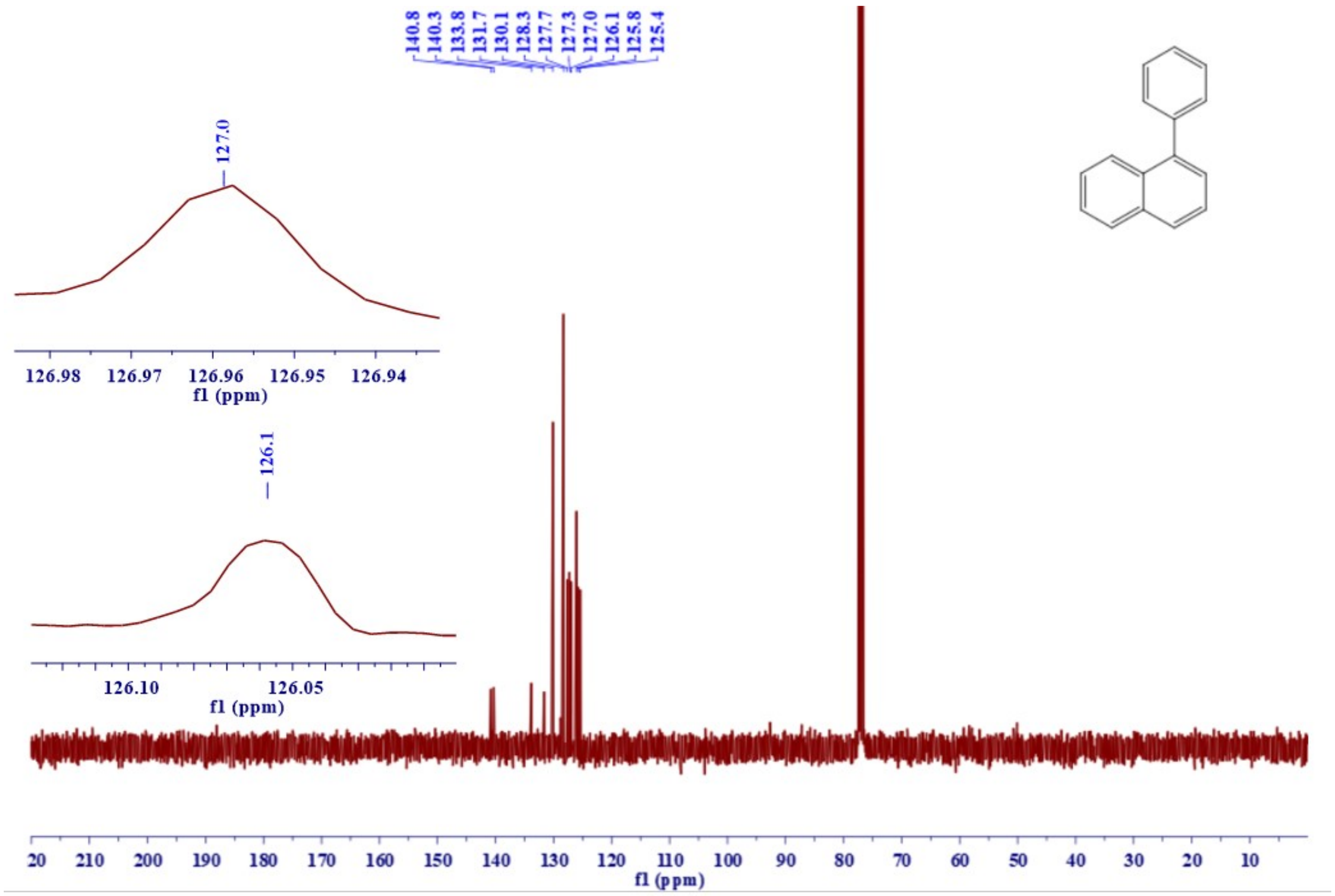

^{13}C NMR of 1-phenylnaphthalene

2-phenylthiophene[12]

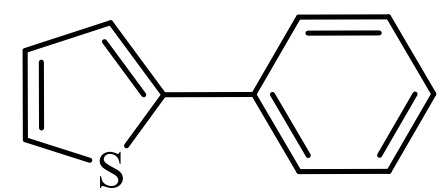

^{1}H NMR (400 MHz, $CDCl_3$) δ 7.67 – 7.65 (m, 2H), 7.44 – 7.34 (m, 2H), 7.33 – 7.30 (m, 2H), 7.14 – 7.12 (m, 2H). ^{13}C NMR (100 MHz, $CDCl_3$) δ 144.5, 134.4, 128.9, 128.0, 127.5, 126.0, 124.8, 123.1.

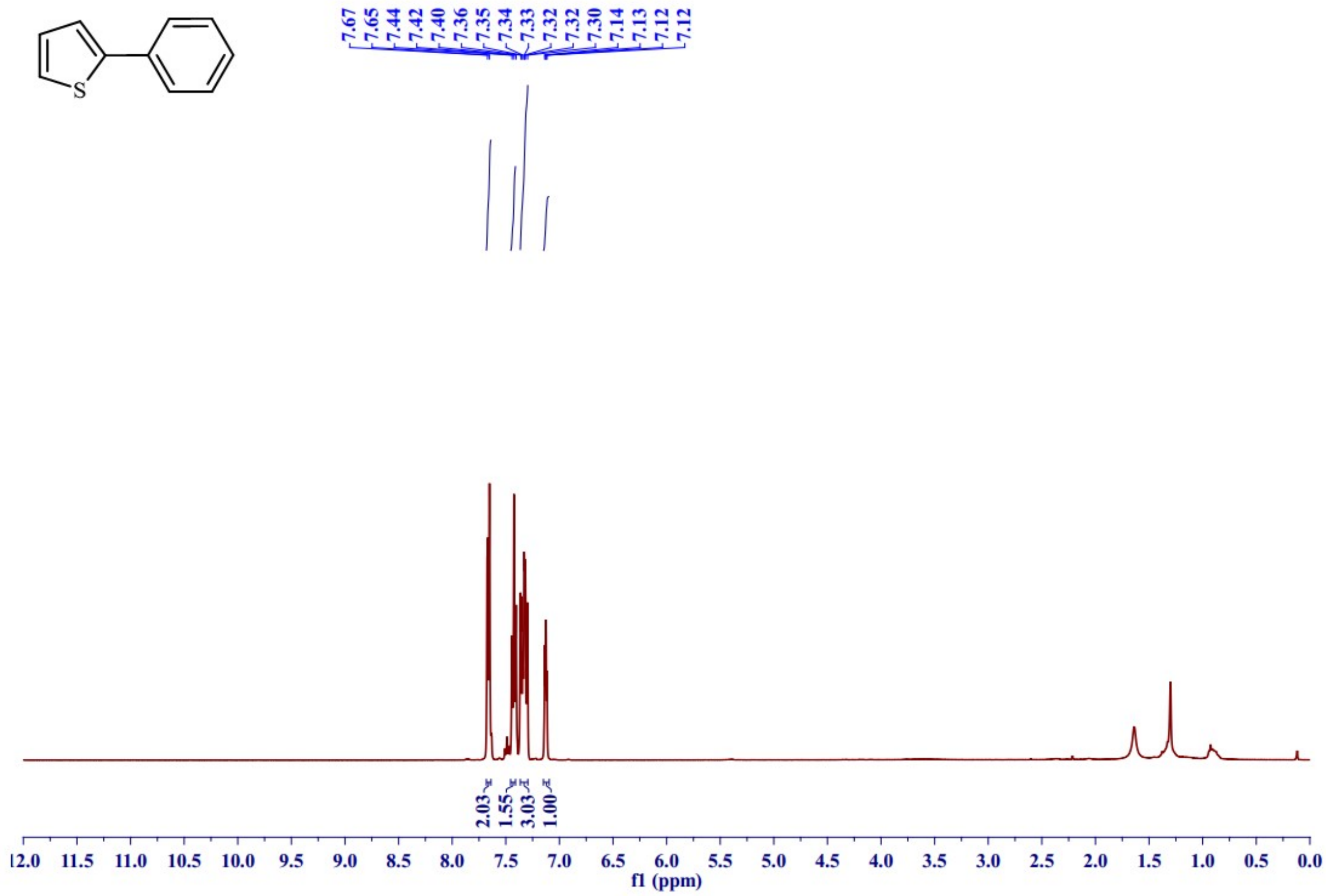

^{1}H NMR of 2-Phenylthiophene

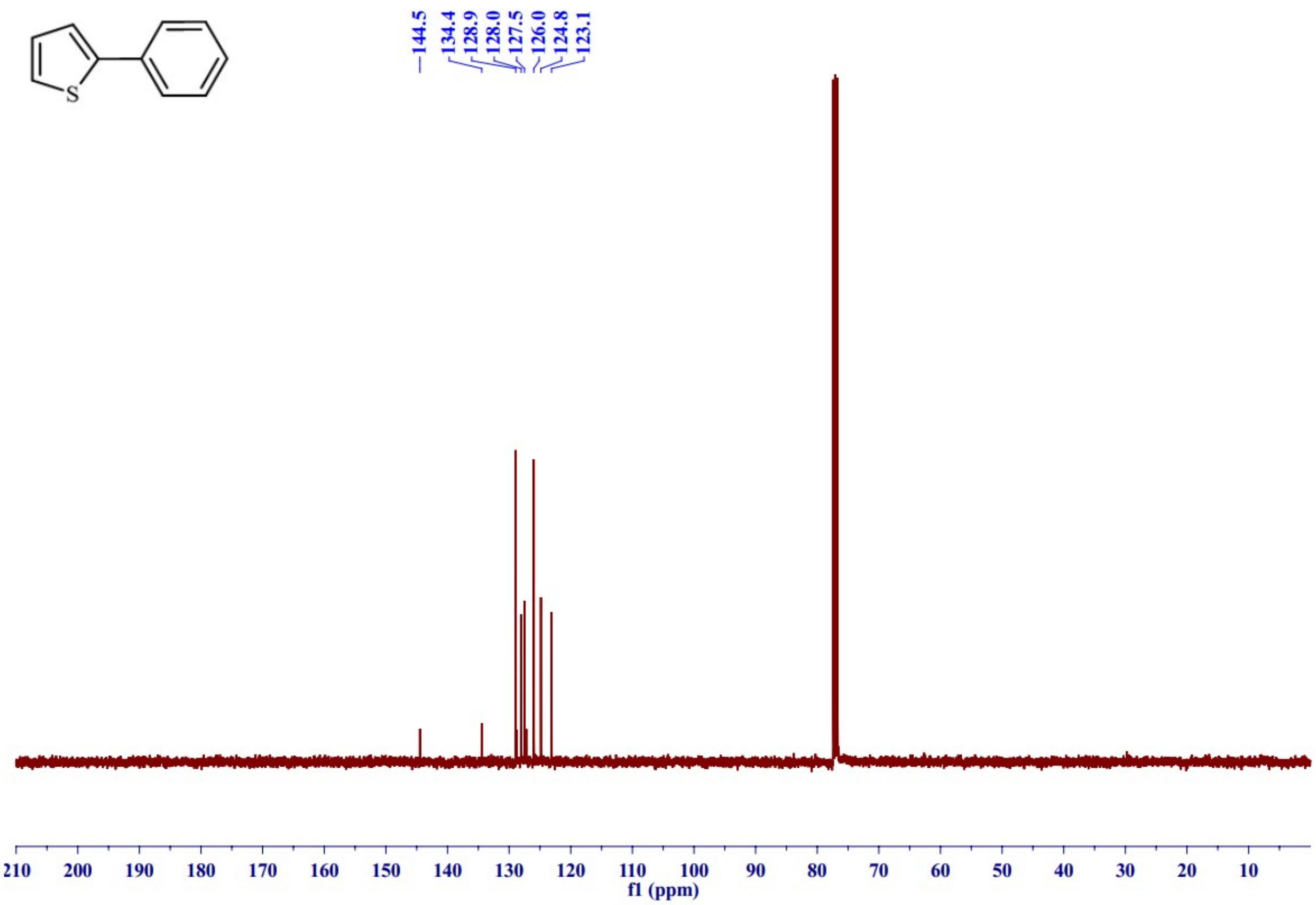

^{13}C NMR of 2-Phenylthiophene

4-Fluoro-1,1'-biphenyl [8]

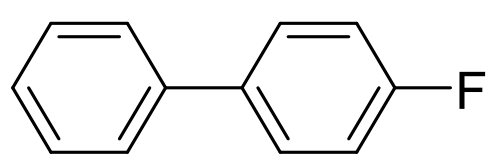

^{1}H NMR (400 MHz, $CDCl_3$) δ 7.63 – 7.57 (m, 4H), 7.51 – 7.46 (m, 2H), 7.42 – 7.37 (m, 1H), 7.21 – 7.15 (m, 2H). ^{13}C NMR (100 MHz, $CDCl_3$) δ 162.5 (d, J = 242.4 Hz), 140.3, 137.4 (d, J = 3.03 Hz), 128.9, 128.8, 128.7 (d, J = 8.08 Hz), 127.3, 127.1, 115.7 (d, J = 21.21 Hz).

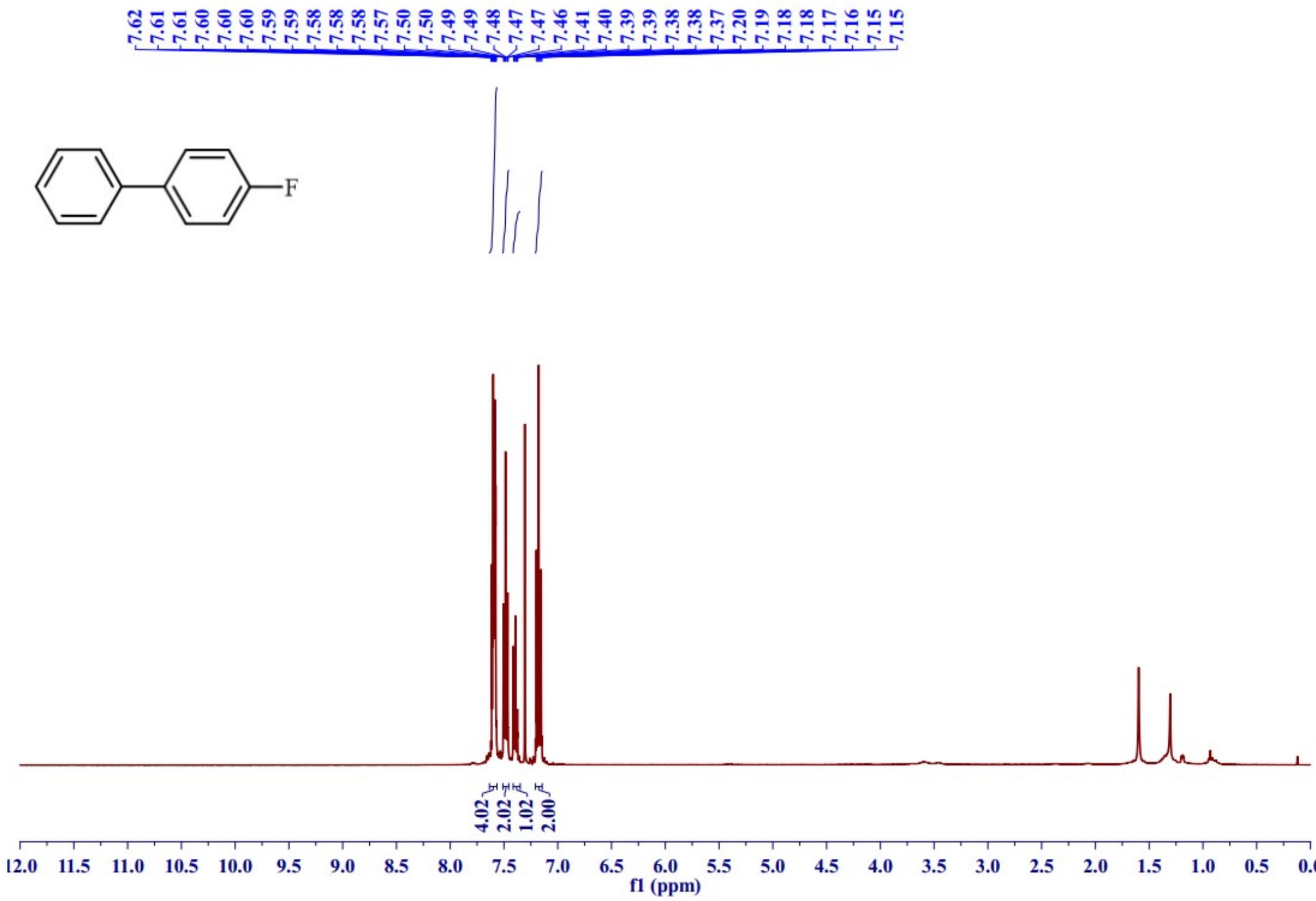

^{1}H NMR of 4-Fluoro-1,1'-biphenyl

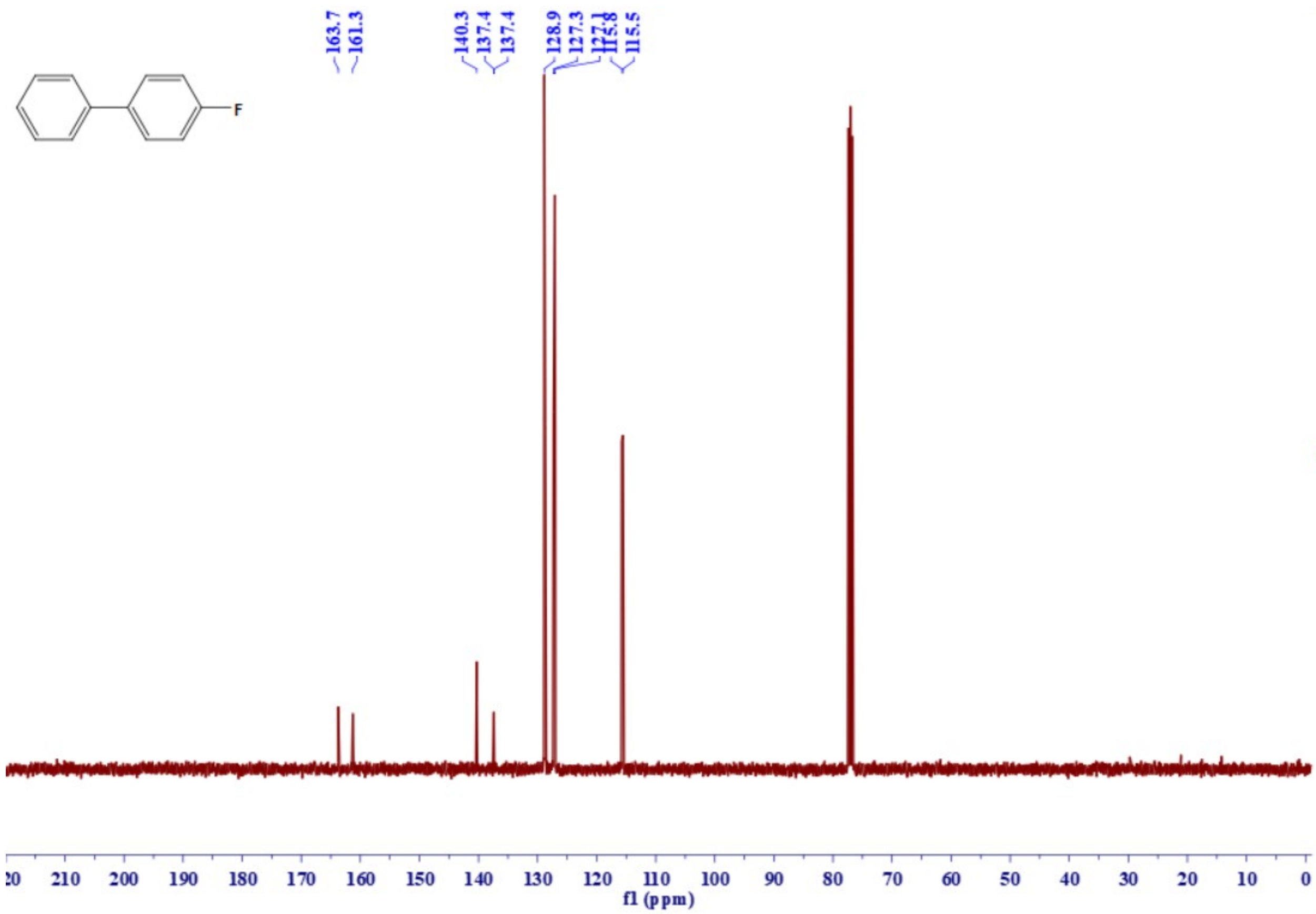

^{13}C NMR of 4-Fluoro-1,1'-biphenyl

5-Phenylpyrimidine [3]

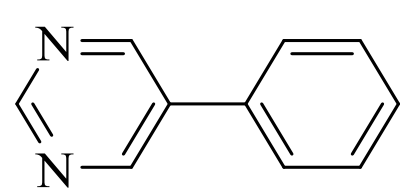

^{1}H NMR (400 MHz, $CDCl_3$) δ (ppm): 9.25 (s, 1H), 9.01 (s, 2H), 7.63 – 7.49 (m, 5H). ^{13}C NMR (100 MHz, $CDCl_3$) δ (ppm): 157.2, 154.9, 134.4, 134.1, 129.5, 129.1, 127.1.

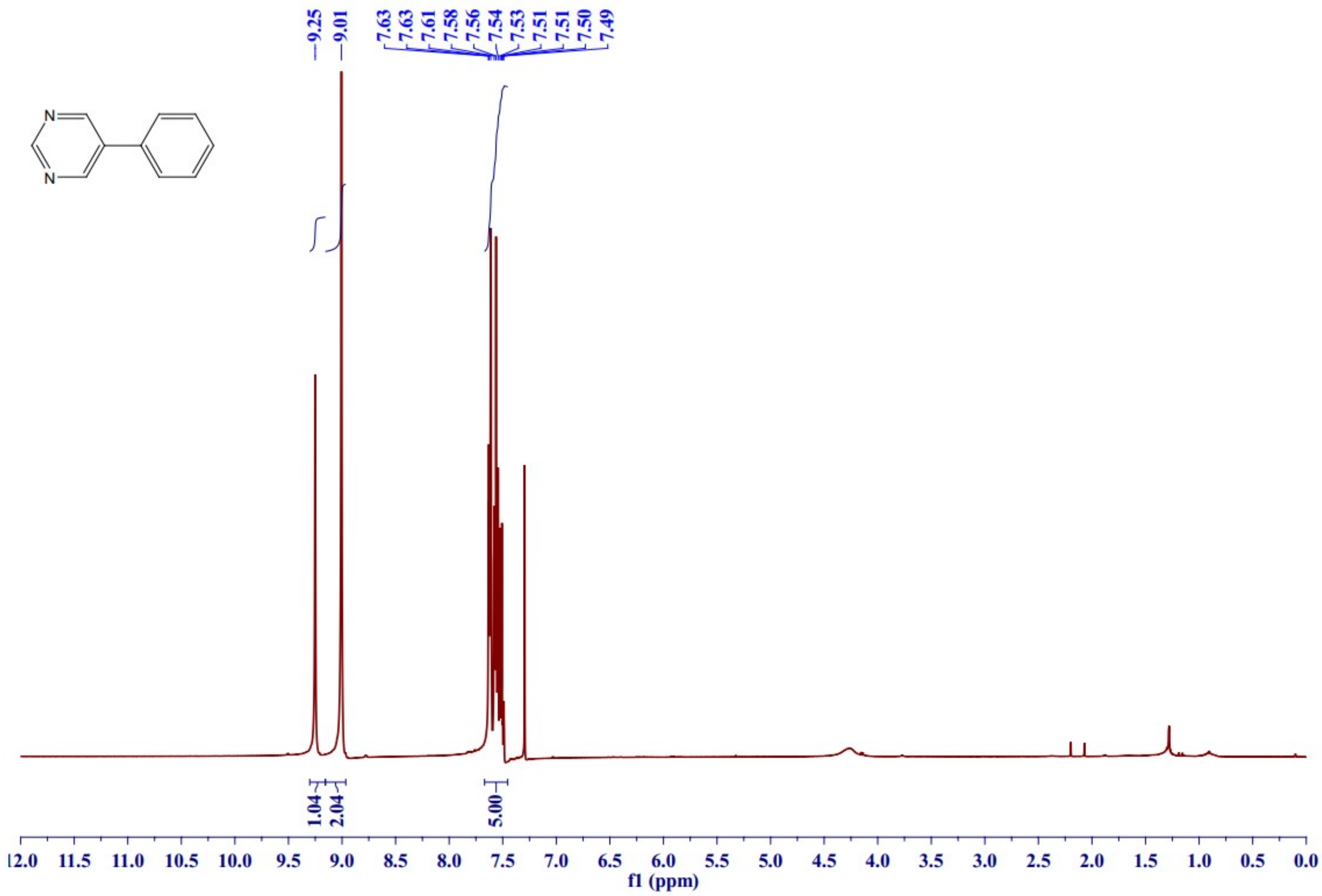

^{1}H NMR of 5-Phenylpyrimidine

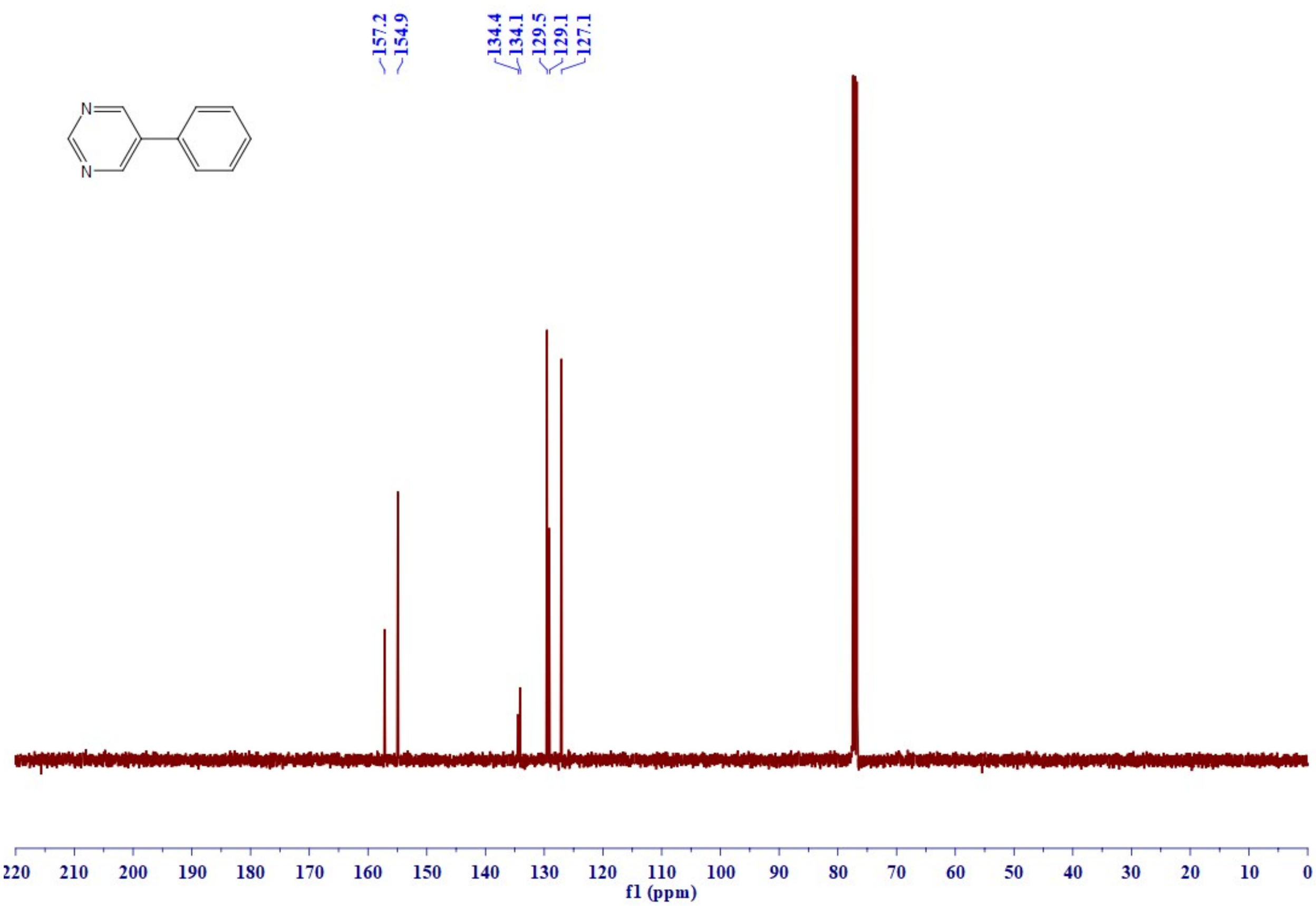

^{13}C NMR of 5-Phenylpyrimidine

3'-fluoro-[1,1'-biphenyl]-4-carbonitrile

^{1}H NMR (400 MHz, $CDCl_3$) δ 7.78 (d, *J* = 8.0 Hz, 2H), 7.71 (d, *J* = 8.4 Hz, 2H), 7.49 (m, 1H), 7.43 – 7.39 (m, 1H), 7.34 – 7.29 (m, 1H), 7.19 – 7.12 (m, 1H).^{13}C NMR (100 MHz, $CDCl_3$) δ (ppm): 164.5, 162.0, 144.3 (d, *J* = 2.02 Hz), 141.4 (d, *J* = 7.07 Hz), 132.7 , 130.8 , 130.7 , 127.8 , 123.0 (d, *J* = 3.03 Hz), 118.7 , 115.5 (d, *J* = 21.21 Hz), 114.2(d, *J* = 23.23 Hz), 111.6 .

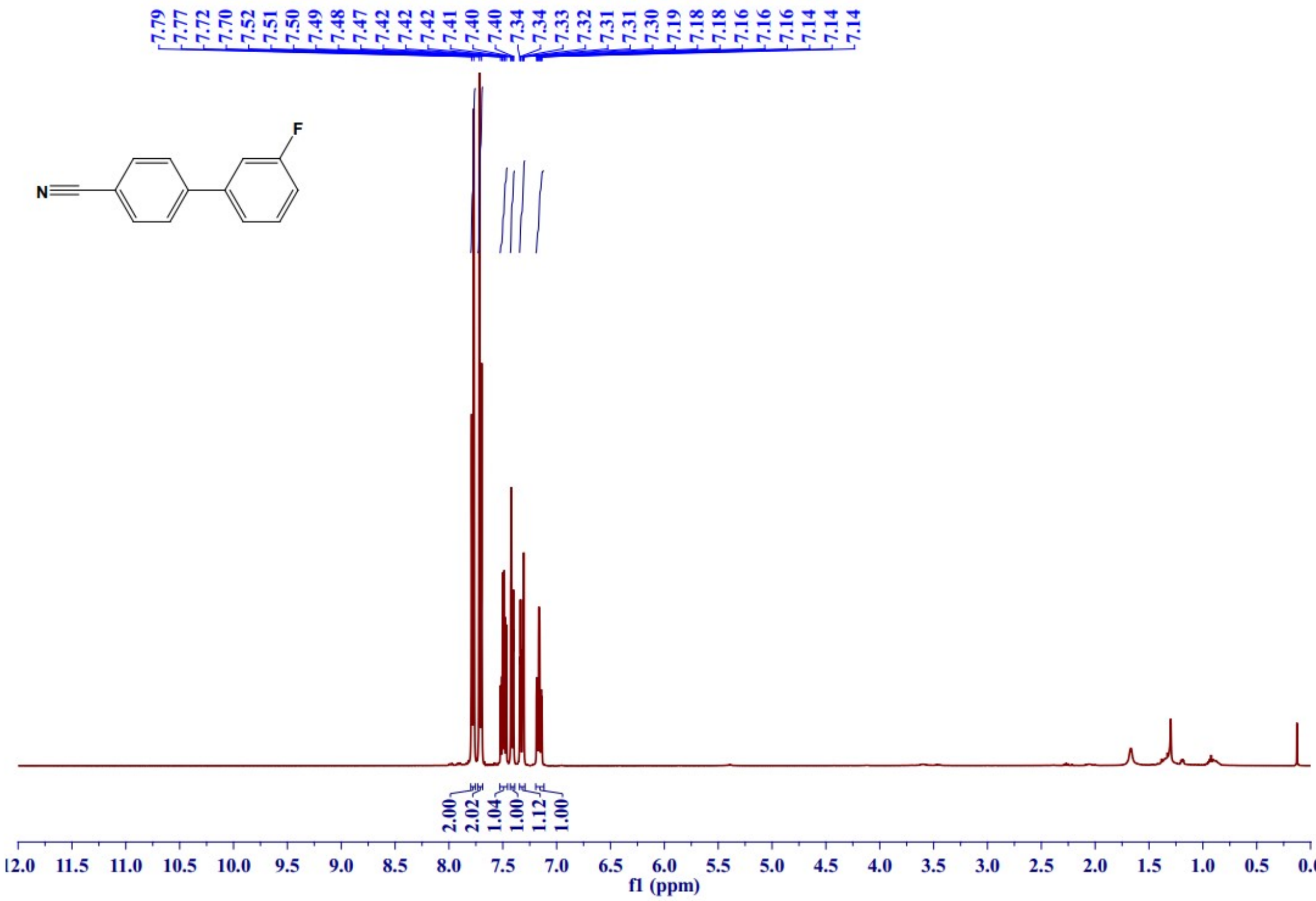

^{1}H NMR of 3'-fluoro-[1,1'-biphenyl]-4-carbonitrile

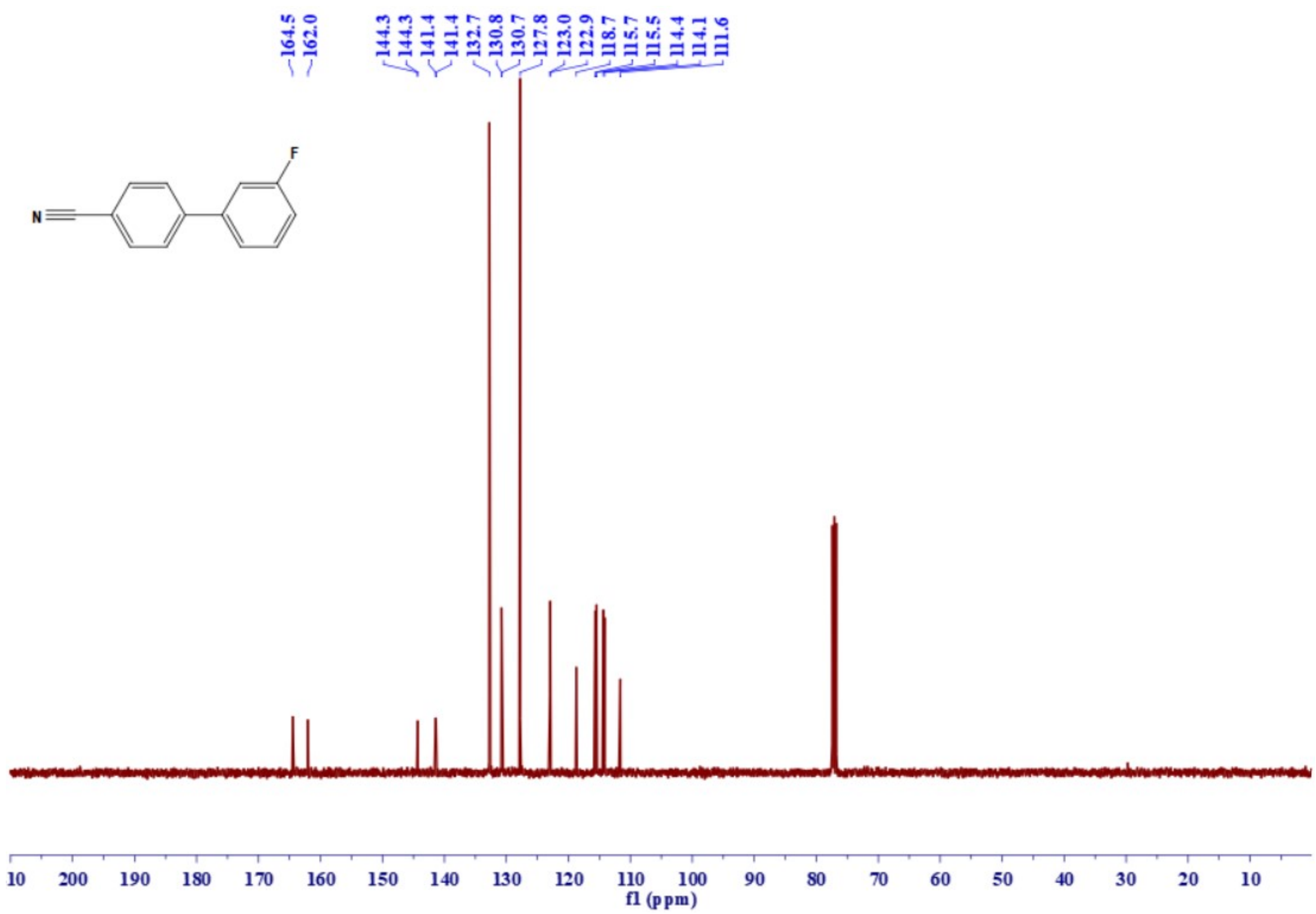

^{13}C NMR of 3'-fluoro-[1,1'-biphenyl]-4-carbonitrile

4'-methyl-[1,1'-biphenyl]-4-carbaldehyde

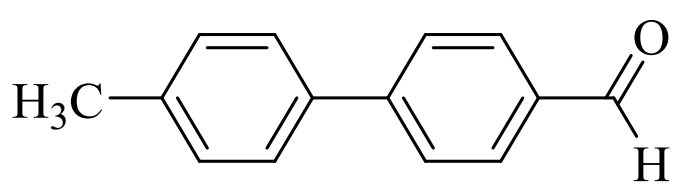

^{1}H NMR (400 MHz, $CDCl_3$) δ (ppm): 10.08 (s, 1H), 8.00 – 7.95 (m, 2H), 7.78 (d, *J* = 8.3 Hz, 2H), 7.60 – 7.57 (m, 2H), 7.33 (d, *J* = 7.9 Hz, 2H), 2.45 (s, 3H).^{13}C NMR (101 MHz, $CDCl_3$) δ 192.0, 147.2, 138.6, 136.8,135.0, 130.3, 129.8, 127.5 ,127.2, 21.2.

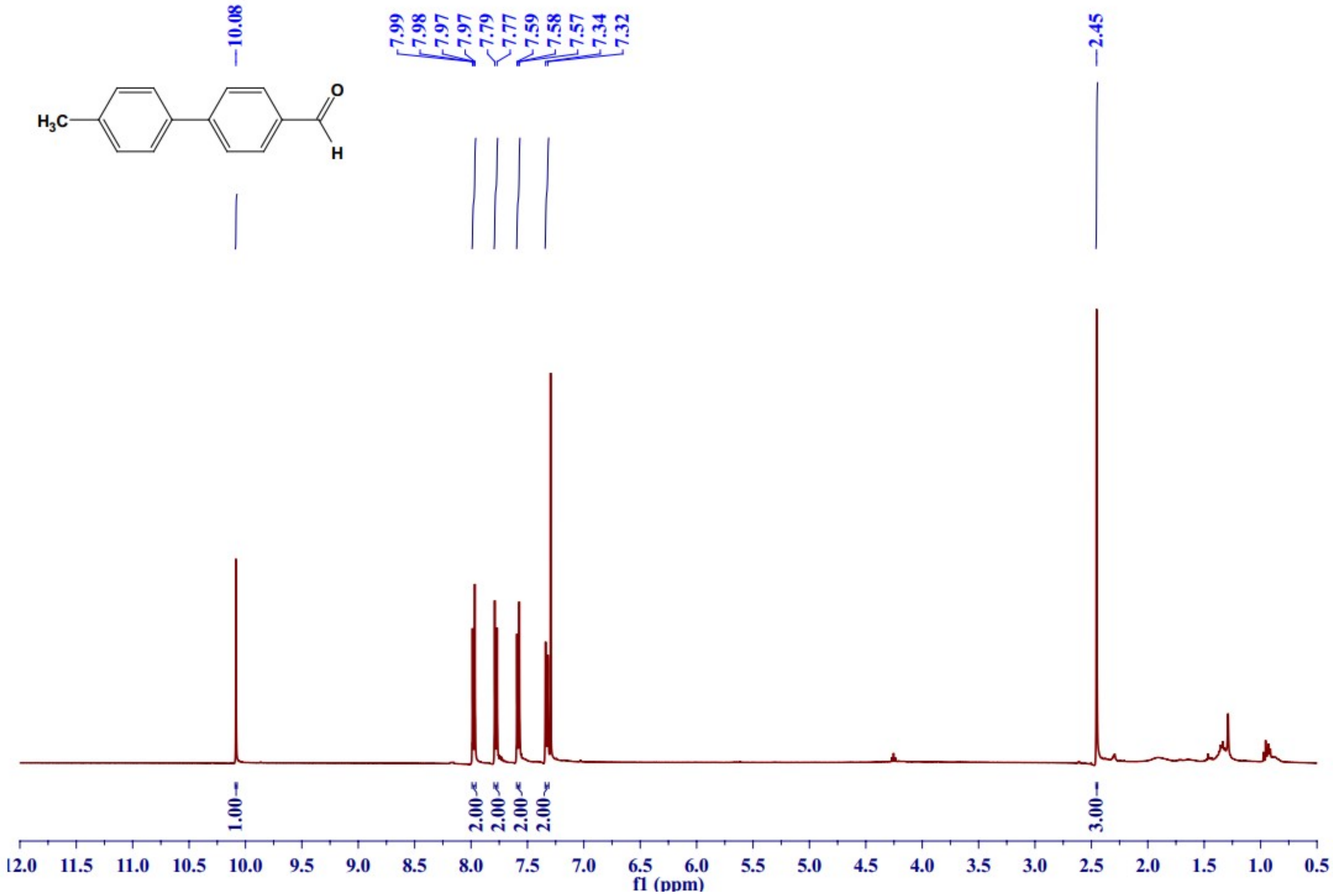

^{1}H NMR of 4'-methyl-[1,1'-biphenyl]-4-carbaldehyde

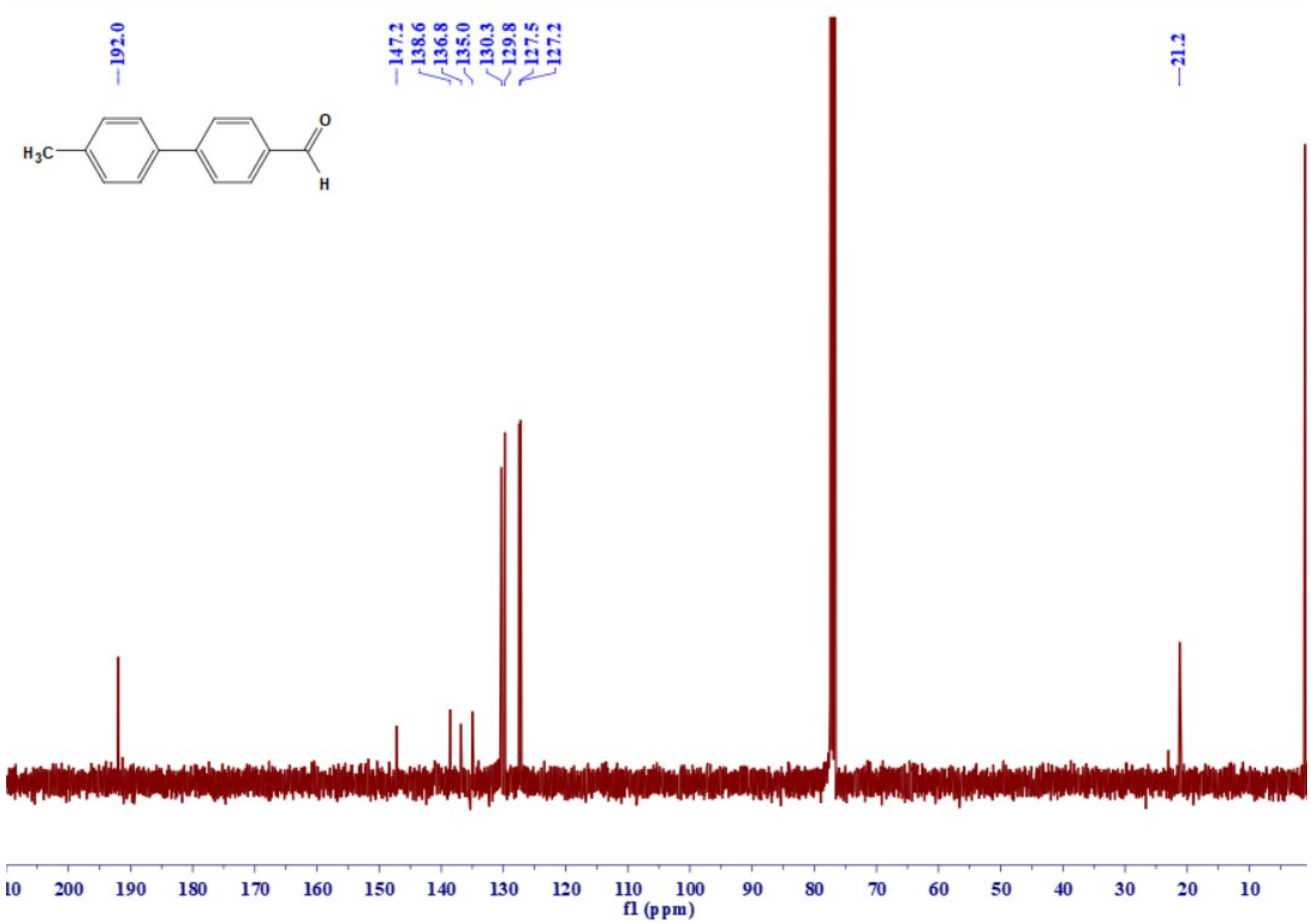

^{13}C NMR of 4'-methyl-[1,1'-biphenyl]-4-carbaldehyde

2,4,5-trimethyl-1,1-biphenyl

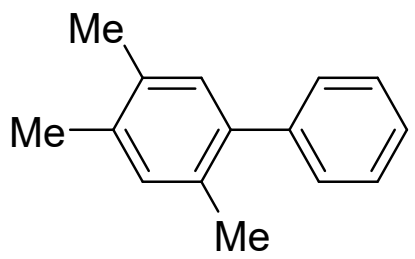

^{1}H NMR (400 MHz, $CDCl_3$) δ (ppm): 7.47 – 7.44 (m, 2H), 7.39 – 7.30 (m, 3H), 7.12 – 7.09 (d, 2H), 2.34 – 2.33 (d, 6H), 2.28 (s, 3H). ^{13}C NMR (101 MHz, $CDCl_3$) δ 142.0 (s), 139.5, 135.5, 133.8, 132.5, 131.8, 131.2, 129.3, 128.0, 126.6, 19.8, 19.4 ,19.2.

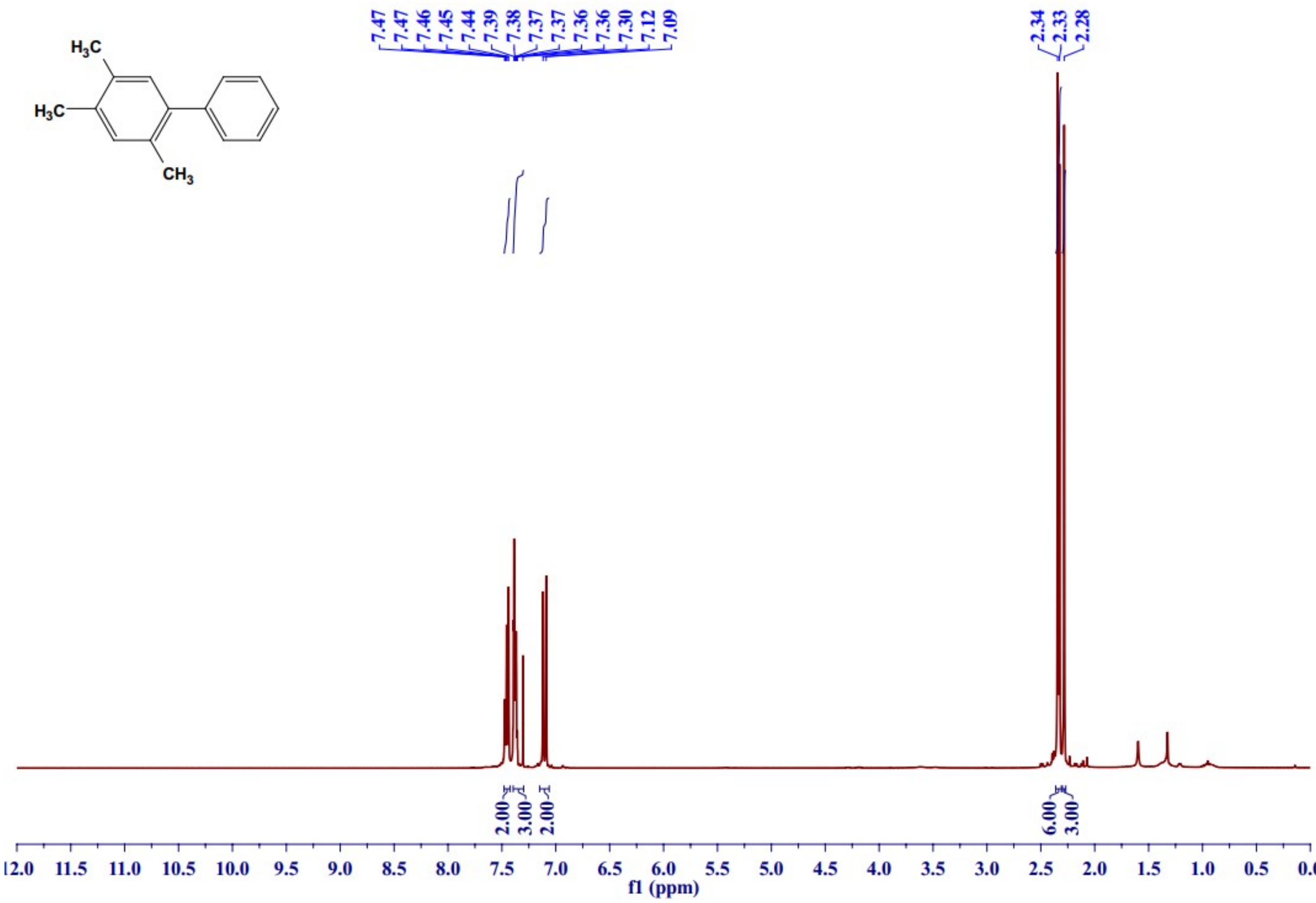

^{1}H NMR of 2,4,5-trimethyl-1,1-biphenyl

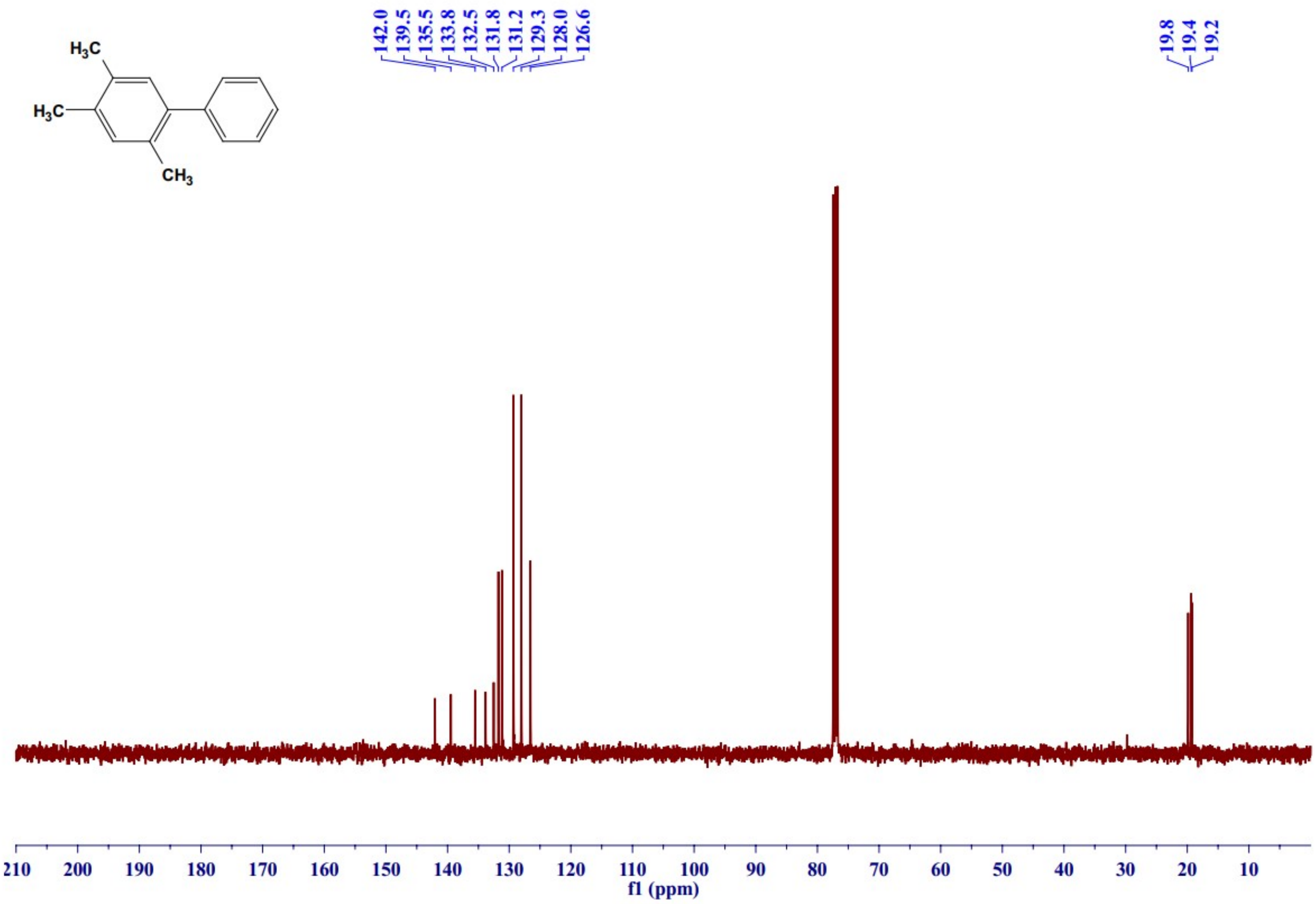

^{13}C NMR of 2,4,5-trimethyl-1,1-biphenyl

2-methyl-1-phenylnaphthalene

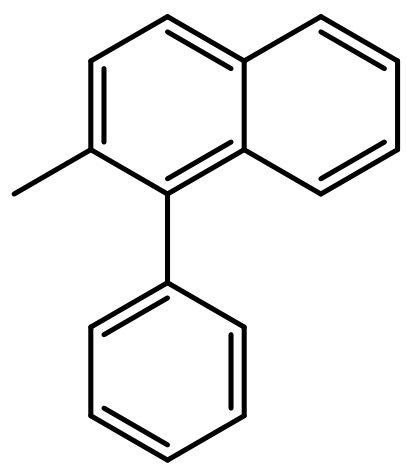

^{1}H NMR (400 MHz, $CDCl_3$) δ 7.93 – 7.84 (m, 2H), 7.59 – 7.35 (m, 9H), 2.33 (s, 3H). ^{13}C NMR (101 MHz, $CDCl_3$) δ 139.9, 138.2, 133.2, 133.0, 132.0, 130.2, 128.7, 127.8, 127.3, 127.1, 126.2, 125.9, 124.8, 20.9.

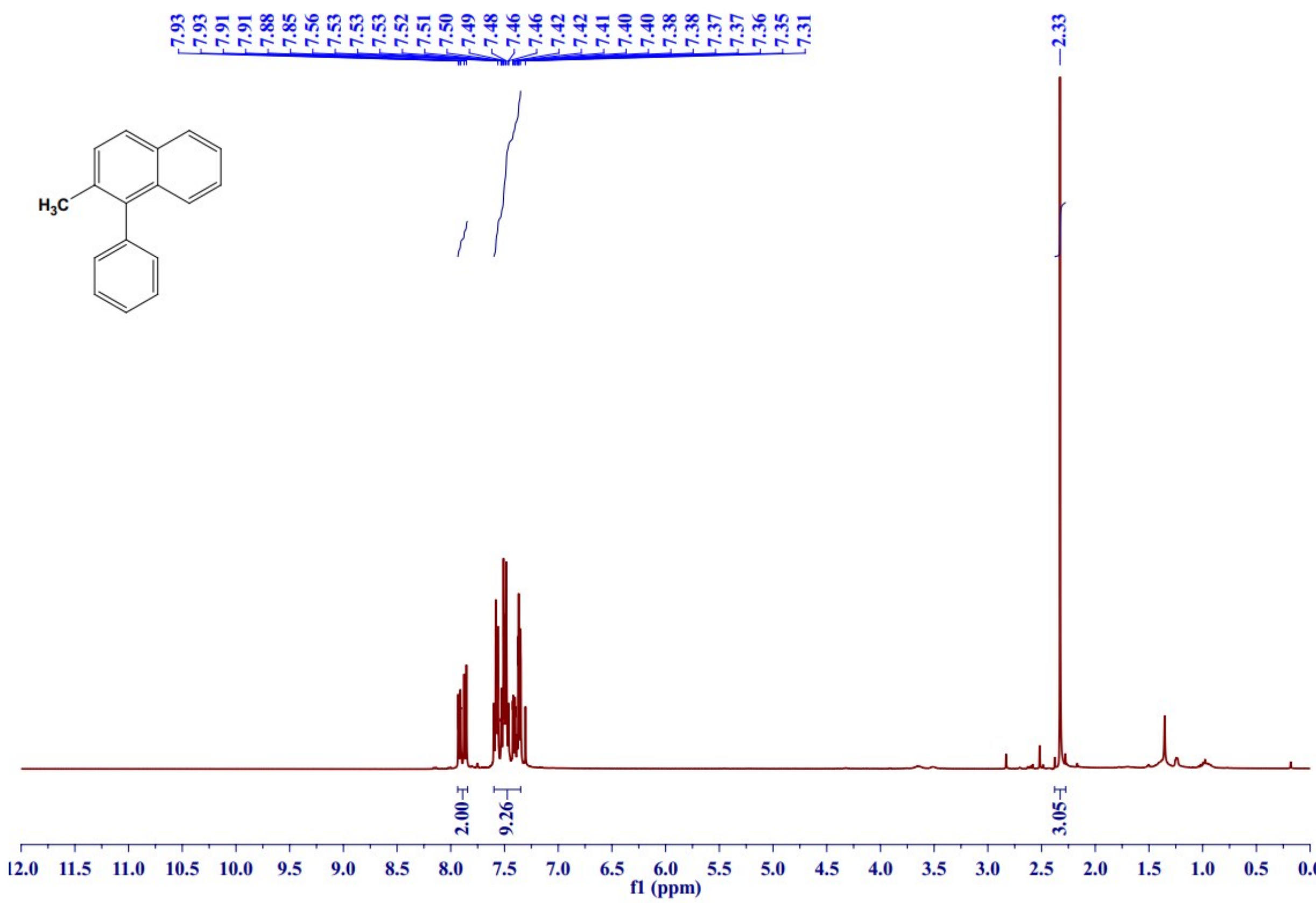

^{1}H NMR of 2-methyl-1-phenylnaphthalene

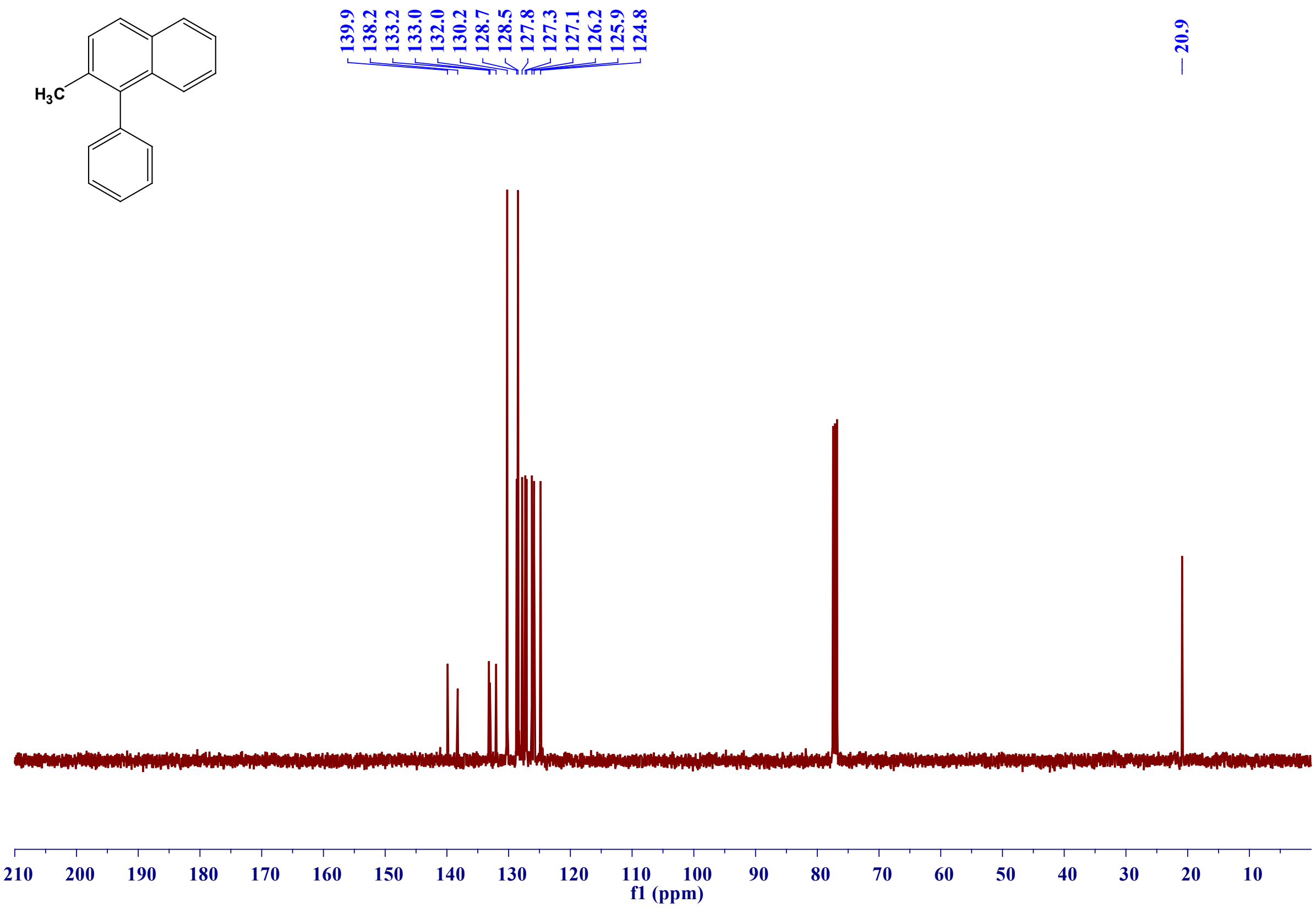

^{13}C NMR of 2-methyl-1-phenylnaphthalene

References:

1-X. Zhang, *Clickphosphines for Transition Metal-Catalyzed Reactions*, US 7,709,655 B2, 2010.

2- Parvin Sanati-Tirgan, H. Eshghi, A. Mohammadinezhad, *Appl. Organomet. Chem.*, 2024, DOI: 10.1002/aoc.7487.

2- U. Kazmaier, S. Haehn, T. D. Weiss, R. Kautenburger, W. F. Maier, Synlett. 2007, 2007, 2579-2583, DOI: 10.1055/s-2007-986639

3-X. Cheng, W. Li, R. Nie, X. Ma, R. Sang, L. Guo, Y. Wu, Adv. Synth. Catal., 2017, 359, 454-466, DOI: 10.1002/adsc.201600815.

4-K. V. Kutonova, N. Jung, M. E. Trusova, V. D. Filimonov, P. S. Postnikov, S. Bräse, Synth., 2017, 49, 1680-1688, DOI: 10.1055/s-0036-1588919

5- A. L. Isfahani, I. Mohammadpoor-Baltork, V. Mirkhani, A. R. Khosropour, M. Moghadam, S. Tangestaninejad ,R.Kia, Adv.Synth.Catal., 2013, 355, 957-972, DOI: 10.1002/adsc.201200707.

6-S. Sithebe, R. S. Robinson, *Beilstein J. Org. Chem.*, 2014, 10, 1107-1113. DOI:10.3762/bjoc.10.109. 7-M. F. Amaral, D. R. Callejon, T. B. Riul, J. Braz. Chem. Soc., 2014, 25, 1907-1913, DOI: 10.5935/0103-5053.20140196.

8- A. K. Sahoo, T. Oda, Y. Nakao, T. Hiyama, Adv. Synth. Catal., 2005, 347, 413-417, DOI: 10.1002/adsc.200404188.

9-P. K. Mandali, D. K. Chand, Catal.Commun. 2013, 31, 16-20, DOI:10.1016/j.catcom.2012.10.020.

10- G. Zhang, Y. Luan, X. Han, Y. Wang, X. Wen, C. Ding, J. Gao, *Green Chem.*, 2013, 15, 2081-2085, DOI: 10.1039/C3GC40645H.

11-M. Gholinejad, M. Eskandari, J. M. Sansano, *J. Mol. Struct.*, 2025, 141364, DOI:10.1016/j.molstruc.2025.141364.

12- L. Li, J. Wang, C. Zhou, R. Wang, M. Hong, *Green Chem.*, 2011, 13, 2071-2077, DOI:10.1039/C1GC15312A.

.
